# Organolithium aggregation as a blueprint to construct polynuclear lithium nickelate clusters†

**DOI:** 10.1039/d3cc01729j

**Published:** 2023-04-28

**Authors:** Andryj M. Borys, Eva Hevia

**Affiliations:** a Departement für Chemie, Biochemie und Pharmazie, Universität Bern Bern 3012 Switzerland andryj.borys-smith@unibe.ch eva.hevia@unibe.ch

## Abstract

By exploiting the high aggregation of aliphatic lithium acetylides, here we report the synthesis and structural analysis of polynuclear lithium nickelate clusters in which up to 10 equivalents of organolithium can co-complex per Ni(0) centre. Exposure of the Ni(0)-ate clusters to dry air provides an alternative route to homoleptic Ni(ii)-ates.

## Abstract 2

Exploiting the high aggregation of organolithium complexes, a new structurally diverse family of lithium nickelate clusters has been uncovered, which can be accessed by treating Ni(COD)_2_ with a large excess of aliphatic lithium acetylides.

## Graphical abstract

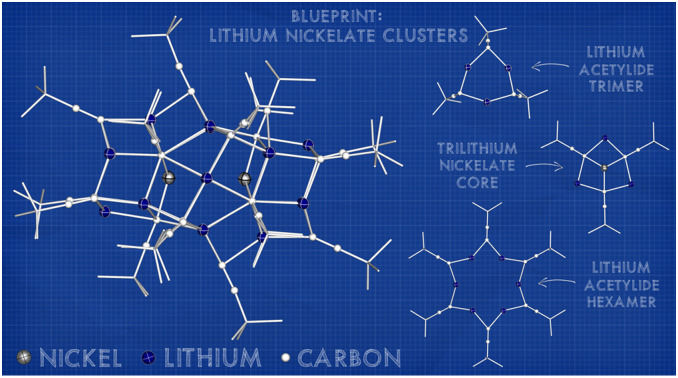

The aggregation and solvation of organo-alkali-metal compounds plays a crucial role in influencing their reactivity and selectivity.^1–3^ Typically, polar ethereal solvents such as THF or polydendate amine donors such as TMEDA (*N,N,N*′,*N*′-tetramethylethylenediamine)^4^ or PMDETA (*N,N,N*′,*N*′,*N*′′-pentamethyldiethylenetriamine)^5^ are employed to break down oligomeric aggregates into kinetically activated monomers or dimers, which exhibit enhanced reactivity, particularly towards deprotonative metalations. Contrastingly, there have been limited applications to date that take advantage of the high aggregation of organo-alkali-metal compounds. Numerous studies and reviews have been documented that assess the aggregation of organo-alkali-metal compounds in solution and the solid-state,^1–3,6^ yet lithium acetylides are comparatively underexplored in this domain.^7–9^ In 1987, Weiss and co-workers reported that ^*t*^Bu–C≡C–Li can form THF-solvated tetrameric and dodecameric aggregates in the solid-state simply depending on the crystallisation conditions employed.^10^ Additional factors such as London dispersion interactions may also play an overlooked role, as evidenced by the dimeric aggregate of Ph–C≡C–Li with TMPDA (*N*,*N*,*N*′*,N*′-tetramethylpropanediamine)^11^*versus* the tetrameric aggregate of Ph–C≡C–Li with TMHDA (*N*,*N*,*N*′,*N*′-tetramethylhexanediamine)^12^ in which the diamine donors differ only in the backbone chain length.

Beyond their well-established applications in deprotonative metalation, metal-halogen exchange and nucleophilic addition or substitution reactions, organo-alkali-metal compounds can also serve as anionic ligands towards a range of secondary metals (s-, p-, d- and f-block) to give rise to heterobimetallic complexes.^13,14^ In this context, the coordination of polar organometallics to Ni(0)-olefin complexes can afford highly reactive heterobimetallic nickelates,^15^ and we have recently assessed the rich co-complexation chemistry of Ni(COD)_2_ (COD = 1,5-cyclooctadiene) with various organo-alkali-metal compounds such as aryl-lithiums and lithium acetylides.^16–19^ In several cases, additional molecules of organolithium are readily incorporated within the nickelate structure, but not coordinated directly to Ni(0), and this feature has also been observed with lithium halides^17^ and alkali-metal alkoxides.^17,20^ Lithium nickelates with Li:Ni ratios of 1 : 1, 2 : 1 and 3 : 1 have now been documented,^15–19^ and we sought to exploit the high aggregation ability of aliphatic lithium acetylides to access new classes of lithium nickelates with higher Li : Ni ratios.

We began by investigating the aggregation of ^*t*^Bu–C≡C–Li in the absence of strong donor solvents or Lewis bases.^8,21^ Crystallisation of ^*t*^Bu–C≡C–Li from Et_2_O and pentane afforded single crystals identified as a decameric (10 units) aggregate, [Li_10_(Et_2_O)_4_(C≡C–^*t*^Bu)_10_] (1, Fig. 1). The solid-state structure consists of four linearly-fused heterocubanes in which the terminal Li atoms (Li1 and Li2) are solvated by Et_2_O – this bears similar structural properties to the dodecameric (12 units) aggregate reported by Weiss, [Li_12_(THF)_4_(C≡C–^*t*^Bu)_12_], which instead contains five linearly-fused heterocubanes despite the presence of the stronger donor solvent THF.^10^^1^H DOSY NMR spectroscopy studies support that large aggregates [Li_n_(THF)_4_(C≡C–^*t*^Bu)_n_] (where *n* = 10 or 12) are retained in weakly coordinating solvent systems (C_6_D_6_ + 1 equiv. THF-d_8_), whilst in bulk THF-d_8_, ^*t*^Bu–C≡C–Li is tetrameric (see the ESI† for more details). This is consistent with literature reports that have employed cryoscopy measurements^8^ or low-temperature ^13^C NMR spectroscopy in tandem with isotopic labelling to determine the aggregation of ^*t*^Bu–C≡C–Li in THF solutions.^7^

**Fig. 1:**
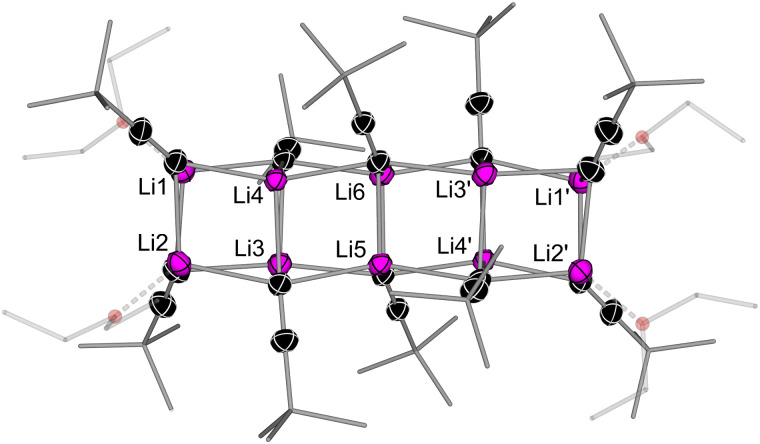
Molecular structure of [Li_10_(Et_2_O)_4_(C≡C–^*t*^Bu)_10_] (1). Thermal ellipsoids shown at 30% probability. Hydrogen atoms omitted and ^*t*^Bu groups and coordinated Et_2_O shown as wireframes for clarity.

With this knowledge in hand, we then went on to assess the reactivity of ^*t*^Bu–C≡C–Li with Ni(COD)_2_ in weakly coordinating solvent systems. Room temperature treatment of Ni(COD)_2_ with excess ^*t*^Bu–C≡C–Li (optimised with 9 equivalents) in Et_2_O (Fig. 2a), followed by crystallisation from pentane at −30 °C afforded emerald green crystals identified as a solvent-free, 9 : 1 lithium nickelate cluster, [Li_9_Ni(C≡C–^*t*^Bu)_9_]_2_ (2, Fig. 2b). This unique heterobimetallic cluster is constructed from three distinct building blocks (Fig. 2c): (i) a cyclotrimeric lithium acetylide ‘end cap’; (ii) a distorted-planar tri-lithium nickelate; and (iii) a cyclohexameric lithium acetylide core, which brings two nickelate units together to form a 20-metal-centred cluster. The Li⋯Ni distances in the tri-lithium nickelate unit range from 2.64(1) to 2.654(7) Å, which is outside the sum of the covalent radii (2.52 Å)^22^ and longer than observed in Li_3_(TMEDA)_3_Ni(C≡C–Ph)_3_ [2.487(4)–2.512(3) Å] in which the Li⋯Ni interactions were found to be repulsive in nature despite their close proximity.^18^ The three unique environments observed in the solid-state are also evidenced in the ^1^H NMR spectrum to give three signals of equal intensity at *δ* 1.79, 1.44 and 1.42. The ^7^Li NMR spectrum of 2 shows two signals in an approximate 1 : 2 ratio at *δ* 1.47 and 0.10, which can be assigned to [Li_3_Ni(C≡C–^*t*^Bu)_3_] and [^*t*^Bu–C≡C–Li]_*n*_, respectively (*c.f. δ* 0.52 for the free lithium acetylide). ^1^H DOSY NMR spectroscopy reveals that only one species exists in toluene-d_8_ solution but suggests that [Li_9_Ni(C≡C–^*t*^Bu)_9_]_2_ (2) dissociates to “Li_9_Ni(C≡C–^*t*^Bu)_9_” (see the ESI† for further details).

**Fig. 2:**
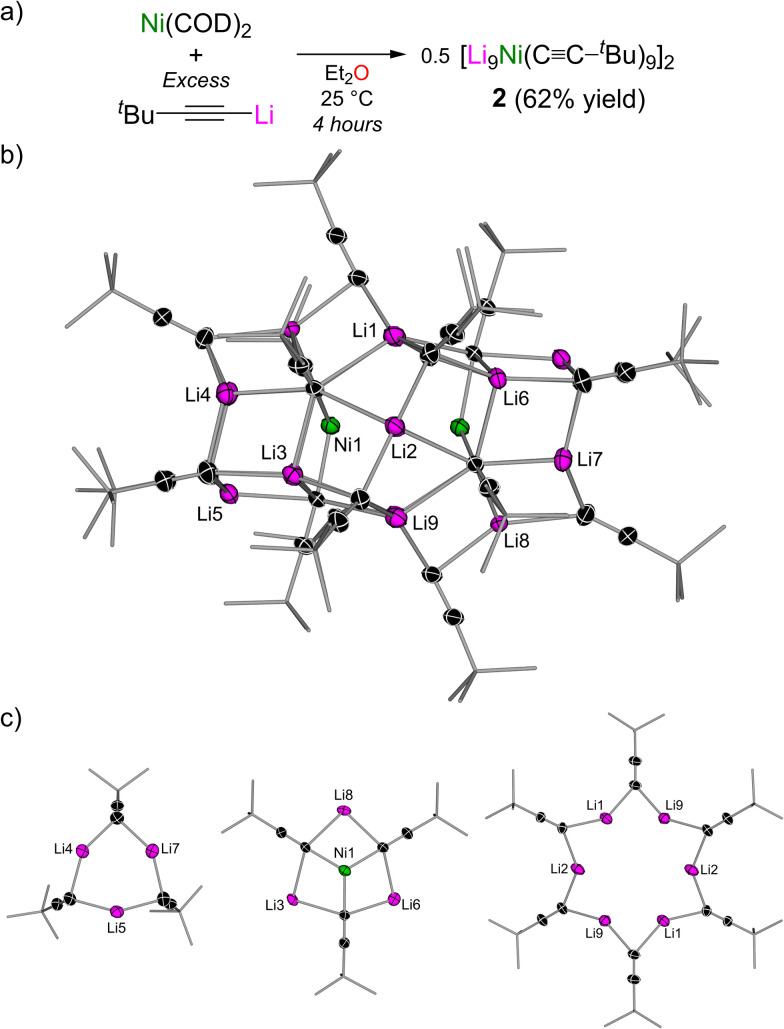
(a) Synthesis of [Li_9_Ni(C≡C–^*t*^Bu)_9_]_2_ (2). (b) Molecular structure of 2. Thermal ellipsoids shown at 30% probability. Hydrogen atoms omitted and ^*t*^Bu groups shown as wireframes for clarity. (c) Building blocks of 2.

The absence of any coordinating solvents in the solid-state structure of 2 is particularly surprising and illustrates that the acetylide carbanion is a more suitable donor than Et_2_O (both from an electronic and steric consideration), which may also explain the high aggregation of the free lithium acetylide in the absence of strong donor solvents or Lewis bases. Compound 2 is a rare example of a polynuclear organometallic cluster containing two distinct metals and to the best of our knowledge represents a new structural motif and stoichiometry in heterobimetallic ‘ate’ chemistry.

Extending this simple synthetic strategy to Me_3_Si–C≡C–Li (optimised with 10 equivalents) (Fig. 3a) instead led to the isolation of Li_10_(Et_2_O)_3_Ni(C≡C–SiMe_3_)_10_ (2, Fig. 3b), which grew as large orange crystals from Et_2_O and (Me_3_Si)_2_O. Unlike [Li_9_Ni(C≡C–^*t*^Bu)_9_]_2_ (2), which contains two tri-lithium nickelate units, the 10 : 1 Li:Ni cluster 3 contains a single tetra-lithium nickelate core (Fig. 3c), which is decorated by six additional lithium acetylides. This tetrahedral Ni(0)-ate motif has been proposed in the closely related [K_4_Ni(C≡C–H)_4_] species, which can be obtained by potassium metal reduction of [K_2_Ni(C≡C–H)_4_].^23^ This compound however was reported to be insoluble even in liquid NH_3_, in contrast to the high hydrocarbon solubility of Li_10_(Et_2_O)_3_Ni(C≡C–SiMe_3_)_10_ (3). Compound 3 therefore represents the first structurally characterised Ni(0) complex coordinated by four carbanions. The requirement however for the additional lithium acetylides to stabilise the “Li_4_Ni(C≡C–SiMe_3_)_4_” motif is supported by our previous reports that showed that the treatment of Ni(COD)_2_ with 3 equivalents of Me_3_Si–C≡C–Li in the presence of TMEDA does not give the homoleptic tri-lithium nickelate Li_3_(TMEDA)_3_Ni(C≡C–SiMe_3_)_3_ but instead gives a dinickel complex in which the lithium acetylide coordinates in a side-on fashion to modulate the electron-density at the electron-rich nickel centers.^18^

**Fig. 3:**
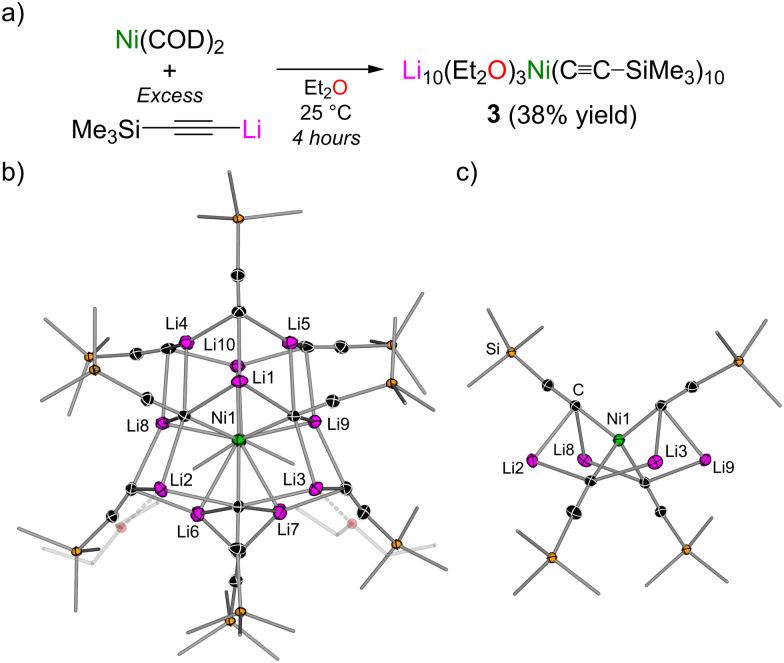
(a) Synthesis of Li_10_(Et_2_O)_3_Ni(C≡C–SiMe_3_)_10_ (3). (b) Molecular structure of 3. Thermal ellipsoids shown at 30% probability. Hydrogen atoms and one coordinated Et_2_O omitted and Me_3_Si groups shown as wireframes for clarity. Only one molecule in the asymmetric unit is shown. (c) Simplified view of the tetra-lithium nickelate core of 3.

^1^H DOSY NMR spectroscopy indicates that Li_10_(Et_2_O)_3_Ni(C≡C–SiMe_3_)_10_ (3) is fully retained in toluene-d_8_ solution. In THF-d_8_, however, the lithium nickelate cluster dissociates to “Li_4_(THF)_*n*_Ni(C≡C–SiMe_3_)_4_” and the free lithium acetylide (Me_3_Si–C≡C–Li)_*x*_(THF)_*y*_, as supported by ^1^H and ^7^Li NMR spectroscopy and confirmed by two independent species that do not co-diffuse by ^1^H DOSY NMR spectroscopy (see the ESI† for full details). ^1^H DOSY NMR spectroscopy studies on Me_3_Si–C≡C–Li indicate that it forms lower aggregates when compared to ^*t*^Bu–C≡C–Li in both weakly coordinating solvent systems (hexamer) and bulk THF (dimer),^24^ which likely influences the final lithium nickelate cluster obtained (*i.e.*2*vs.*3).

Polynuclear transition-metal clusters are often sensitive to the crystallisation conditions employed^25,26^ and this was also observed to be true for the lithium nickelate clusters. Whilst Li_10_(Et_2_O)_3_Ni(C≡C–SiMe_3_)_10_ (3) was the only species that could be crystallographically identified when treating Ni(COD)_2_ with Me_3_Si–C≡C–Li, regardless of the stoichiometry and crystallisation conditions, the isostructural ^*t*^Bu–C≡C–Li analogue Li_10_(Et_2_O)_3_Ni(C≡C–^*t*^Bu)_10_ (4) could also be crystallographically characterised (see the ESI† for the full structure) by simply switching to Et_2_O and (Me_3_Si)_2_O as crystallisation solvents instead of pentane that was used to crystallise [Li_9_Ni(C≡C–^*t*^Bu)_9_]_2_ (2). Additionally, when treating Ni(COD)_2_ with lower equivalents of ^*t*^Bu–C≡C–Li (5 equivalents), 26-metal-centred cluster [Li_11_(Et_2_O)Ni_2_(C≡C–^*t*^Bu)_11_]_2_ (5) could be isolated and characterised by single-crystal X-ray diffraction (see the ESI† for full structure). The molecular structure of 5 shows similar features to [Li_9_Ni(C≡C–^*t*^Bu)_9_]_2_ (2) and is constructed from well-defined cyclotrimeric lithium acetylide and distorted-planar tri-lithium nickelate building blocks (see Fig. 2c), but also contains regions in which the Li cations are occupationally disordered across two or more positions. Several other aliphatic lithium acetylides were also explored; cycloalkyl (propyl, pentyl, and hexyl) lithium acetylides all gave insoluble and intractable solids however, whilst ^*i*^Pr–C≡C–Li (10 equivalents) afforded mixed acetylide/alkoxide cluster [Li_10_(Et_2_O)_2_Ni(C≡C–^*i*^Pr)_8_(C≡C–Me_2_O)]_2_ (6), albeit in low yields (see the ESI† for the full structure). Attempts to prepare or crystallise the polynuclear lithium nickelate clusters from THF were unsuccessful, supporting the crucial role of aggregation in the construction of these complexes.

Terminal acetylenes and metal acetylides can undergo homocoupling in the presence of transition-metal catalysts (*e.g.* Cu, Mn, and Fe) and oxidants to afford the corresponding 1,3-diynes.^27–29^ Several examples using Ni-catalysts have been reported^30^ and we therefore considered whether the simple lithium acetylide/Ni(COD)_2_ system was also catalytically competent. Exposure of ^*t*^Bu–C≡C–Li to dry air in the presence of 5 mol% Ni(COD)_2_ afforded the corresponding 1,3-diyne, ^*t*^Bu–C≡C–C≡C–^*t*^Bu in a respectable 57% yield after 2 hours (Scheme 1), whilst no homocoupling is observed in the absence of a Ni catalyst. Although lithium nickelates have been shown to be key intermediates in the Ni(COD)_2_ catalysed cross-coupling of aryl ethers with phenyl-lithium,^16^ no reactivity was observed under stoichiometric or catalytic conditions between 2 or 3 and 2-methoxynaphthalene.

**Scheme 1:**
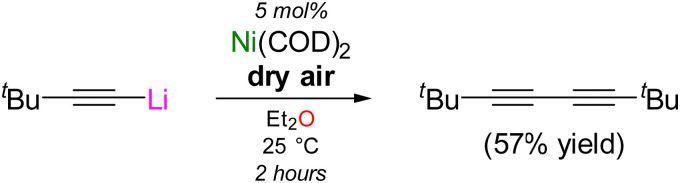
Oxidative homocoupling of ^*t*^Bu–C≡C–Li catalysed by Ni(COD)_2_ in the presence of dry air. Yields refer to spectroscopic yields determined using hexamethylbenzene as an internal standard.

Since a large excess of the lithium acetylide is present with respect to Ni(COD)_2_ under these catalytic conditions, it could be hypothesised that polynuclear clusters such as [Li_9_Ni(C≡C–^*t*^Bu)_9_]_2_ (2) and Li_10_(Et_2_O)_3_Ni(C≡C–SiMe_3_)_10_ (3) initially form and are involved in the reaction. Supporting this claim, exposure of lithium nickelate clusters 2 and 3 to dry air for 1 hour resulted in a loss of colour and oxidation to the corresponding homoleptic Ni(ii)-ates, [Li_2_(Et_2_O)Ni(C≡C–^*t*^Bu)_4_]_2_ (7, 55% yield) and Li_2_(Et_2_O)_2_Ni(C≡C–SiMe_3_)_4_ (8, 34% yield) (Fig. 4a). The consumption of residual lithium acetylide *via* oxidative homocoupling means that no additional organolithium co-complexation is observed in compounds 7 and 8, which is in contrast to lithium nickelate clusters 2–6 (*vide supra*) and related lithium ferrates prepared *via* a salt-metathesis route using excess Me_3_Si–C≡C–Li.^31^

**Fig. 4:**
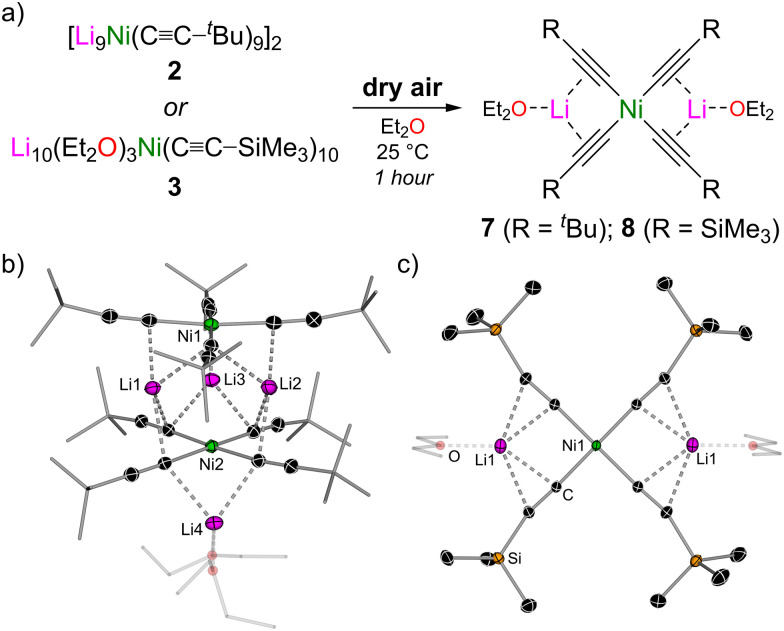
(a) Synthesis of [Li_2_(Et_2_O)Ni(C≡C–^*t*^Bu)_4_]_2_ (7) and Li_2_(Et_2_O)_2_Ni(C≡C–SiMe_3_)_4_ (8). (b) Molecular structure of 7. Thermal ellipsoids shown at 30% probability. Hydrogen atoms omitted and ^*t*^Bu groups and coordinated Et_2_O shown as wireframes for clarity. (c) Molecular structure of 8. Thermal ellipsoids shown at 50% probability. Hydrogen atoms omitted and coordinated Et_2_O shown as wireframes for clarity.

This transformation is unique from the perspective of accessing homoleptic Ni(ii)-ates since reported synthetic routes are often low-yielding or not possible *via* traditional salt-metathesis routes with NiX_2_ precursors (X = halide or acetylacetonate) due to the inherent instability of the neutral NiR_2_ intermediates in the absence of suitable ligands,^32,33^ and the use of bespoke polydendate ligands is generally necessary to reliably prepare Li_2_NiR_4_ complexes (where R = alkyl or aryl).^32,34^ This simple oxidation route starting from a readily accessible Ni(0) precursor therefore offers an alternative route to access these heterobimetallic complexes that may find further applications in catalysis and other areas of organometallic chemistry.

Compound 7 exists as a dimer in the solid-state in which two square planar Ni(C≡C–^*t*^Bu)_4_ units are offset and rotated by 45° (Fig. 4b). Three unsolvated Li atoms (Li1, Li2 and Li3) are sandwiched between the two Ni(C≡C–^*t*^Bu)_4_ planes, whilst one Li atom (Li4) sits below one of the Ni(C≡C–^*t*^Bu)_4_ planes and is further coordinated by two molecules of Et_2_O. This asymmetric dimeric motif contrasts with [Li_2_(THF)_2_Ni(CH_3_)_4_]_2_ that exists as a *D*_4h_ symmetric dimer in the solid-state where all four Li atoms are sandwiched between two Ni(CH_3_)_4_ planes.^34^ Despite the similar electronic and steric properties of ^*t*^Bu and Me_3_Si-substituents, compound 8 exists as a discrete monomer in the solid-state (Fig. 4c). The Li atoms lie in the same plane as the Ni center and four acetylide substituents and the Li⋯C contacts adopt a narrow range [2.210(1)–2.334(2) Å *vs.* 2.154(3)–2.542(3) Å for 7].

In conclusion, we have uncovered a new and structurally diverse family of polynuclear lithium nickelate clusters that can be readily accessed by treating Ni(COD)_2_ with aliphatic lithium acetylides in Et_2_O solution. Exposure of the Ni(0)-ates to dry air leads to the formation of the homoleptic Ni(ii)-ates with concomitant formation of the oxidative homocoupling product. This redox behaviour, along with the observation of higher order systems, hints at the possible role and applications of heterobimetallic nickelate clusters in homogenous catalysis.

We thank the SNSF (188573) and the Universität Bern for their generous sponsorship of this research.

## Conflicts of interest

There are no conflicts to declare.

## Supplementary Material

CC-059-D3CC01729J-s001

CC-059-D3CC01729J-s002
