# Supplementary material for: Organolithium aggregation as a blueprint to construct polynuclear lithium nickelate clusters
Source: Chem Commun (Camb). 2023 Apr 28;59(46):7032–5. doi: 10.1039/d3cc01729j (PMC10243118; doi:10.1039/d3cc01729j)
Supplement: CC-059-D3CC01729J-s001 [file CC-059-D3CC01729J-s001.pdf]

Electronic Supplementary Material (ESI) for ChemComm.
This journal is © The Royal Society of Chemistry 2023

Electronic Supplementary Information

Organolithium aggregation as a blueprint to construct polynuclear lithium nickelate clusters

Andryj M. Borys,[a]* Eva Hevia[a]*

[a] Departement für Chemie, Biochemie und Pharmazie, Universität Bern, 3012 Bern, Switzerland

Table of Contents

Experimental 3

General Considerations 3

Synthesis of ^{t}Bu–C≡C–Li (**1**) 3

Synthesis of $[Li_9Ni(C{\equiv}C–^tBu)_9]_2$ (**2**) 4

Synthesis of $Li_{10}(Et_2O)_3Ni(C{\equiv}C–SiMe_3)_{10}$ (**3**) 4

Synthesis of $Li_{10}(Et_2O)_3Ni(C{\equiv}C–^tBu)_{10}$ (**4**) 5

Synthesis of $[Li_{11}(Et_2O)Ni_2(C{\equiv}C–^tBu)_{11}]_2$ (**5**) 5

Synthesis of $[Li_{10}(Et_2O)_2Ni(C{\equiv}C–^iPr)_8(C{\equiv}C–Me_2O)]_2$ (**6**) 5

Synthesis of $Li_2(Et_2O)_nNi(C{\equiv}C–^tBu)_4$ (**7**) 6

Synthesis of $Li_2(Et_2O)_2Ni(C{\equiv}C–SiMe_3)_4$ (**8**) 6

Oxidative Homocoupling Experiments 7

DOSY NMR Spectroscopy 9

^{t}Bu–C≡C–Li in C_6D_6 (+ 1 eq. THF-d_8) 9

^{t}Bu–C≡C–Li in THF-d_8 10

Me_3Si–C≡C–Li in C_6D_6 (+ 1 eq. THF-d_8) 11

Me_3Si–C≡C–Li in THF-d_8 12

$[Li_9Ni(C{\equiv}C–^tBu)_9]_2$ (**2**) in Toluene-d_8 13

$[Li_9Ni(C{\equiv}C–^tBu)_9]_2$ (**2**) in THF-d_8 14

$Li_{10}(Et_2O)_3Ni(C{\equiv}C–SiMe_3)_{10}$ (**3**) in Toluene-d_8 16

$Li_{10}(Et_2O)_3Ni(C{\equiv}C–SiMe_3)_{10}$ (**3**) in THF-d_8 17

$Li_{10}(Et_2O)_3Ni(C{\equiv}C–^tBu)_{10}$ (**4**) in Toluene-d_8 20

$[Li_{11}(Et_2O)Ni_2(C{\equiv}C–^tBu)_{11}]_2$ (**5**) in Toluene-d_8 20

$[Li_{10}(Et_2O)_2Ni(C{\equiv}C–^iPr)_8(C{\equiv}C–Me_2O)]_2$ (**6**) in Toluene-d_8 21

Variable Temperature NMR Spectroscopy 22

X-Ray Crystallography 25

Molecular Structure of $[Li_{10}(Et_2O)_4(C{\equiv}C–^tBu)_{10}]$ (**1**) 31

Molecular Structure of $[Li_9Ni(C{\equiv}C–^tBu)_9]_2$ (**2**) 32

Molecular Structure of $Li_{10}(Et_2O)_3Ni(C{\equiv}C–SiMe_3)_{10}$ (**3**) 34

Molecular Structure of $Li_{10}(Et_2O)_3Ni(C{\equiv}C–^tBu)_{10}$ (**4**) 36

Molecular Structure of $[Li_{11}(Et_2O)Ni_2(C{\equiv}C–^tBu)_{11}]_2$ (**5**) 38

Molecular Structure of $[Li_{10}(Et_2O)_2Ni(C{\equiv}C–^iPr)_8(C{\equiv}C–Me_2O)]_2$ (**6**) 38

Molecular Structure of $[Li_2(Et_2O)_2Ni(C{\equiv}C–^tBu)_4]_2$ (**7**) 39

Molecular Structure of $Li_2(Et_2O)_2Ni(C{\equiv}C–SiMe_3)_4$ (**8**) 40

NMR Spectra of Reported Compounds 41

References 50

Experimental

General Considerations

All manipulations were carried out under an inert atmosphere of argon using standard Schlenk line[1,2] or glovebox techniques (MBraun UNILab Pro ECO, <0.1 ppm H_2O and O_2). Due to the extreme air, moisture, and often temperature sensitivity of described compounds, rigorously inert conditions must be maintained to allow for the isolation of crystalline and pure samples. All manipulations, except for the preparation of organolithium starting materials, must avoid the use of Teflon-coated stir bars and Teflon cannulae, and glass-coated stir bars should be used. Specific experimental details can be found below. THF was dried and distilled from Na/benzophenone and stored over 4 Å molecular sieves, then further dried and vacuum distilled over $NaK_{2.8}$ or a sodium mirror. Hexane, pentane, Et_2O, toluene and benzene were pre-dried using a MBraun MBSPS 5, then further dried and vacuum distilled over $NaK_{2.8}$ or a sodium mirror, and stored over 4 Å molecular sieves. $(Me_3Si)_2O$ was degassed, dried and distilled over CaH_2 and stored over 4 Å molecular sieves. THF-d_8,Tol-d_8 and C_6D_6 were dried and vacuum distilled over $NaK_{2.8}$ and stored over 4 Å molecular sieves in a glovebox prior to use. Me_3Si–C≡C–Li was prepared as previously reported.[3] Ni(COD)$_2$ was purchased from commercial sources (Sigma Aldrich or Strem). All other reagents were used as supplied without further purification.

NMR spectra were recorded on Bruker Avance III HD 300 MHz or 400 MHz spectrometers at 300 K unless otherwise specified. ^{1}H NMR spectra were referenced internally to the corresponding residual *protio* solvent peaks. CHN elemental microanalyses were performed on a Flash 2000 Organic Elemental Analyser (Thermo Scientific). Samples were prepared and crimped in tin capsules in an argon filled glovebox. Analyses were performed in triplicate, and reference standards (e.g. nicotinamide) were measured prior to use as controls.

Synthesis of ^{t}Bu–C≡C–Li (1)

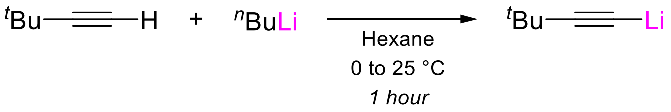

3,3-Dimethyl-1-butyne (2.5 mL, 20.3 mmol) was dissolved in hexane and cooled to 0 °C. ^{n}BuLi (1.6 M, 12.7 mL, 20.3 mmol) was added dropwise and the resulting colourless suspension was warmed to room temperature and stirred for 1 hour. The colourless solids were collected on a filter frit, washed with hexane (2 × 10 mL), and dried *in vacuo*. Yield – 1.57 g (88%).

Single crystals suitable for X-ray diffraction were grown from Et_2O and pentane at -30 °C.

^{1}H NMR (300.1 MHz, 5:1 C_6D_6/THF-d_8): δ 1.28 {s, 9H, C(C$\underline{H_3}$)$_3$}.

^{7}Li NMR (116.6 MHz, 5:1 C_6D_6/THF-d_8): δ 0.52 {br}.

^{13}C{^{1}H} NMR (75.5 MHz, 5:1 C_6D_6/THF-d_8): δ 123.4 {^{t}Bu–C≡$\underline{C}$–Li}, 115.9 {br, ^{t}Bu–$\underline{C}$≡C–Li}, 33.4 {C($\underline{C}$H$_3$)$_3$}, 28.9 {$\underline{C}$(CH$_3$)$_3$}.

Synthesis of [$Li_9Ni(C{\equiv}C{-}{}^tBu)_9]_2$ (2)

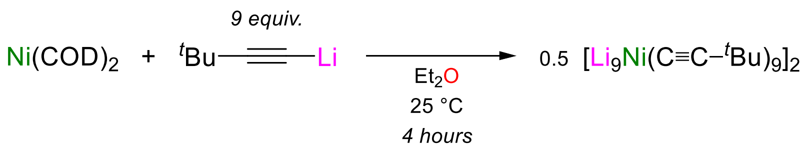

$Ni(COD)_2$ (28 mg, 0.1 mmol) and $^tBu{-}C{\equiv}C{-}Li$ (79 mg, 0.9 mmol) were combined in Et_2O (2.5 mL) and stirred at room temperature for 4 hours. The dark green solution was evaporated to dryness and the residues were extracted into pentane (1.5 mL), filtered through a glass wool plug and stored at -30 °C. After 1 week, the dark green crystals were separated from the supernatant and dried under argon. Yield – 53 mg (62%).

^{1}H NMR (400.1 MHz, Tol-d_8): δ 1.79 {s, 54H}, 1.44 {s, 54H}, 1.42 {s, 54H}.

^{7}Li NMR (155.5 MHz, Tol-d_8): δ 1.47 {s}, 0.10 {br}.

N.B. It was not possible to confidently identify or assign signals in the $^{13}C\{^1H\}$ NMR spectrum.

Elemental Analysis: Calculated for $C_{108}H_{162}Li_{18}Ni_2$: C, 76.18; H, 9.59. Found: C, 76.25; H, 9.30.

Synthesis of $Li_{10}(Et_2O)_3Ni(C{\equiv}C{-}SiMe_3)_{10}$ (3)

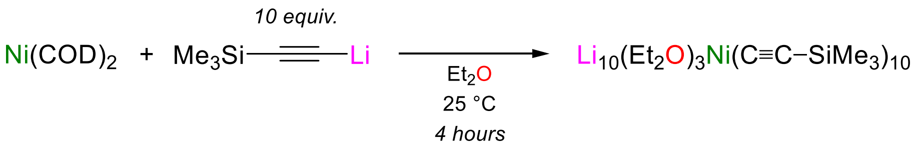

$Ni(COD)_2$ (28 mg, 0.1 mmol) and $Me_3Si{-}C{\equiv}C{-}Li$ (104 mg, 1.0 mmol) were combined in Et_2O (2.5 mL) and stirred at room temperature for 4 hours. The bright orange solution was evaporated to dryness and the residues were extracted into $(Me_3Si)_2O$ (1 mL) and Et_2O (0.2 mL), filtered through a glass wool plug and stored at -30 °C. After 2 weeks, the large orange crystals were separated from the supernatant and dried under argon. Yield – 56 mg (38%).

^{1}H NMR (400.1 MHz, Tol-d_8): δ 3.40 {q, Et_2O}, 1.17 {t, Et_2O}, 0.57 {s}, 0.54 {br}, 0.37 {br}, 0.33 {s}, 0.11 {$(Me_3Si)_2O$}.

^{7}Li NMR (155.5 MHz, Tol-d_8): δ 0.62 {br}.

N.B. It was not possible to confidently identify or assign signals in the $^{13}C\{^1H\}$ NMR spectrum.

Elemental Analysis: Calculated for $C_{68}H_{138}Li_{10}NiO_4Si_{12}$: C, 55.00; H, 9.37. Found: C, 54.39; H, 9.29.

N.B. NMR spectroscopy and elemental analysis consistent with 3 molecules of coordinated Et_2O and 1 molecule of $(Me_3Si)_2O$, as observed in the solid-state structure.

Synthesis of $Li_{10}(Et_2O)_3Ni(C{\equiv}C{-}^tBu)_{10}$ (4)

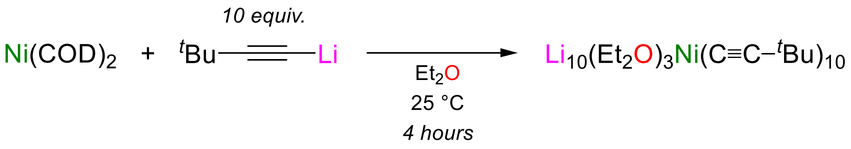

$Ni(COD)_2$ (28 mg, 0.1 mmol) and ^{t}Bu–C≡C–Li (88 mg, 1.0 mmol) were combined in Et_2O (2.5 mL) and stirred at room temperature for 4 hours. The deep green solution was evaporated to dryness and the residues were extracted into $(Me_3Si)_2O$ (1 mL) and Et_2O (0.2 mL), filtered through a glass wool plug and stored at -30 °C. After 1 week, orange single crystals suitable for X-ray diffraction were obtained. Crystalline samples were plagued with green $[Li_9Ni(C{\equiv}C{-}^tBu)_9]_2$ and could therefore not be isolated in analytically pure form.

^{1}H NMR (400.1 MHz, Tol-d_8): δ 3.27 {q, Et_2O}, 1.83–1.67 {br, 27H}, 1.46 {br, 9H}, 1.38 {s, 63H}, 1.11 {t, Et_2O}.

^{7}Li NMR (155.5 MHz, Tol-d_8): see Spectra S9.

Synthesis of $[Li_{11}(Et_2O)Ni_2(C{\equiv}C{-}^tBu)_{11}]_2$ (5)

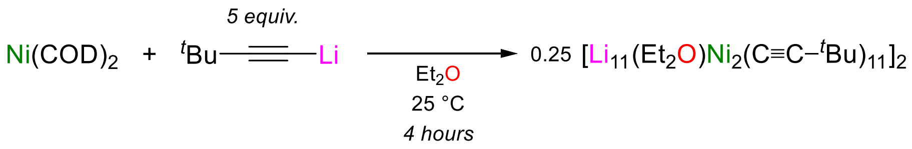

$Ni(COD)_2$ (110 mg, 0.4 mmol) and ^{t}Bu–C≡C–Li (176 mg, 2.0 mmol) were combined in Et_2O (5 mL) and stirred at room temperature for 4 hours. The dark brown solution was evaporated to dryness and the residues were extracted into $(Me_3Si)_2O$ (1 mL) and Et_2O (0.5 mL), filtered through a glass wool plug and stored at -30 °C. After 1 week, red single crystals suitable for X-ray diffraction were obtained. Yield – 25 mg (11% based on $Ni(COD)_2$).

N.B. It was not possible to confidently assign signals in the ^{1}H, ^{7}Li or ^{13}C{^{1}H} NMR spectrum. See Spectra S10–11 for ^{1}H and ^{7}Li NMR spectra.

Elemental Analysis: Calculated for $C_{140}H_{218}Li_{22}Ni_4O_2$: C, 72.46; H, 9.47. Found: C, 71.33; H, 8.65.

Synthesis of $[Li_{10}(Et_2O)_2Ni(C{\equiv}C{-}^iPr)_8(C{\equiv}C{-}Me_2O)]_2$ (6)

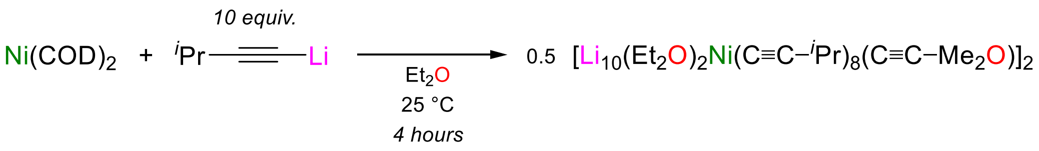

3-Methyl-1-butyne (103 µL, 1.0 mmol) was dissolved in Et_2O (1 mL) and cooled to -30 °C. $LiCH_2SiMe_3$ (94 mg, 1.0 mmol) was added dropwise as a chilled solution in Et_2O (1.5 mL). After warming to room temperature and stirring for 15 minutes, $Ni(COD)_2$ (28 mg, 0.1 mmol) was added and the pale yellow solution was allowed to stir at room temperature for 4 hours. The resulting dark green solution was

evaporated to dryness and the residues were extracted into $(Me_3Si)_2O$ (0.5 mL) and Et_2O (0.5 mL), filtered through glass wool and stored at -30 °C. After 2 weeks, the dark green crystals were separated from the supernatant and dried under argon. Yield – 13 mg (15%).

N.B. It was not possible to confidently assign signals in the ^{1}H, ^{7}Li or ^{13}C{^{1}H} NMR spectrum. See Spectra S12–13 for ^{1}H and ^{7}Li NMR spectra.

Elemental Analysis: Calculated for $C_{106}H_{164}Li_{20}Ni_2O_6$; C, 71.10; H, 9.23. Found: C, 70.68; H, 9.05.

Synthesis of $Li_2(Et_2O)_nNi(C{\equiv}C{-}^tBu)_4$ (7)

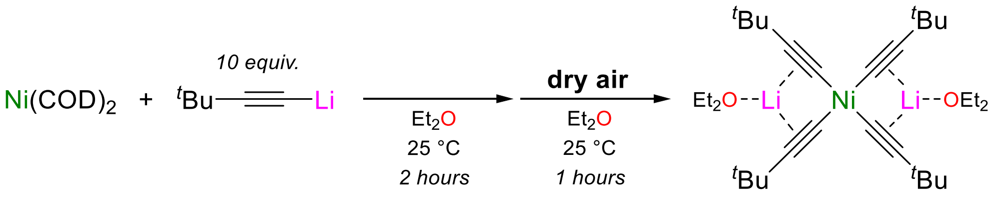

Ni(COD)$_2$ (55 mg, 0.2 mmol) and ^{t}Bu–C≡C–Li (172 mg, 2.0 mmol) were combined in Et_2O (5 mL) and stirred at room temperature for 2 hours. The dark brown solution was exposed to dry air through the attachment of a $CaCl_2$ filled drying tube and stirred at room temperature for 1 hour resulting in a colour change to red then pale yellow. The reaction mixture was evaporated to dryness then extracted into hexane (1 mL) and Et_2O (0.5 mL), filtered through a glass wool plug, and stored at -30 °C. After 48 hours, colourless crystals of **7** were separated from the supernatant, washed with cold pentane (2 × 0.5 mL) and dried under argon. Yield – 44 mg (55%).

The rational synthesis of compound **7** directly from Ni(II) precursors was also attempted, but no product could be reliably isolated.

^{1}H NMR (300.1 MHz, 5:1 C_6D_6/THF-d_8): δ 1.35 {s, 36H, C(C$\underline{H_3}$)$_3$}.

^{7}Li NMR (116.6 MHz, 5:1 C_6D_6/THF-d_8): δ 0.52 {s}.

^{13}C{^{1}H} NMR (75.5 MHz, 5:1 C_6D_6/THF-d_8): δ 120.2 {^{t}Bu–C≡$\underline{C}$–Ni}, 101.7 {^{t}Bu–$\underline{C}$≡C–Ni}, 33.6 {C($\underline{C}$H$_3$)$_3$}, 30.3 {$\underline{C}$(CH$_3$)$_3$}.

Elemental Analysis: Calculated for $C_{24}H_{36}Li_2Ni$: C, 72.59; H, 9.14. Found: C, 72.45; H, 8.93.

N.B. ^{1}H NMR spectroscopy and elemental analysis consistent with loss of coordinated Et_2O.

Synthesis of $Li_2(Et_2O)_2Ni(C{\equiv}C{-}SiMe_3)_4$ (8)

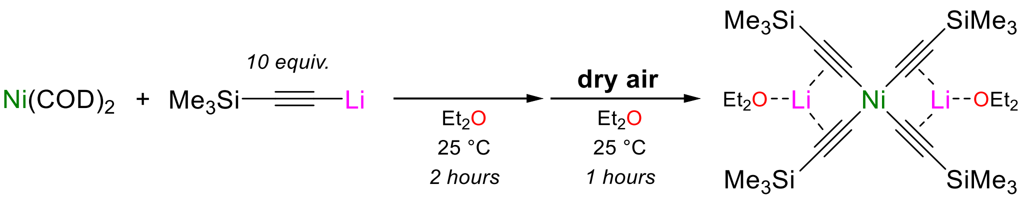

$Ni(COD)_2$ (55 mg, 0.2 mmol) and $Me_3Si–C≡C–Li$ (166 mg, 2.0 mmol) were combined in Et_2O (5 mL) and stirred at room temperature for 2 hours. The bright orange solution was exposed to dry air through the attachment of a $CaCl_2$ filled drying tube and stirred at room temperature for 1 hour resulting in a colour change to red then pale yellow. The reaction mixture was evaporated to dryness then extracted into $(Me_3Si)_2O$ (1 mL) and Et_2O (0.2 mL), filtered through a glass wool plug, and stored at -30 °C. After 1 week, colourless crystals of **8** were separated from the supernatant, washed with cold pentane (0.5 mL), and dried under argon. Yield – 42 mg (34%).

^{1}H NMR (300.1 MHz, 5:1 C_6D_6/THF-d_8): δ 3.25 {q, Et_2O}, 1.06 {t, Et_2O}, 0.14 {s, 36H, Si(C$\underline{H_3}$)$_3$}.

^{7}Li NMR (116.6 MHz, 5:1 C_6D_6/THF-d_8): δ 0.41 {s}.

^{13}C{^{1}H} NMR (75.5 MHz, 5:1 C_6D_6/THF-d_8): δ 147.8 {Me_3Si–C≡$\underline{C}$–Ni}, 116.4 {Me_3Si–$\underline{C}$≡C–Ni}, 66.2 (Et_2O), 15.8 (Et_2O), 2.1 {Si($\underline{C}H_3$)$_3$}.

N.B. It was not possible to obtain suitable elemental analysis data for **8** due to a persistent red microcrystalline impurity that contaminated isolated samples.

Oxidative Homocoupling Experiments

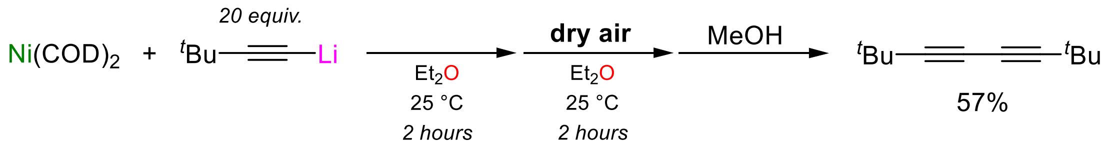

$Ni(COD)_2$ (14 mg, 0.05 mmol) and ^{t}Bu–C≡C–Li (86 mg, 1.0 mmol) were combined in Et_2O (5 mL) and stirred at room temperature for 2 hours. The dark brown solution was exposed to dry air through the attachment of a $CaCl_2$ filled drying tube and stirred at room temperature for 2 hours resulting in a colour change to pale brown. The reaction was quenched with MeOH (1 mL) and hexamethylbenzene (27 mg, 0.17 mmol) was added as an internal standard. An aliquot was taken, evaporated to dryness and redissolved in $CDCl_3$ for NMR spectroscopic analysis, which indicated a spectroscopic yield of 57% for 1,4-di-*tert*-butyl-1,3-diyne (Figure S1–2).

^{1}H NMR (300.1 MHz, $CDCl_3$): δ 1.25 {s, 18H, C(C$\underline{H_3}$)$_3$}.

^{13}C{^{1}H} NMR (75.5 MHz, $CDCl_3$): δ 86.3 {$\underline{C}$≡C}, 63.7 {C≡$\underline{C}$}, 30.6 {C($\underline{C}H_3$)$_3$}, 28.0 {$\underline{C}$(CH_3)$_3$}.

Analytical data for 1,4-di-*tert*-butyl-1,3-diyne in accordance with the literature.[4]

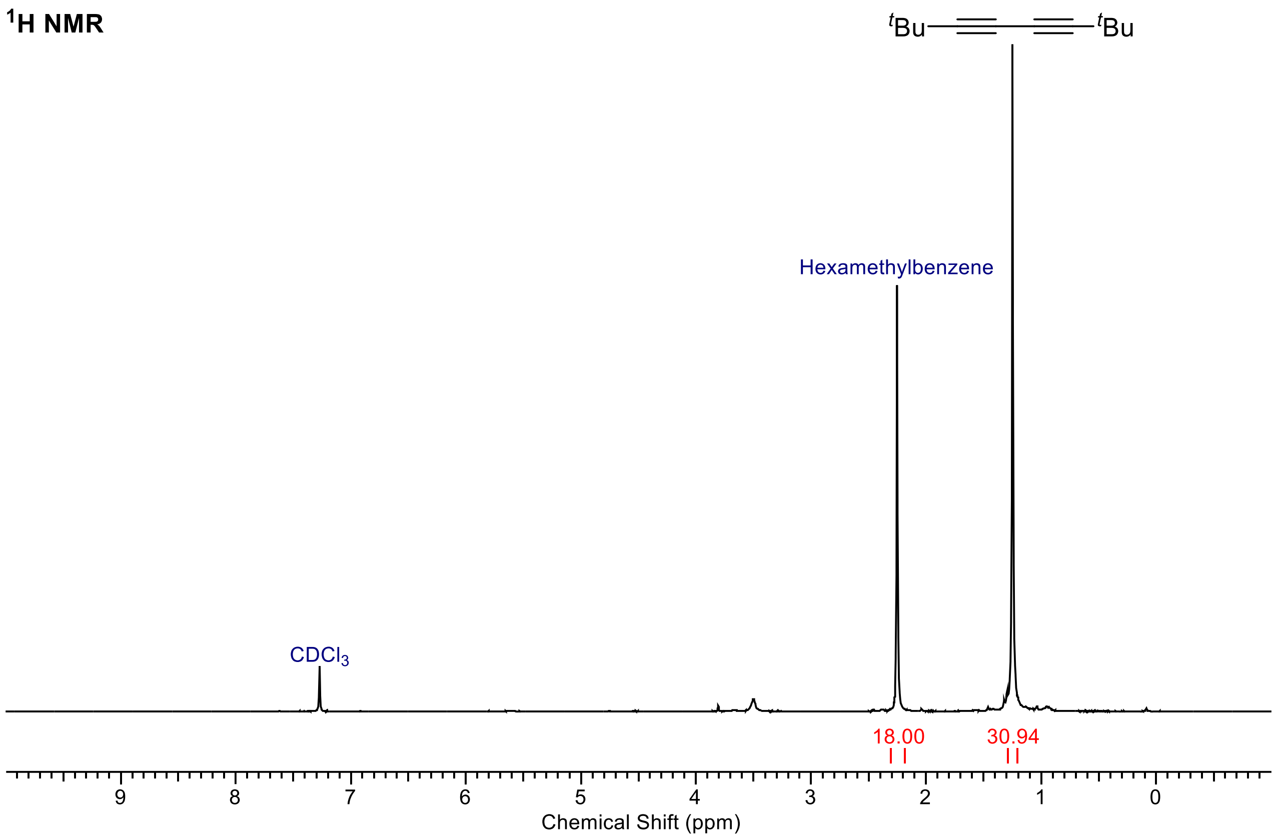

Figure S1: ^{1}H NMR spectrum of ^{t}Bu–C≡C–C≡C–^{t}Bu with hexamethylbenzene as an internal standard.

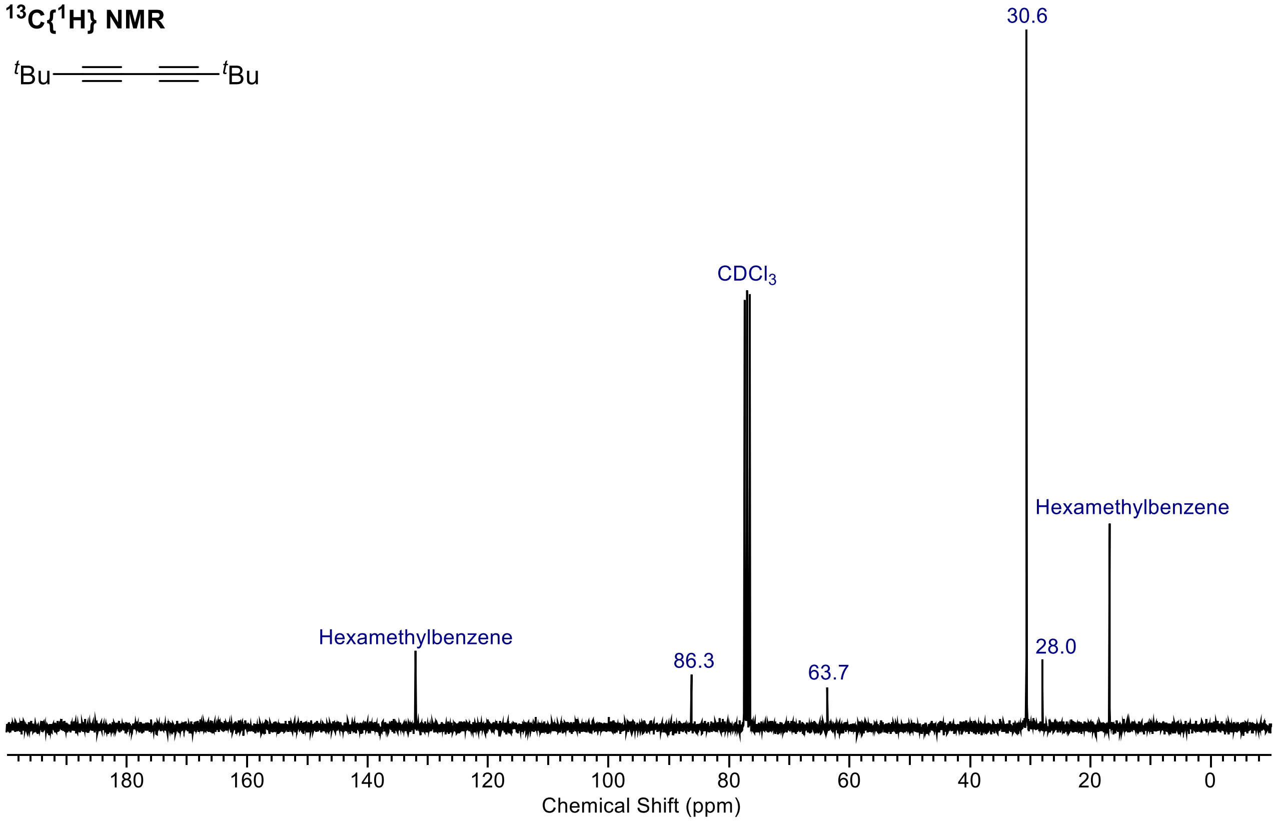

Figure S2: $^{13}C\{^{1}H\}$ NMR spectrum of ^{t}Bu–C≡C–C≡C–^{t}Bu with hexamethylbenzene as an internal standard.

Attempts to assess the quantity of $Li_2(solv)_nNi(C{\equiv}C{-}^tBu)_4$ or residual $^tBu{-}C{\equiv}C{-}Li$ by quenching the reaction with other electrophiles such as Me_3SiCl were inconclusive. Similarly, attempted oxidative homocoupling reaction with $Me_3Si{-}C{\equiv}C{-}Li$ were inconclusive since the formed $Me_3Si{-}C{\equiv}C{-}C{\equiv}C{-}SiMe_3$ product was observed to react with free $Me_3Si{-}C{\equiv}C{-}Li$ to give numerous unidentified side products.

DOSY NMR Spectroscopy

Estimated molecular weights (MW) were calculated from the diffusion coefficients established from the ^1H DOSY NMR spectrum using Stalke's external calibration curve (ECC)[5–7] method and using the residual proton signal of the deuterated solvent or 1,2,3,4-tetraphenylnaphthalene as internal standards unless otherwise specified. It should be emphasised that the current ECC method is not yet optimised for aggregates that have molecular weights above >600 g mol^{-1} due to the lack of suitable reference compounds. Nevertheless, the ^1H DOSY NMR spectra support that the lithium acetylides form large aggregates in the absence of bulk THF and that the lithium nickelate clusters are retained in non-donor solvents (toluene) whilst they dissociate in donor solvents (THF). Attempts to assess the solution-state aggregation of $^tBu{-}C{\equiv}C{-}Li$ in Et_2O solution using an internal calibration curve were unsuccessful due to overlap of the ^tBu signal with the *protio* solvent signal (both Et_2O or MTBE), and lack of suitable internal standards (i.e. high molecular weight and soluble in Et_2O).

$^tBu{-}C{\equiv}C{-}Li$ in C_6D_6 (+ 1 eq. THF-d_8)

$^tBu{-}C{\equiv}C{-}Li$ (1.3 mg, 0.015 mmol) was suspended in 0.5 mL of C_6D_6 and 1,2,3,4-tetraphenylnaphthalene (6.5 mg, 0.015 mmol) was added as an internal standard. One equivalent of THF-d_8 (1.2 µL) was added which enabled partial solubility of the lithium acetylide. The ^1H DOSY NMR spectrum (Figure S3) suggests that large aggregates exist in solution with an estimated molecular weight between 1032 g mol^{-1} (DSE = dissipated spheres and ellipsoids) and 1216 g mol^{-1} (Merge). This is consistent with a decameric aggregate $[Li_{10}(THF)_4(C{\equiv}C{-}^tBu)_{10}]$ (calculated MW = 1149.24 g mol^{-1}; +11% or -5% error respectively) although equilibria between other aggregates (e.g. octameric and dodecameric) can not be conclusively ruled out (Figure S4) due to broad substrate signal observed.

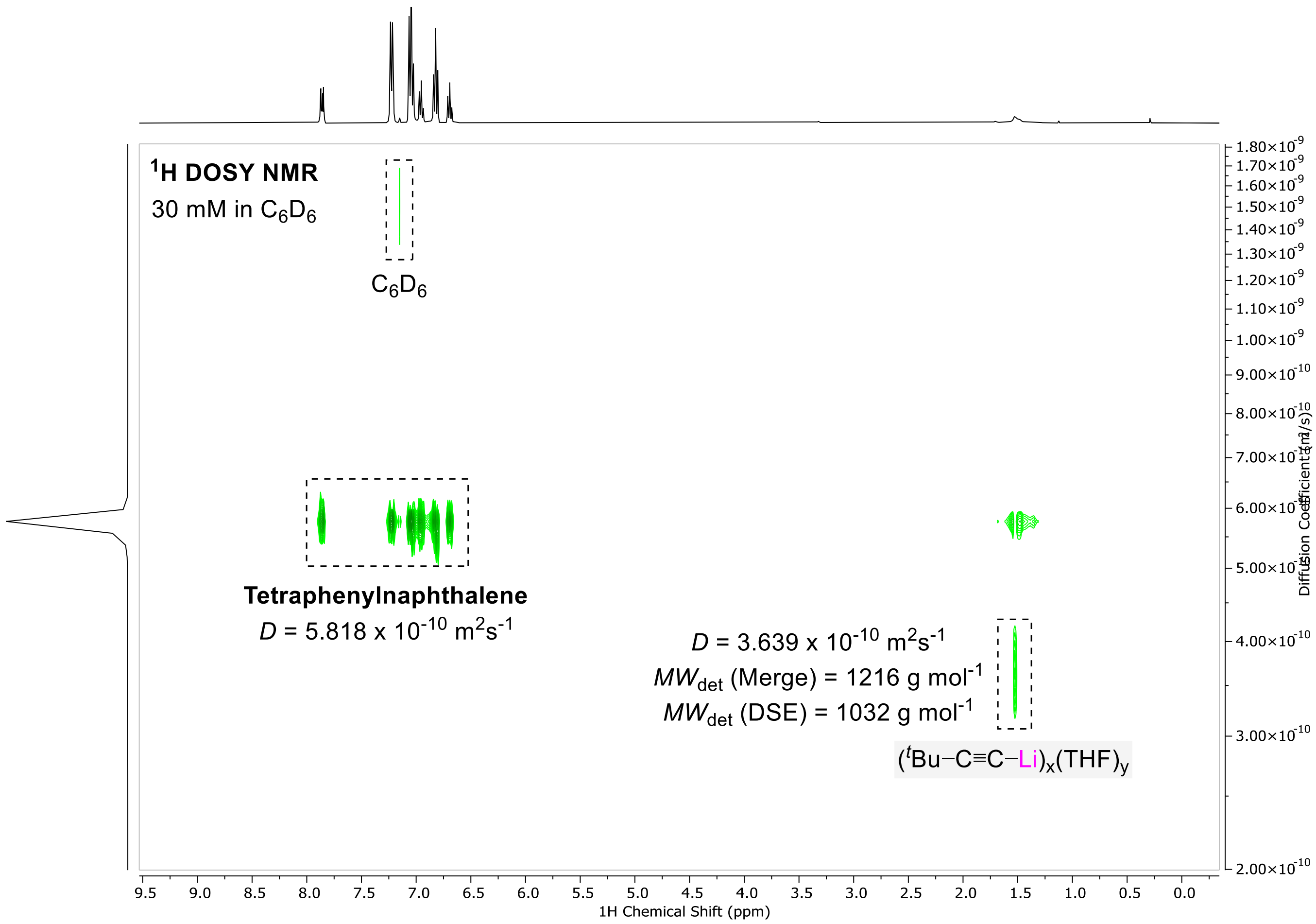

Figure S3: ^{1}H DOSY NMR spectrum of ^{t}Bu–C≡C–Li in C_6D_6 (+ 1 eq. THF-d_8).

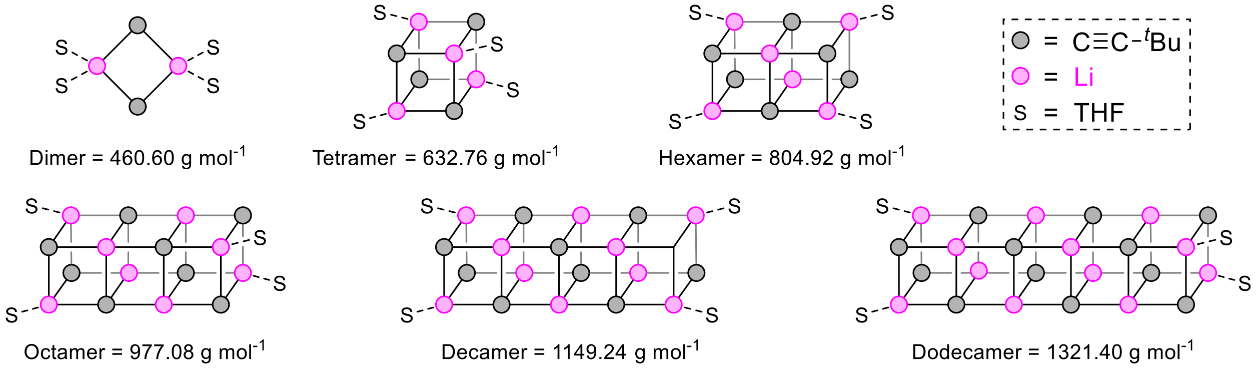

Figure S4: Possible aggregates of ^{t}Bu–C≡C–Li.

^{t}Bu–C≡C–Li in THF-d_8

^{t}Bu–C≡C–Li (1.3 mg, 0.015 mmol) was dissolved in 0.5 mL of THF-d_8 and 1,2,3,4-tetraphenylnaphthalene (6.5 mg, 0.015 mmol) was added as an internal standard. The ^{1}H DOSY NMR spectrum (Figure S5) indicates that a tetrameric aggregate [$Li_4(THF)_4(C{\equiv}C{-}{}^{t}Bu)_4$] is present in THF solution, consistent with literature reports.[8,9] Estimated molecular weight from the measured diffusion coefficient = 587 g mol^{-1} (Merge) or 654 g mol^{-1} (CS = compact spheres); calculated molecular weight for [$Li_4(THF)_4(C{\equiv}C{-}{}^{t}Bu)_4$] = 632.76 g mol^{-1} (+8% or -3% error respectively).

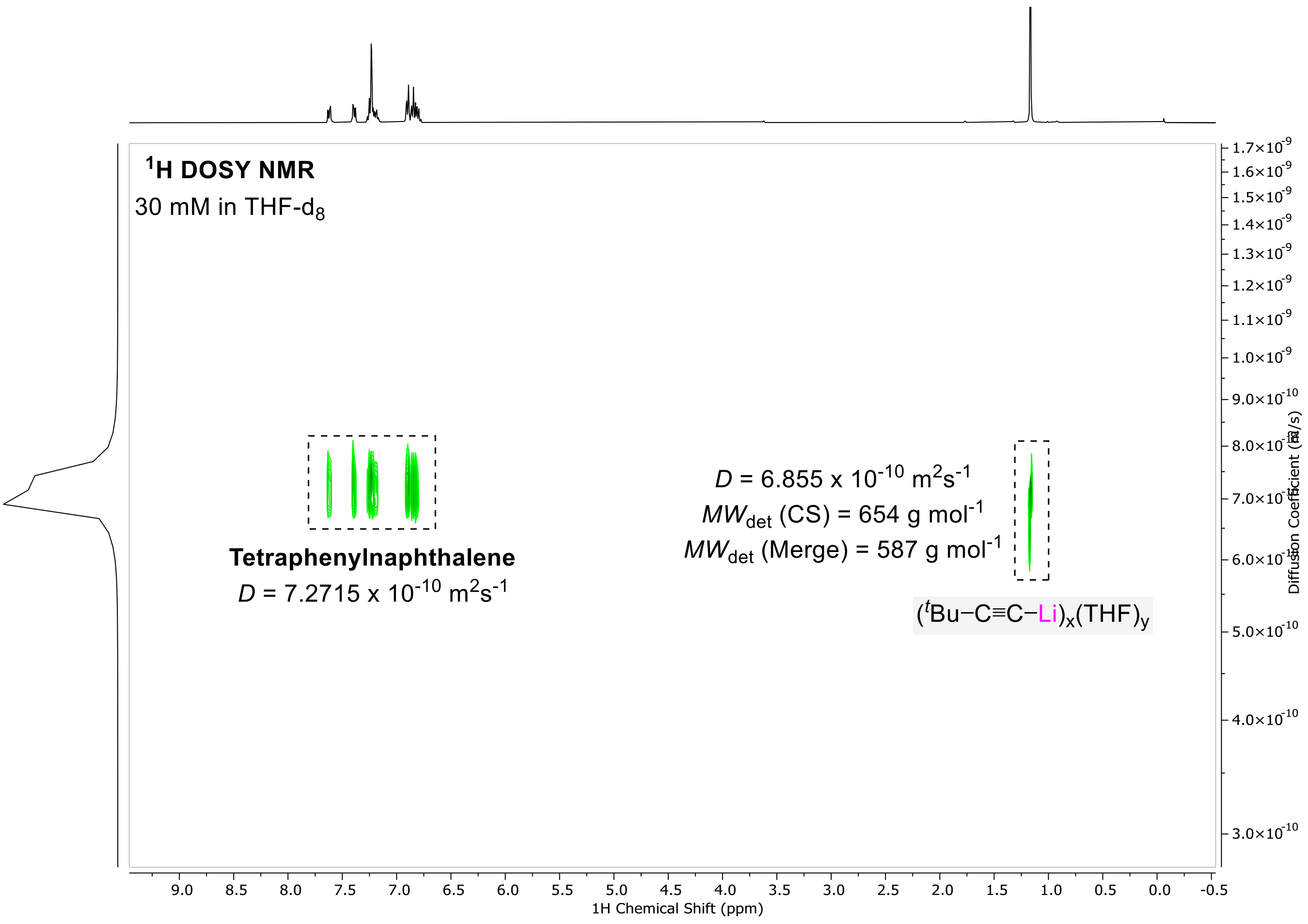

Figure S5: ^{1}H DOSY NMR spectrum of ^{t}Bu–C≡C–Li in THF-d_8.

Me_3Si–C≡C–Li in C_6D_6 (+ 1 eq. THF-d_8)

Me_3Si–C≡C–Li (1.6 mg, 0.015 mmol) was suspended in 0.5 mL of C_6D_6 and 1,2,3,4-tetraphenylnaphthalene (6.5 mg, 0.015 mmol) was added as an internal standard. One equivalent of THF-d_8 (1.2 µL) was added which enabled partial solubility of the lithium acetylide. The ^{1}H DOSY NMR spectrum (Figure S6) suggests that large aggregates exist in solution with an estimated molecular weight between 830 g mol^{-1} (DSE) and 959 g mol^{-1} (Merge). This is consistent with a hexameric aggregate [$Li_6(THF)_4$(C≡C–$SiMe_3)_6$] (Calculated MW = 913.16 g mol^{-1}; +5% or +10% respectively) although other aggregates and equilibria between multiple species (Figure S7) can not be conclusively ruled out due to the broad substrate signal observed.

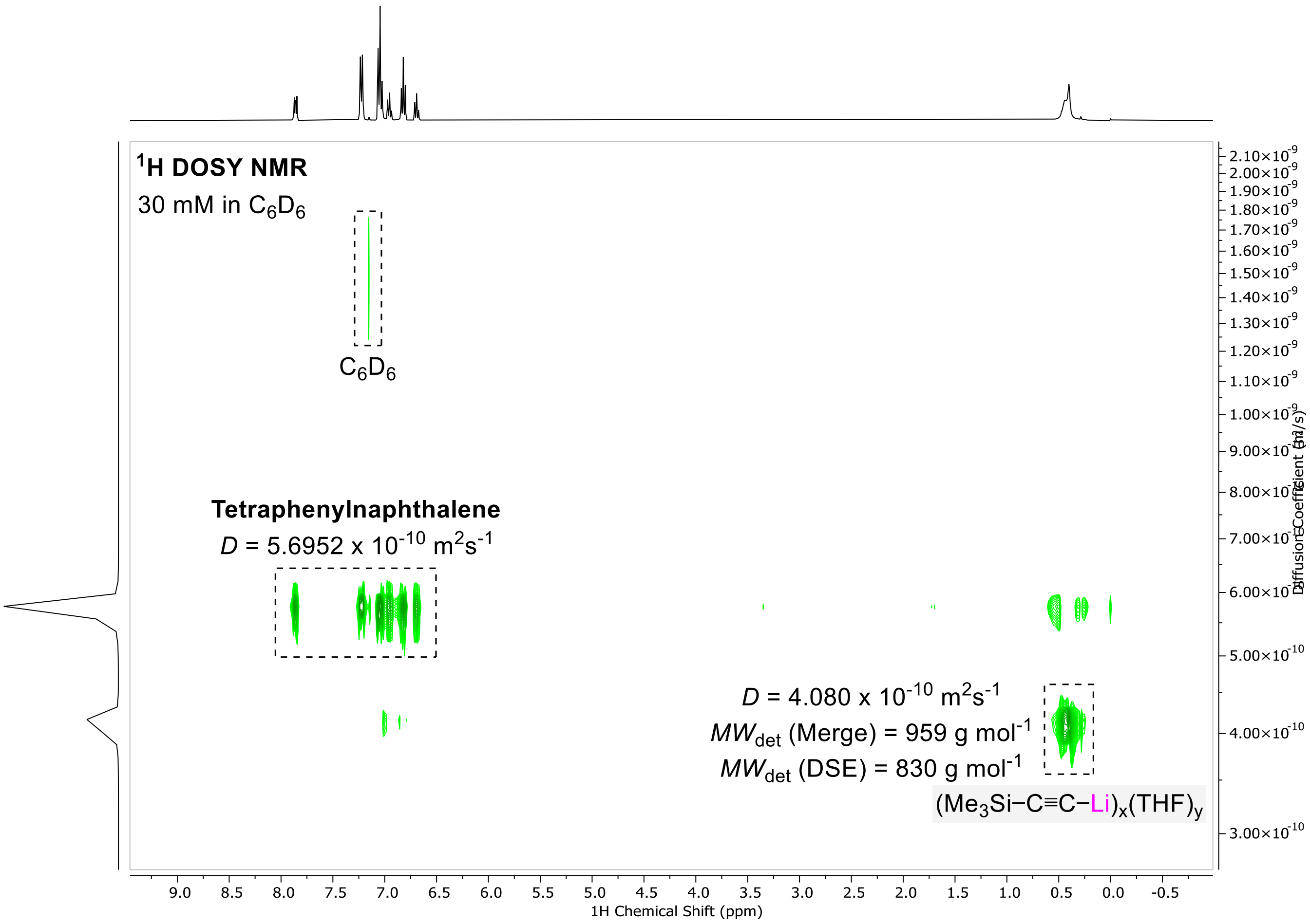

Figure S6: ¹H DOSY NMR spectrum of $Me_3Si–C≡C–Li$ in C_6D_6 (+ 1 eq. THF-d_8).

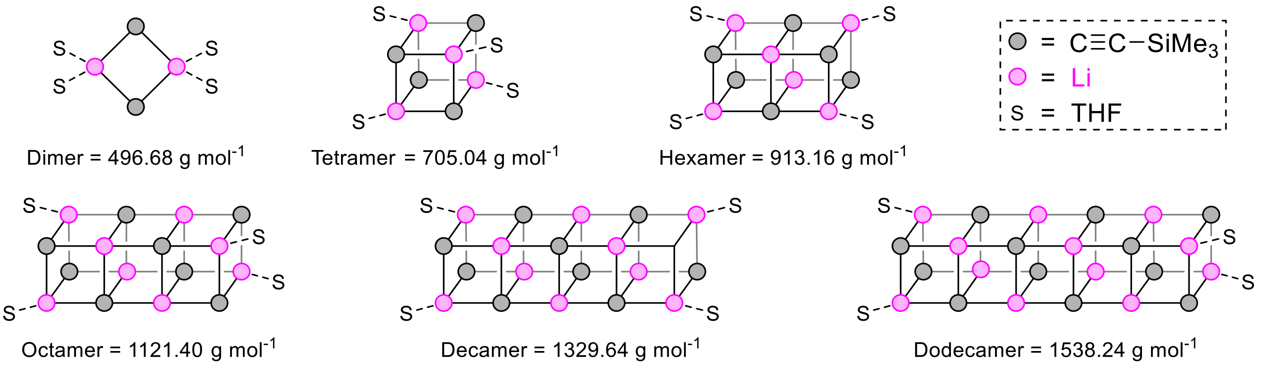

Figure S7: Possible aggregates of $Me_3Si–C≡C–Li$.

$Me_3Si–C≡C–Li$ in THF-d_8

$Me_3Si–C≡C–Li$ (1.6 mg, 0.015 mmol) was dissolved in 0.5 mL of THF-d_8 and 1,2,3,4-tetraphenylnaphthalene (6.5 mg, 0.015 mmol) was added as an internal standard. The ¹H DOSY NMR spectrum (Figure S8) indicates that a dimeric aggregate $[Li_2(THF)_4(C≡C–SiMe_3)_4]$ is present in THF solution, consistent with literature reports.[10] Estimated molecular weight from the measured diffusion coefficient = 502 g mol^{-1} (Merge) or 467 g mol^{-1} (DSE); calculated molecular weight for $[Li_2(THF)_4(C≡C–SiMe_3)_4]$ =496.68 g mol^{-1} (-1% or +6% error respectively).

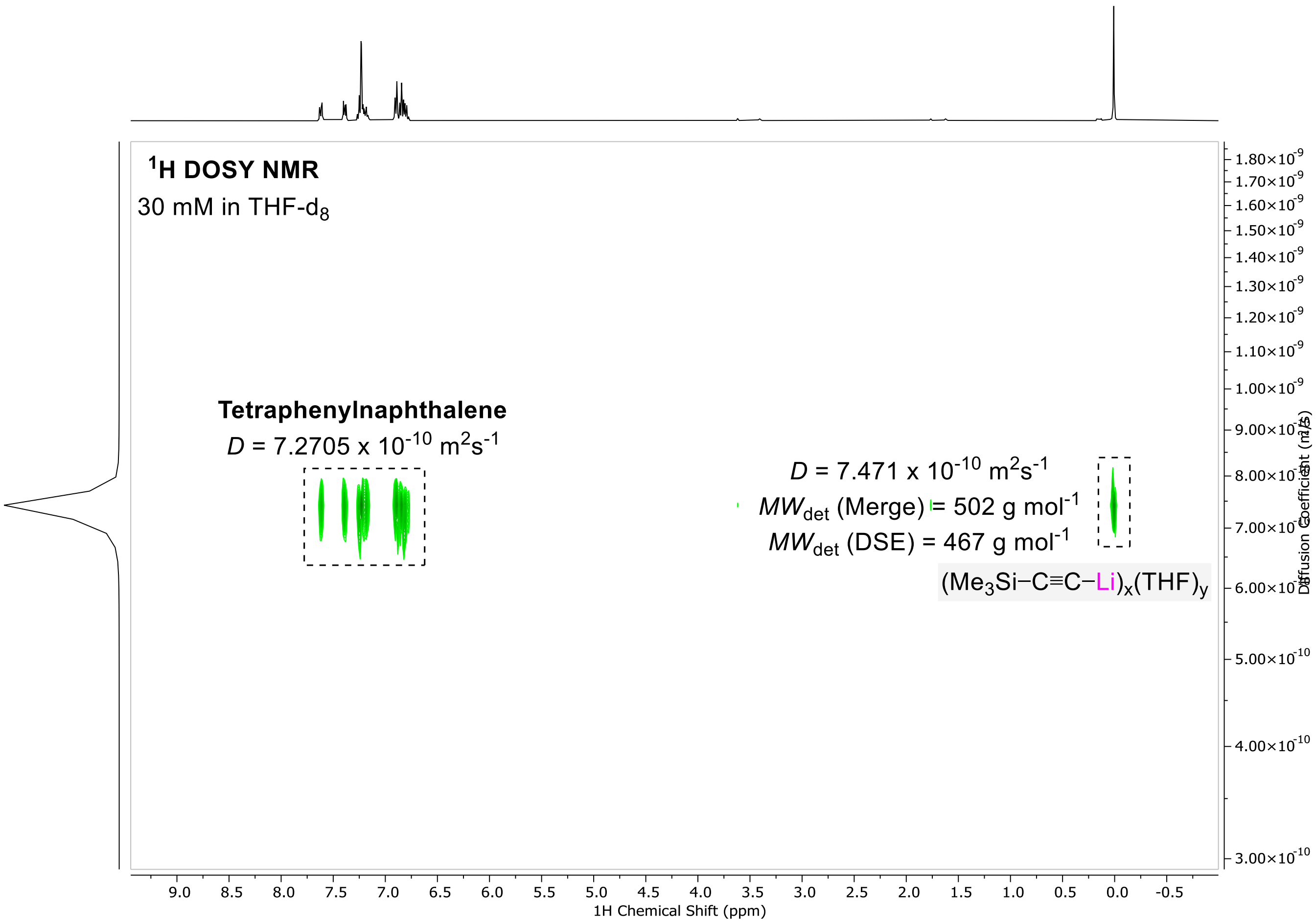

Figure S8: ^{1}H DOSY NMR spectrum of Me_3Si–C≡C–Li in THF-d_8.

[Li_9Ni(C≡C–^{t}Bu)$_9$]$_2$ (2) in Toluene-d_8

17 mg (0.01 mmol) of [Li_9Ni(C≡C–^{t}Bu)$_9$]$_2$ (**2**) was dissolved in 0.5 mL of toluene-d_8 and analysed by ^{1}H DOSY NMR spectroscopy. The ^{1}H DOSY NMR spectrum indicates that only one major species exists in solution with no evidence of lithium acetylide dissociation from the lithium nickelate cluster (Figure S9). The estimated molecular weight determined from the measured diffusion coefficient is 862 g mol^{-1} (CS); this is approximately half the molecular weight of [Li_9Ni(C≡C–^{t}Bu)$_9$]$_2$ (1702.79 g mol^{-1}) suggesting that the cluster dissociates to “Li_9Ni(C≡C–^{t}Bu)$_9$” in solution (MW = 851.39 g mol^{-1}; -1% difference).

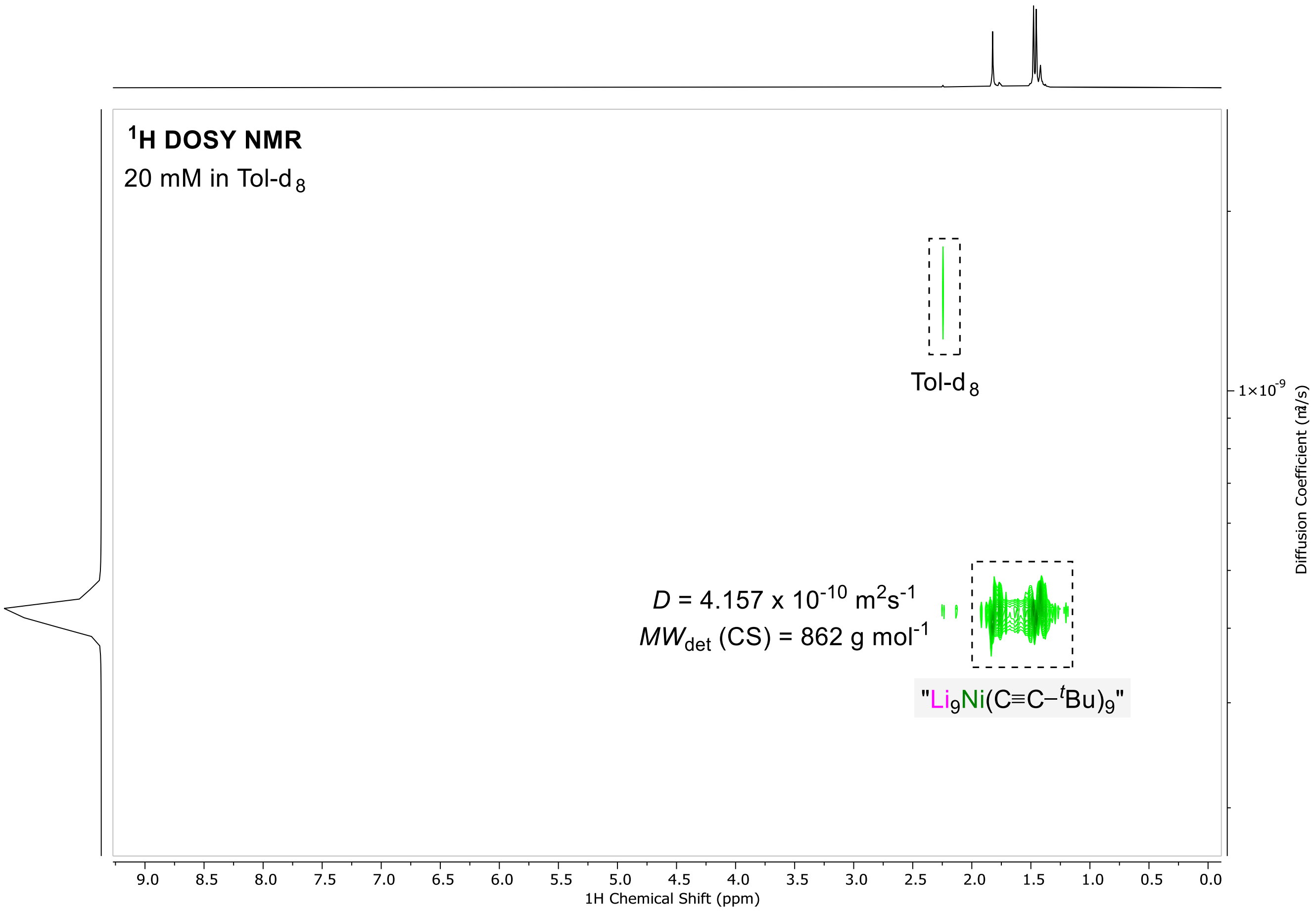

Figure S9: ^{1}H DOSY NMR spectrum of $[Li_9Ni(C{\equiv}C–{}^tBu)_9]_2$ (**2**) in toluene-d_8.

$[Li_9Ni(C{\equiv}C–{}^tBu)_9]_2$ (2) in THF-d_8

17 mg (0.01 mmol) of $[Li_9Ni(C{\equiv}C–{}^tBu)_9]_2$ (**2**) was dissolved in 0.5 mL of THF-d_8 and analysed by ^{1}H, ^{7}Li and ^{1}H DOSY NMR spectroscopy. The ^{1}H and ^{7}Li NMR spectra clearly show that free ^{t}Bu–C≡C–Li is present in solution (Figures S10–11) consistent with dissociation of the lithium nickelate cluster in donor solvents. The ^{1}H DOSY NMR spectrum also supports that free lithium acetylide dissociates from **2** which gives two major species that do not co-diffuse (Figure S12). The estimated molecular weight determined from the measured diffusion coefficient of the proposed lithium nickelate component is between 542 g mol^{-1} (DSE) and 588 g mol^{-1} (Merge). This is in good agreement with a tri-lithium nickelate unit "$Li_3(THF)_3Ni(C{\equiv}C–{}^tBu)_3$" which is a core building block of cluster **2** (MW = 539.25 g mol^{-1}; -1% or +8% difference respectively). For the free lithium acetylide, whilst this exists as a dimeric aggregate in THF-d_8 solution (see Figure S5), exchange processes between the free organolithium and lithium nickelate (or overlap of signals) appear to give lower estimated molecular weights than expected [$D = 6.208 \times 10^{-10}$ m^{2}s^{-1}; MW = 398 g mol^{-1} (Merge) or 375 g mol^{-1} (DSE)].

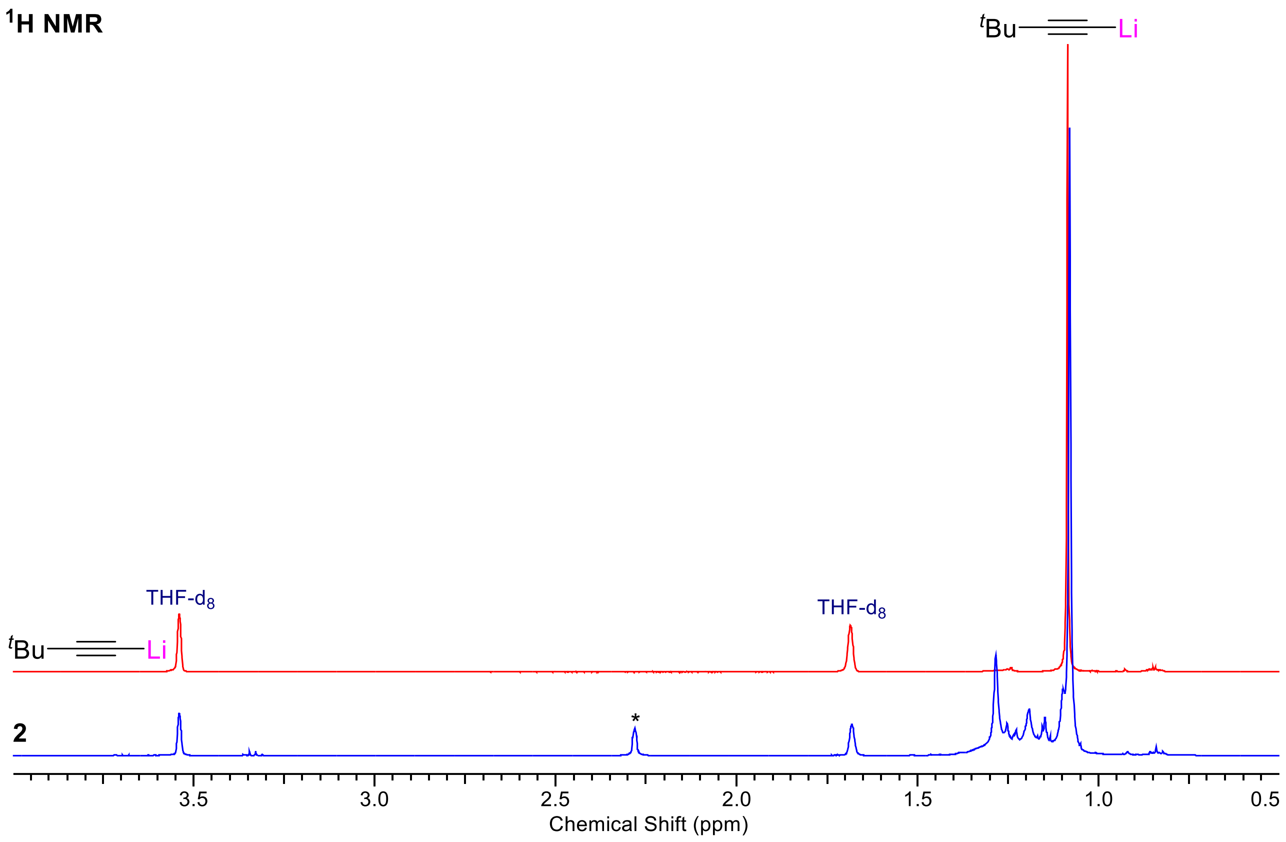

Figure S10: Stacked ^{1}H NMR spectra comparing free ^{t}Bu–C≡C–Li (red trace) with $[Li_9Ni(C{\equiv}C{-}^tBu)_9]_2$ (**2**, blue trace) in THF-d_8.

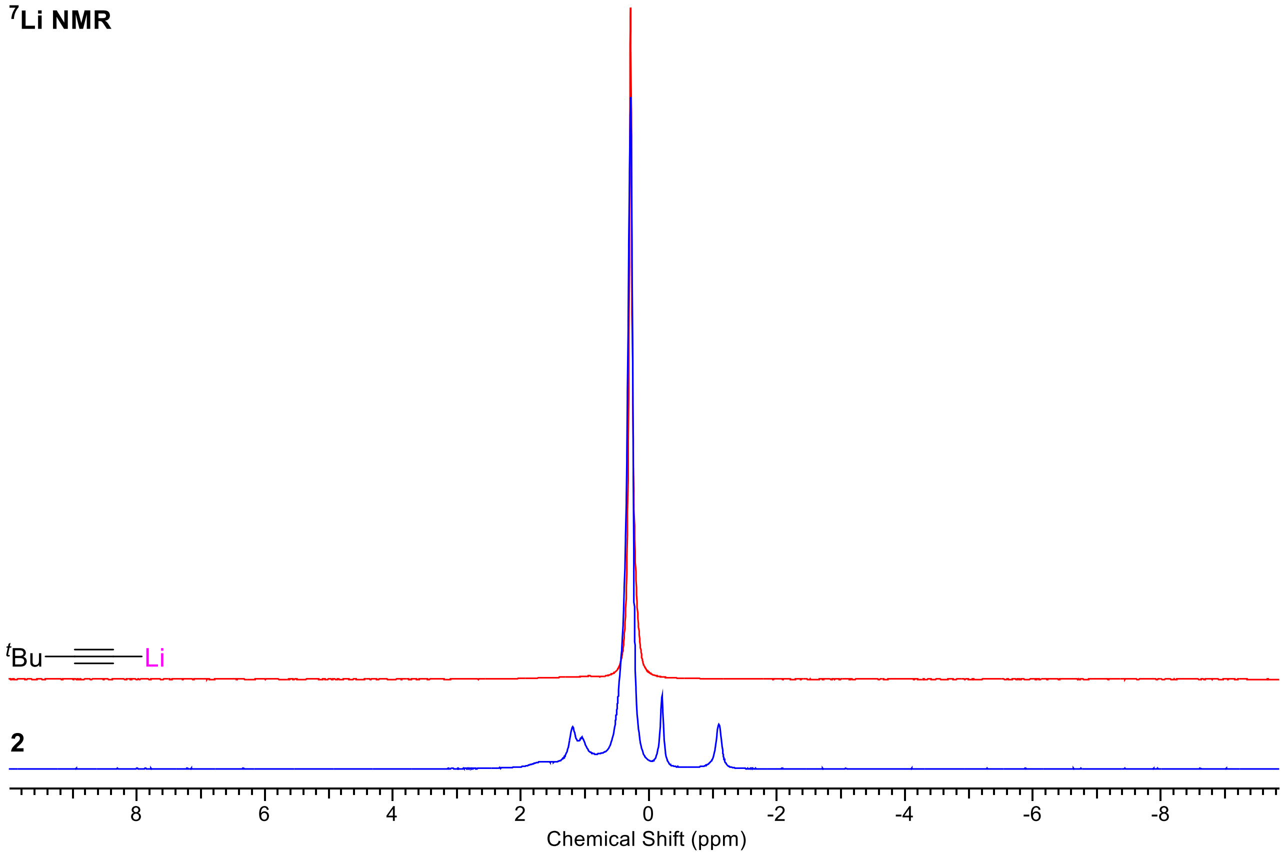

Figure S11: Stacked ^{7}Li NMR spectra comparing free ^{t}Bu–C≡C–Li (red trace) with $[Li_9Ni(C{\equiv}C{-}^tBu)_9]_2$ (**2**, blue trace) in THF-d_8.

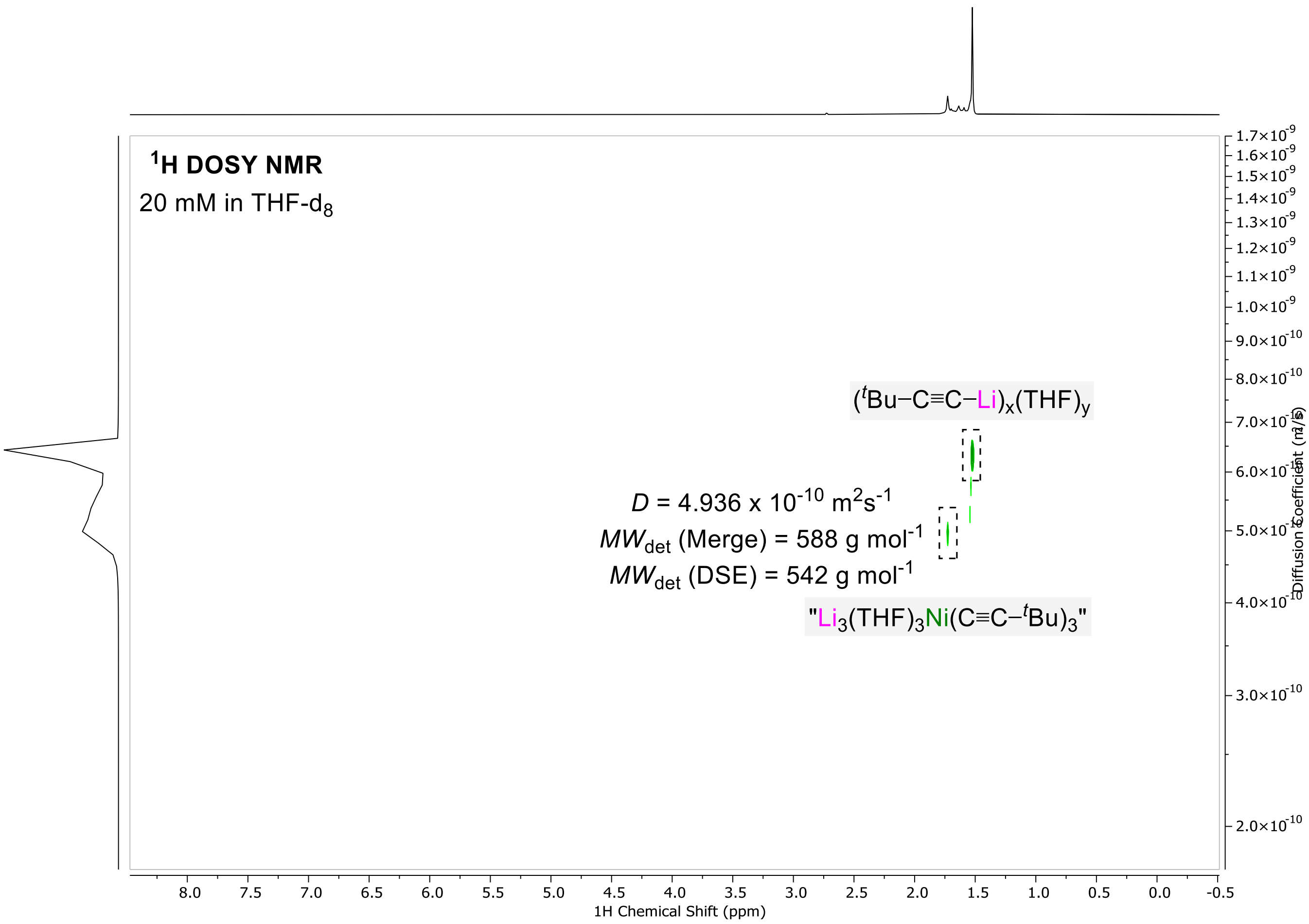

Figure S12: ^{1}H DOSY NMR spectrum of $[Li_9Ni(C{\equiv}C–^{t}Bu)_9]_2$ (**2**) in THF-d_8.

$Li_{10}(Et_2O)_3Ni(C{\equiv}C–SiMe_3)_{10}$ (3) in Toluene-d_8

15 mg (0.01 mmol) of $Li_{10}(Et_2O)_3Ni(C{\equiv}C–SiMe_3)_{10}$ (**3**) was dissolved in 0.5 mL of toluene-d_8 and analysed by ^{1}H DOSY NMR spectroscopy. The ^{1}H DOSY NMR spectrum indicates that only one major species exists in solution with no evidence of lithium acetylide dissociation from the lithium nickelate cluster (Figure S13). The estimated molecular weight determined from the measured diffusion coefficient is 1262 g mol^{-1} (DSE) or 1397 g mol^{-1} (Merge). This is consistent with partial Et_2O dissociation from the cluster to give $Li_{10}(Et_2O)_1Ni(C{\equiv}C–SiMe_3)_{10}$ in solution (MW = 1336.72 g mol^{-1}; +6 or -4% difference).

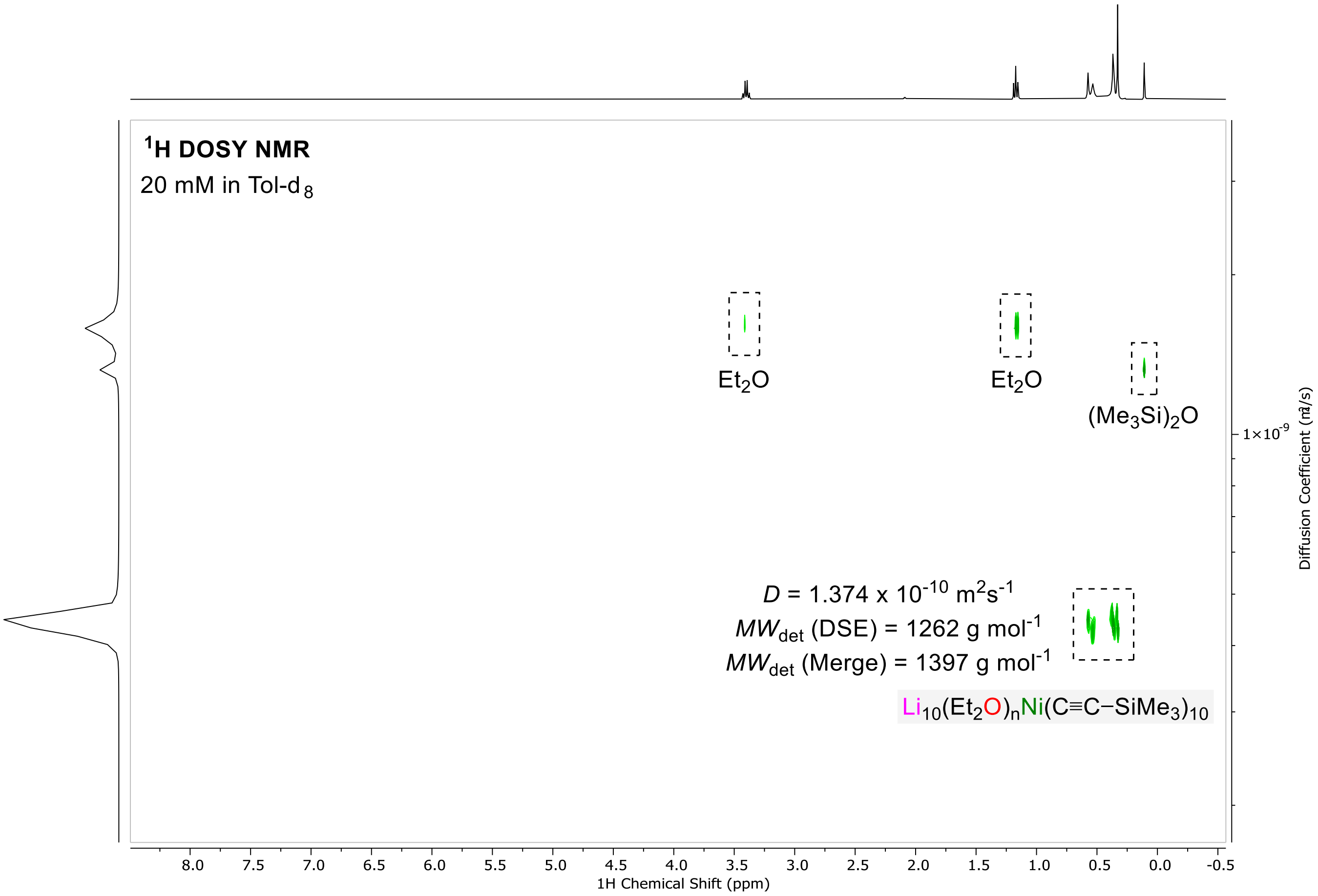

Figure S13: ^{1}H DOSY NMR spectrum of $Li_{10}(Et_2O)_3Ni(C{\equiv}C–SiMe_3)_{10}$ (**3**) in toluene-d_8.

$Li_{10}(Et_2O)_3Ni(C{\equiv}C–SiMe_3)_{10}$ (3) in THF-d_8

6 mg (0.01 mmol) of $Li_{10}(Et_2O)_3Ni(C{\equiv}C–SiMe_3)_{10}$ (**3**) was dissolved in 0.5 mL of THF-d_8 and analysed by ^{1}H DOSY NMR spectroscopy. The ^{1}H DOSY NMR spectrum indicates that the lithium nickelate dissociates into two major components which are proposed to be $Li_4(THF)_8Ni(C{\equiv}C–SiMe_3)_4$ and $(Me_3Si–C{\equiv}C–Li)_x(THF)_y$ (Figure S14). The estimated molecular weight of $Li_4(THF)_8Ni(C{\equiv}C–SiMe_3)_4$ determined from the measured diffusion coefficient is 952 g mol^{-1} (DSE) or 1062 g mol^{-1} (Merge) which is close to the calculated molecular weight of 1052.16 g mol^{-1} (+11 or -1% error respectively). Whilst the free lithium acetylide exists as a dimeric aggregate in THF-d_8 solution (see Figure S8), exchange processes between the free organolithium and lithium nickelate (or overlap of signals) appear to give higher estimated molecular weights than expected [D = 6.577 x 10^{-10} m^{2}s^{-1}; MW = 741 g mol^{-1} (DSE)].

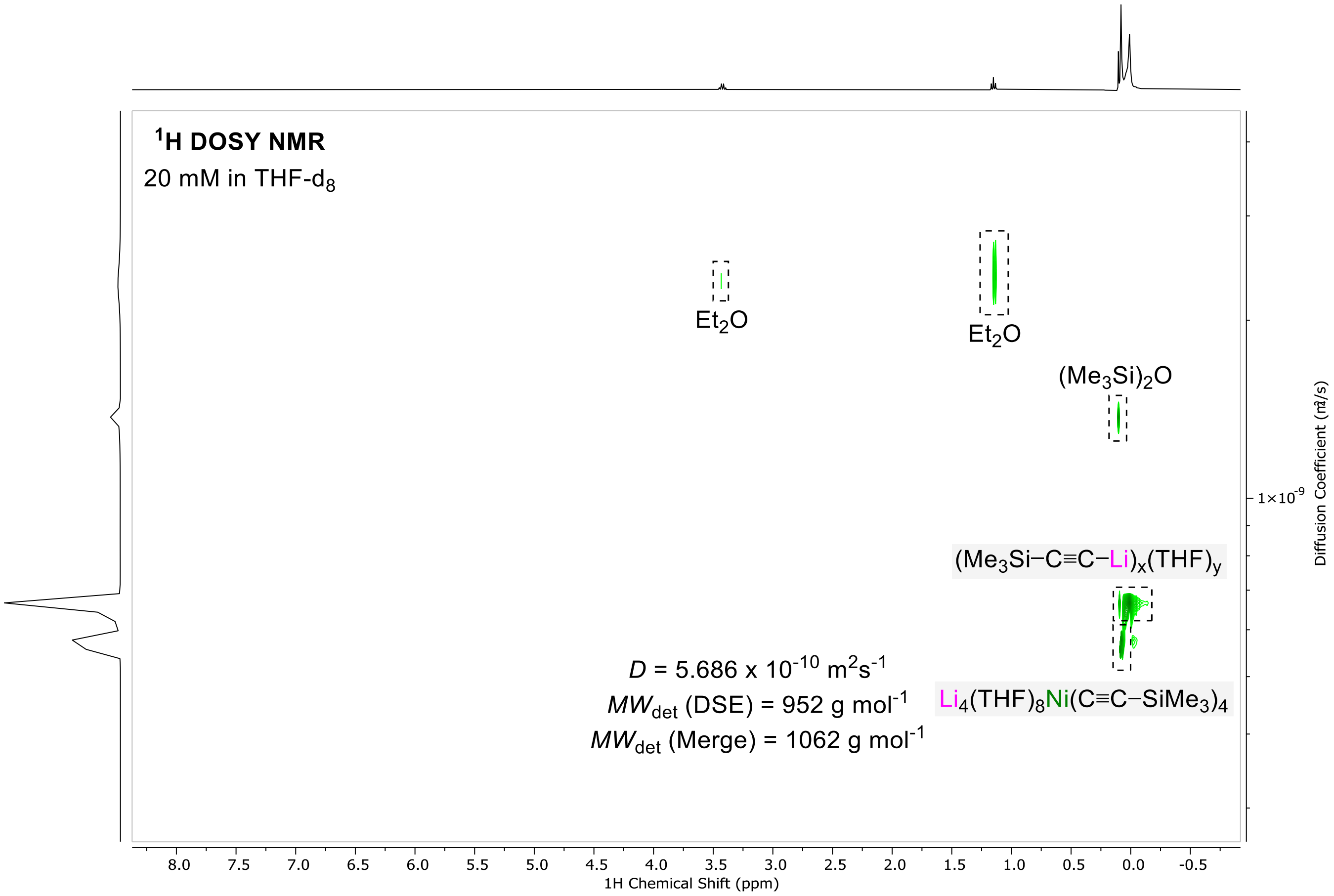

Figure S14: ^{1}H DOSY NMR spectrum of $Li_{10}(Et_2O)_3Ni(C{\equiv}C–SiMe_3)_{10}$ (**3**) in THF-d$_8$.

The dissociation of $Li_{10}(Et_2O)_3Ni(C{\equiv}C–SiMe_3)_{10}$ into $Li_4(THF)_8Ni(C{\equiv}C–SiMe_3)_4$ and $(Me_3Si–C{\equiv}C–Li)_x(THF)_y$ is further supported by inspecting the 1D ^{1}H NMR spectrum which shows three distinct Me_3Si environments (Figure S15); a sharp singlet at δ 0.02 with an integral of 18H which corresponds to one equivalent of co-crystallised $(Me_3Si)_2O$; a broader singlet at δ 0.00 with an integral of 36H consistent with $Li_4(THF)_nNi(C{\equiv}C–SiMe_3)_4$; and a very broad signal at δ -0.07 (with an overlapping shoulder) with a combined integral of 54H which is proposed to be free lithium acetylide, $(Me_3Si–C{\equiv}C–Li)_x(THF)_y$, which dissociates from the lithium nickelate cluster in THF solution. The ^{7}Li NMR spectrum (Figure S16) also supports this hypothesis and displays two sharp signals at δ 0.20 and δ 0.06 with relative ratios of 6:4 for $(Me_3Si–C{\equiv}C–Li)_x(THF)_y$ and $Li_4(THF)_nNi(C{\equiv}C–SiMe_3)_4$ respectively.

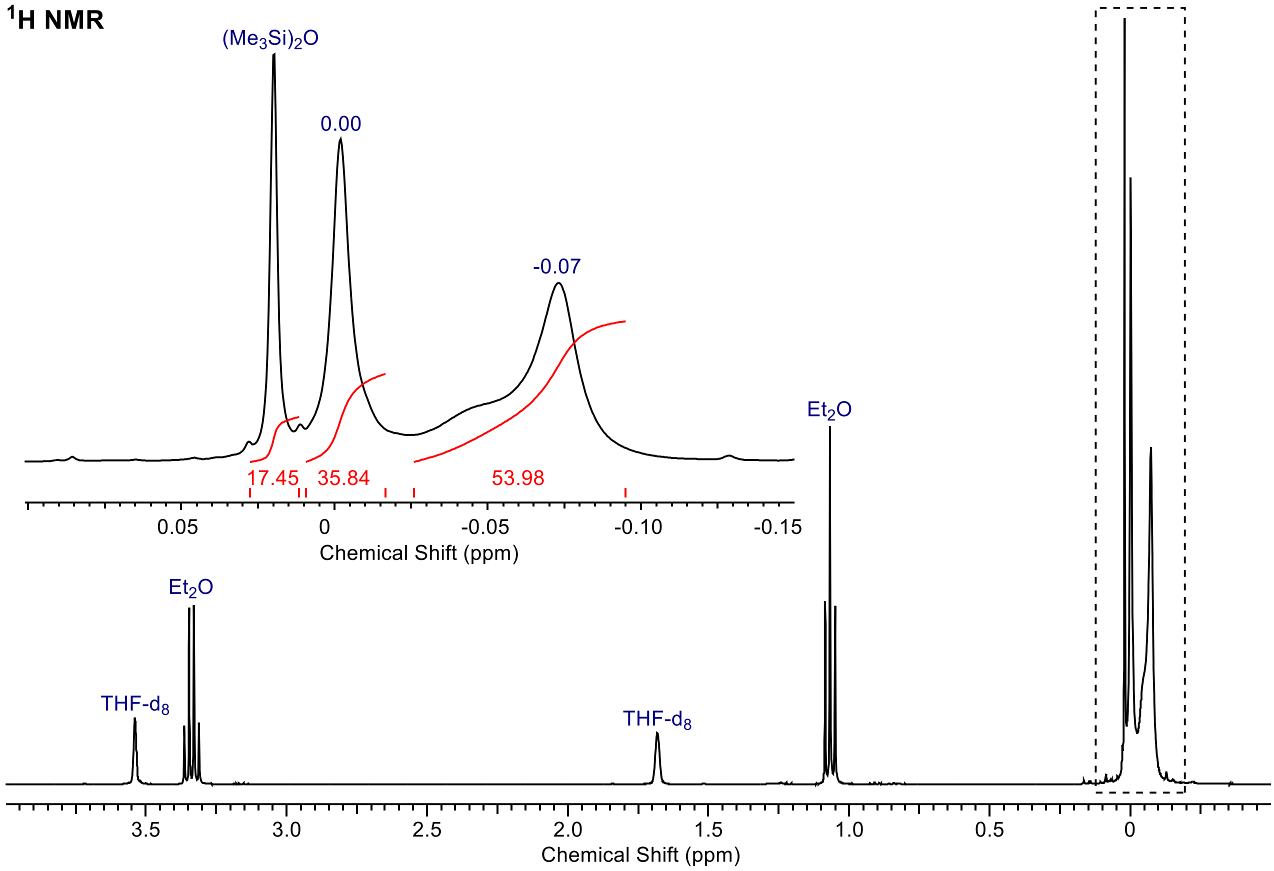

Figure S15: ^{1}H NMR spectrum of $Li_{10}(Et_2O)_3Ni(C{\equiv}C{-}SiMe_3)_{10}$ (**3**) in THF-d_8.

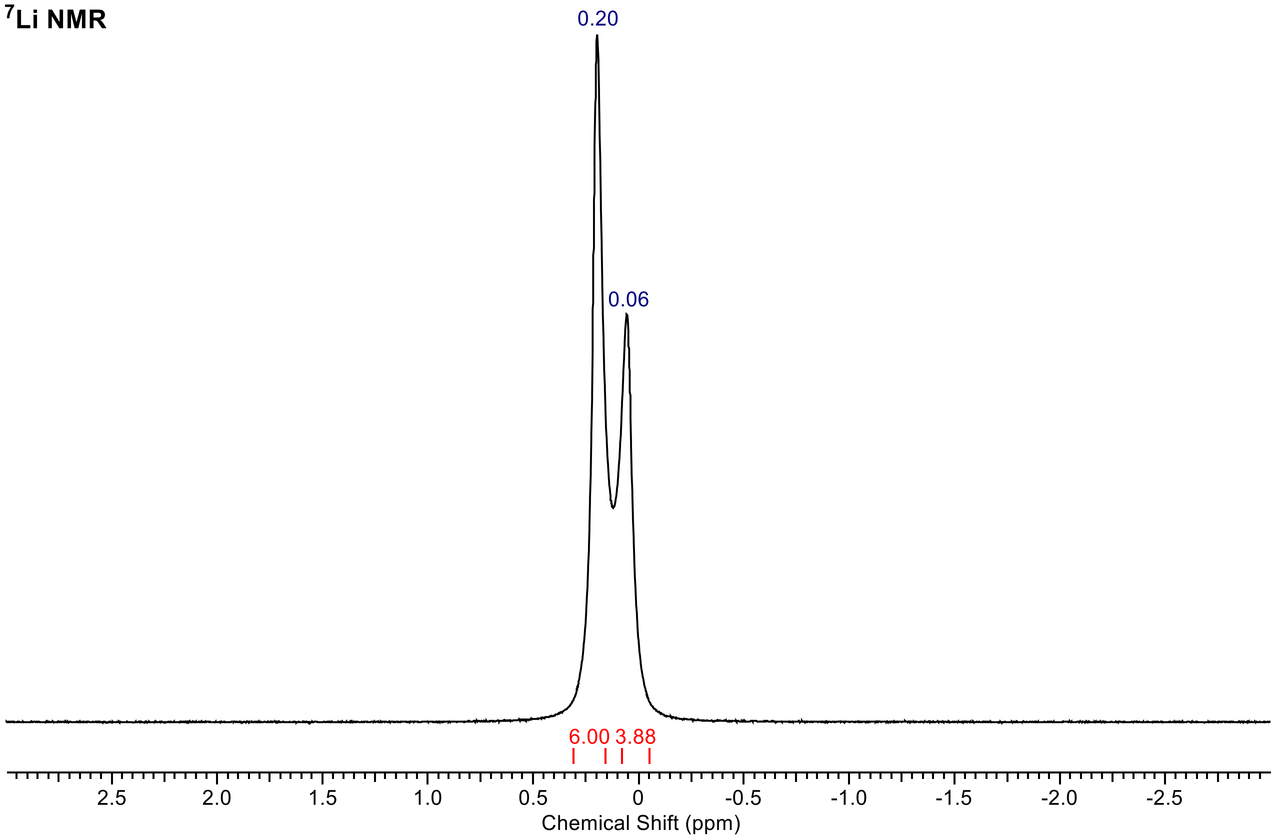

Figure S16: ^{7}Li NMR spectrum of $Li_{10}(Et_2O)_3Ni(C{\equiv}C{-}SiMe_3)_{10}$ (**3**) in THF-d_8.

$Li_{10}(Et_2O)_3Ni(C{\equiv}C{-}^tBu)_{10}$ (4) in Toluene-d_8

12 mg (0.01 mmol) of $Li_{10}(Et_2O)_3Ni(C{\equiv}C{-}^tBu)_{10}$ (**4**) was dissolved in 0.5 mL of toluene-d_8 and analysed by ^1H DOSY NMR spectroscopy. The ^1H DOSY NMR spectrum indicates that only one major species exists in solution with no evidence of lithium acetylide dissociation from the lithium nickelate cluster (Figure S17). The estimated molecular weight determined from the measured diffusion coefficient is 845 g mol^{-1} (Merge) or 1090 g mol^{-1} (CS). This is consistent with Et_2O dissociation from the cluster to give either $Li_{10}Ni(C{\equiv}C{-}^tBu)_{10}$ (MW = 939.47 g mol^{-1}; +11% or -14% difference) or $Li_{10}(Et_2O)_1Ni(C{\equiv}C{-}^tBu)_{10}$ (MW = 1013.60 g mol^{-1}; +20% or -7% difference).

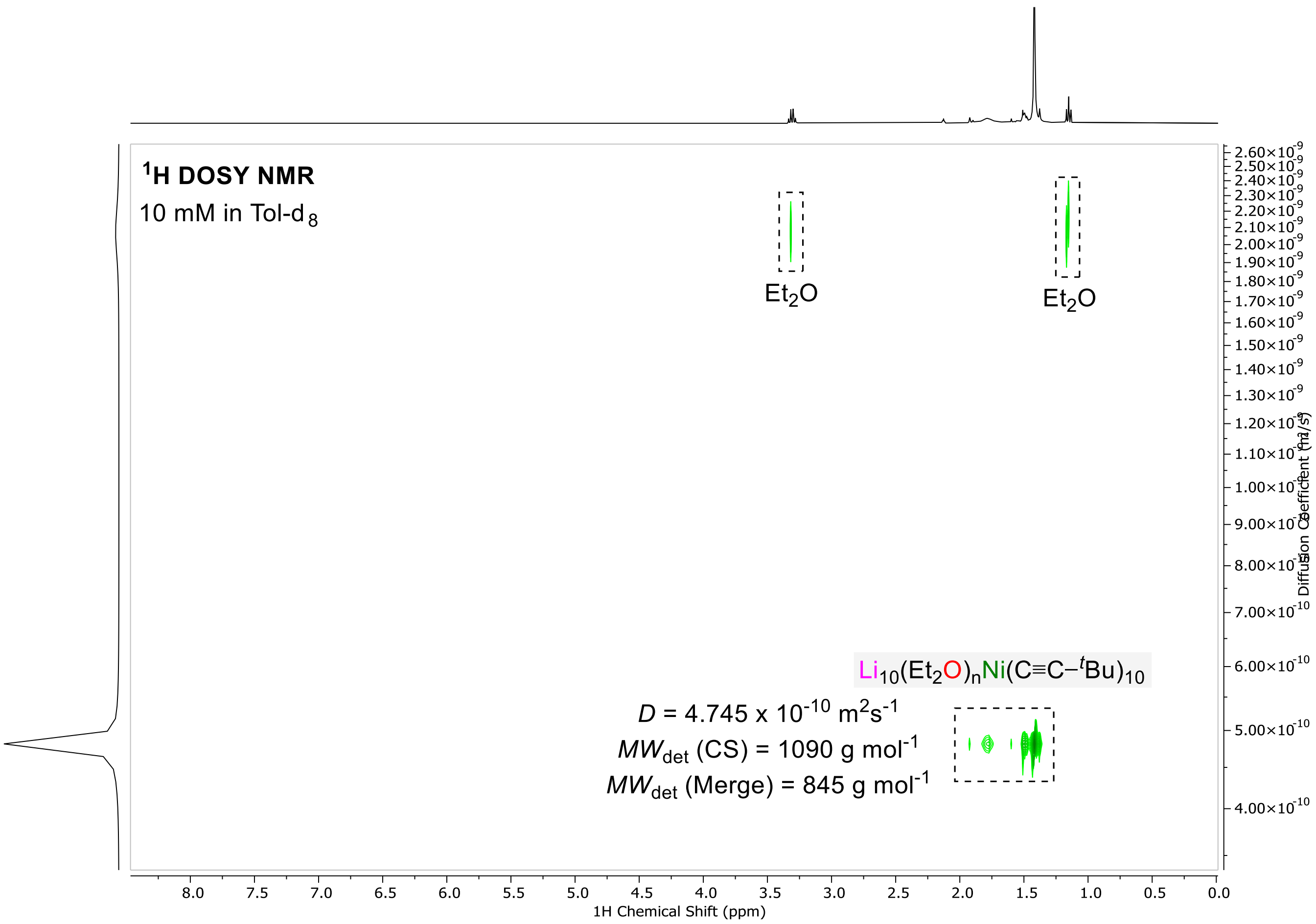

Figure S17: ^1H DOSY NMR spectrum of $Li_{10}(Et_2O)_3Ni(C{\equiv}C{-}^tBu)_{10}$ (**4**) in toluene-d_8.

$[Li_{11}(Et_2O)Ni_2(C{\equiv}C{-}^tBu)_{11}]_2$ (5) in Toluene-d_8

6 mg (0.0025 mmol) of $[Li_{11}(Et_2O)Ni_2(C{\equiv}C{-}^tBu)_{11}]_2$ (**5**) was dissolved in 0.5 mL of toluene-d_8 and analysed by ^1H DOSY NMR spectroscopy. The ^1H DOSY NMR spectrum indicates that only one major species exists in solution with no evidence of lithium acetylide dissociation from the lithium nickelate cluster (Figure S18). The estimated molecular weight determined from the measured diffusion coefficient is 2208 g mol^{-1} (CS = compact spheres); this is in good agreement with the molecular weight of $[Li_{11}(Et_2O)Ni_2(C{\equiv}C{-}^tBu)_{11}]_2$ (MW = 2320.74 g mol^{-1}; +5% difference). Alternatively, if modelled as an expanded disc (ED), the estimated molecular weight determined from the measured diffusion coefficient is 962 g mol^{-1}; this provides a reasonable agreement with "$Li_{11}Ni_2(C{\equiv}C{-}^tBu)_{11}$" (MW = 1086.24 g mol^{-1}; +13% difference).

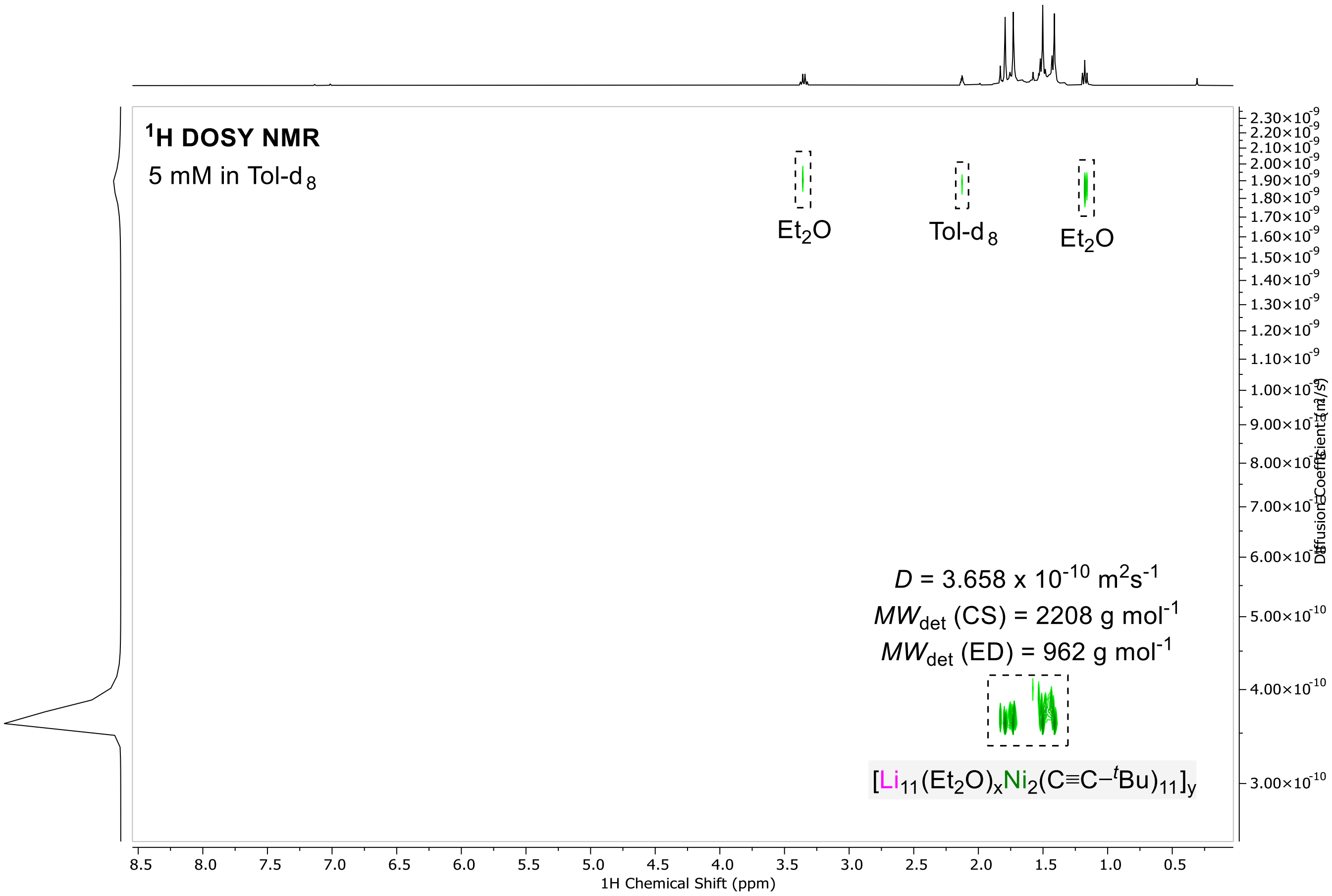

Figure S18: ^{1}H DOSY NMR spectrum of [$Li_{11}(Et_2O)Ni_2(C{\equiv}C{-}^{t}Bu)_{11}]_2$ (**5**) in toluene-d_8.

[$Li_{10}(Et_2O)_2Ni(C{\equiv}C{-}^{i}Pr)_8(C{\equiv}C{-}Me_2O)]_2$ (6) in Toluene-d_8

9 mg (0.005 mmol) of [$Li_{10}(Et_2O)_2Ni(C{\equiv}C{-}^{i}Pr)_8(C{\equiv}C{-}Me_2O)]_2$ (**6**) was dissolved in 0.5 mL of toluene-d_8 and analysed by ^{1}H DOSY NMR spectroscopy. The ^{1}H DOSY NMR spectrum indicates that only one major species exists in solution with no evidence of lithium acetylide dissociation from the lithium nickelate cluster (Figure S19). The estimated molecular weight determined from the measured diffusion coefficient is 1718 g mol^{-1} (CS = compact spheres); this is in good agreement with the molecular weight of [$Li_{10}(Et_2O)_2Ni(C{\equiv}C{-}^{i}Pr)_8(C{\equiv}C{-}Me_2O)]_2$ (MW = 1790.66 g mol^{-1}; +4% difference).

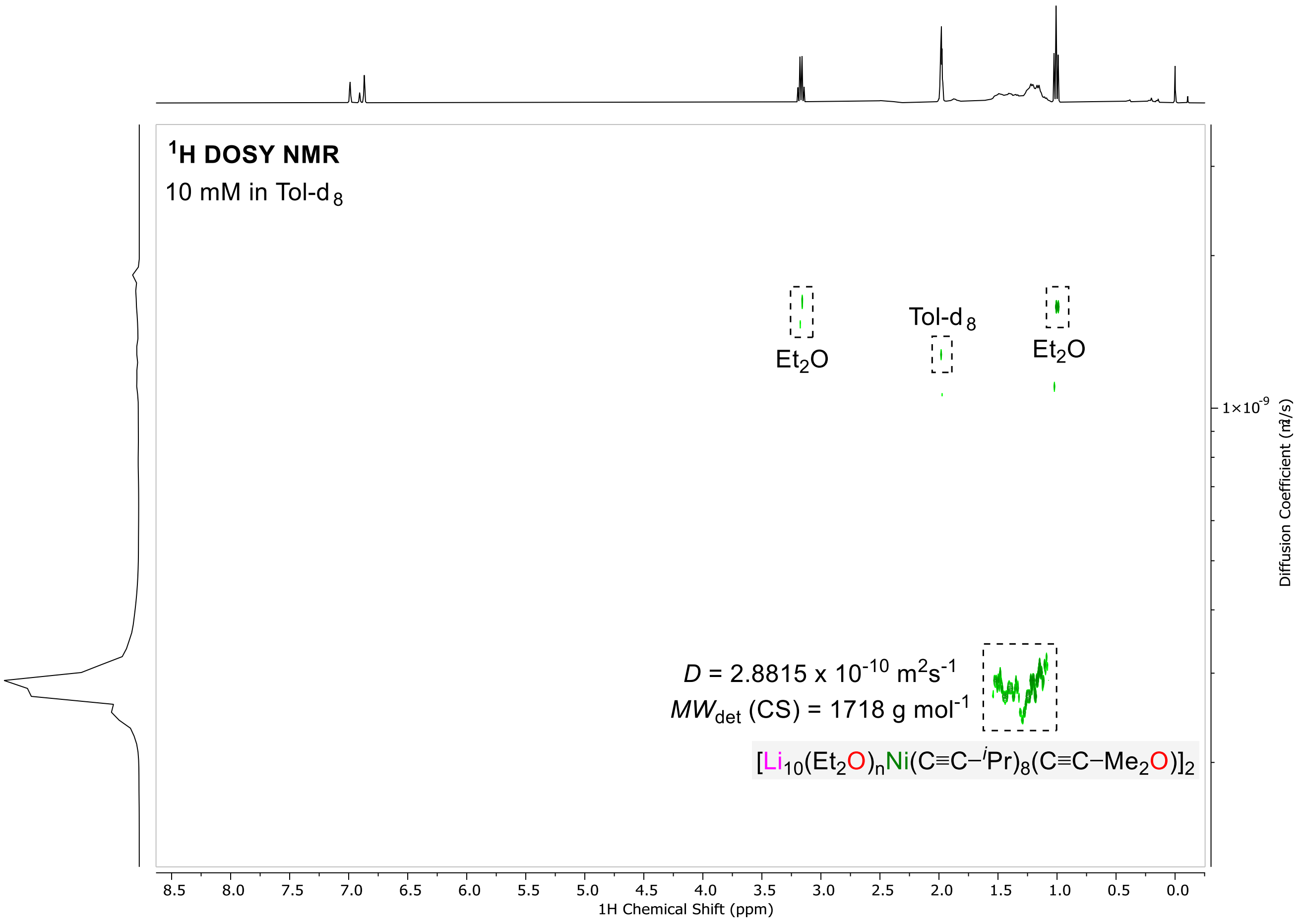

Figure S19: ^{1}H DOSY NMR spectrum of $[Li_{10}(Et_2O)_2Ni(C{\equiv}C{-}^{i}Pr)_8(C{\equiv}C{-}Me_2O)]_2$ (**6**) in toluene-d_8.

Variable Temperature NMR Spectroscopy

The lithium nickelate clusters $[Li_9Ni(C{\equiv}C{-}^{t}Bu)_9]_2$ (**2**) and $Li_{10}(Et_2O)_3Ni(C{\equiv}C{-}SiMe_3)_{10}$ (**3**) were further analysed by variable temperature ^{1}H and ^{7}Li NMR spectroscopy (Figure S20–23). Whilst the broad signals observed at room temperature split into multiple sharp signals upon cooling -80 °C, it was not possible to confidently assign any of these signals to the distinct chemical environments observed in the solid-state structures.

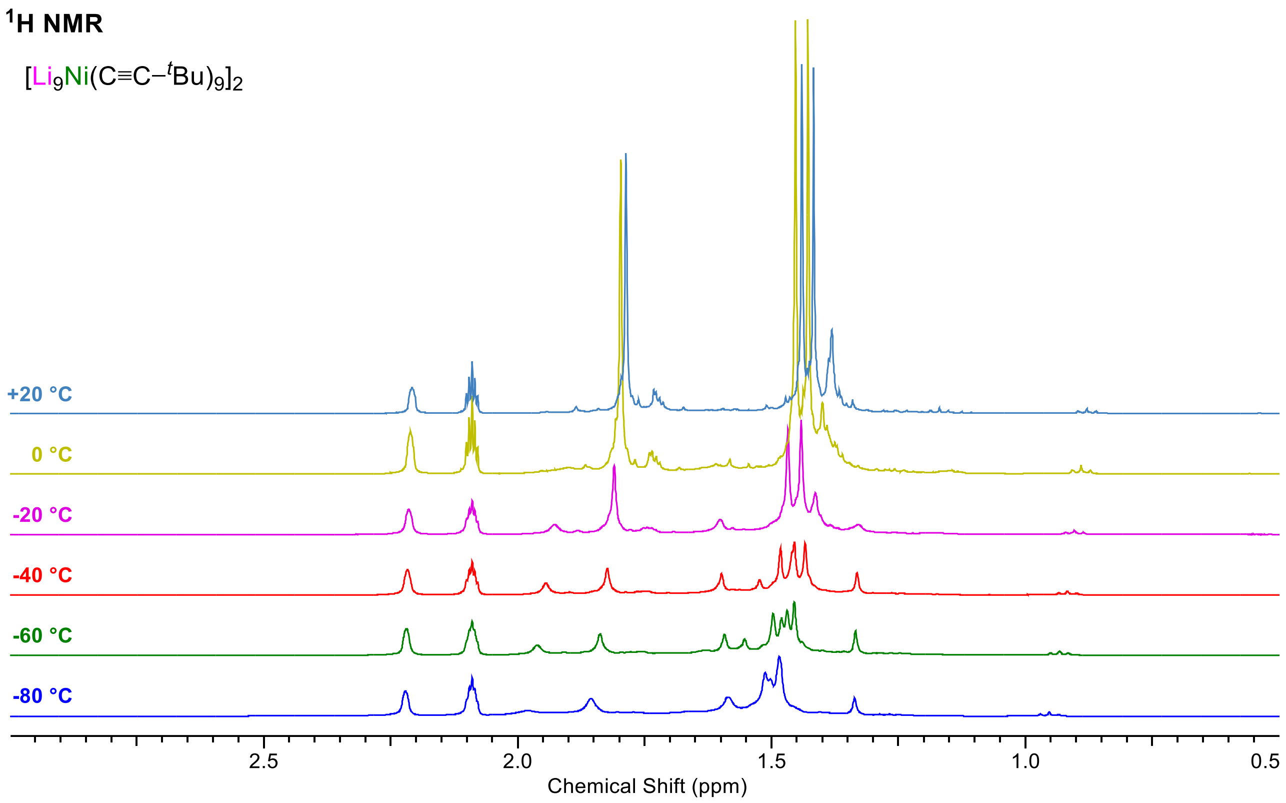

Figure S20: Stacked ^{1}H NMR spectra of [$Li_9Ni(C≡C–^tBu)_9]_2$ (**2**) in toluene-d$_8$ at variable temperatures.

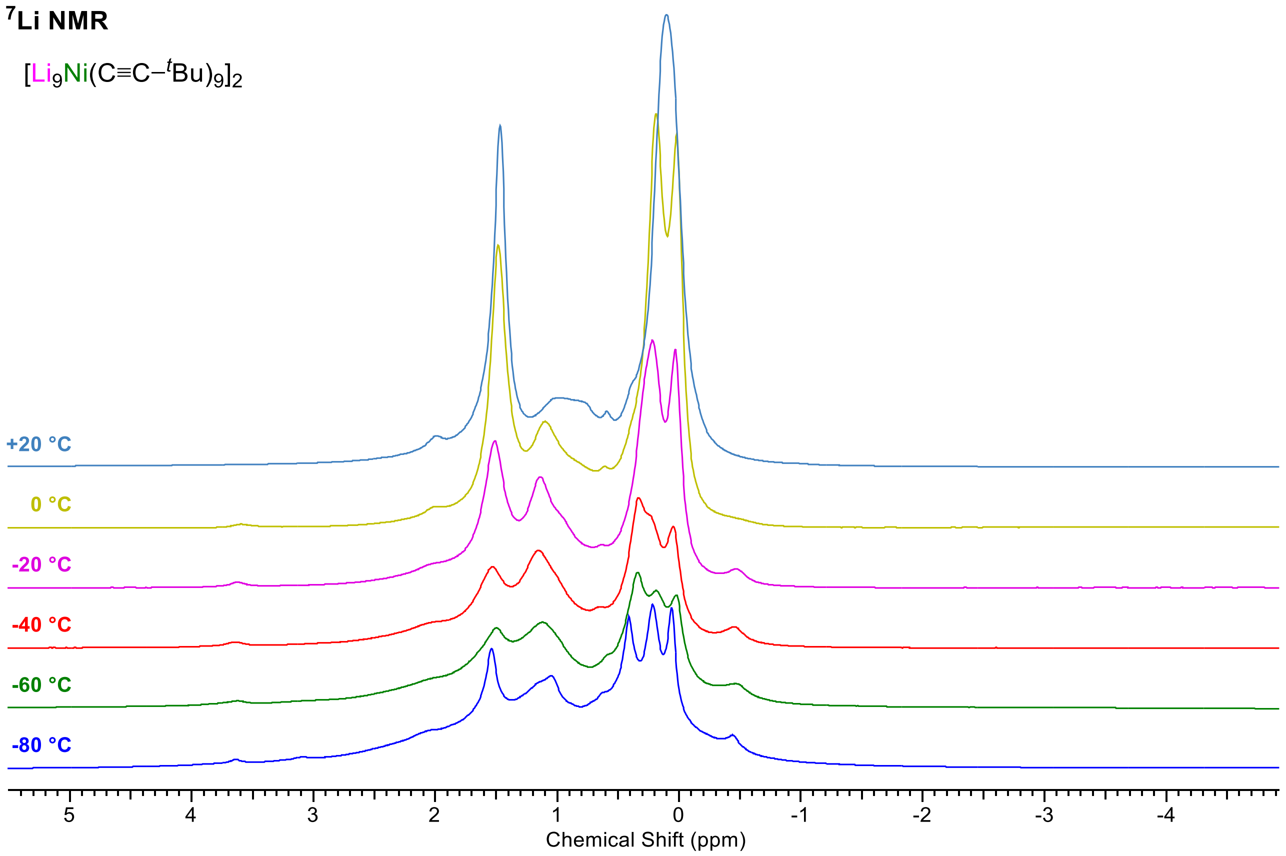

Figure S21: Stacked ^{7}Li NMR spectra of [$Li_9Ni(C≡C–^tBu)_9]_2$ (**2**) in toluene-d$_8$ at variable temperatures.

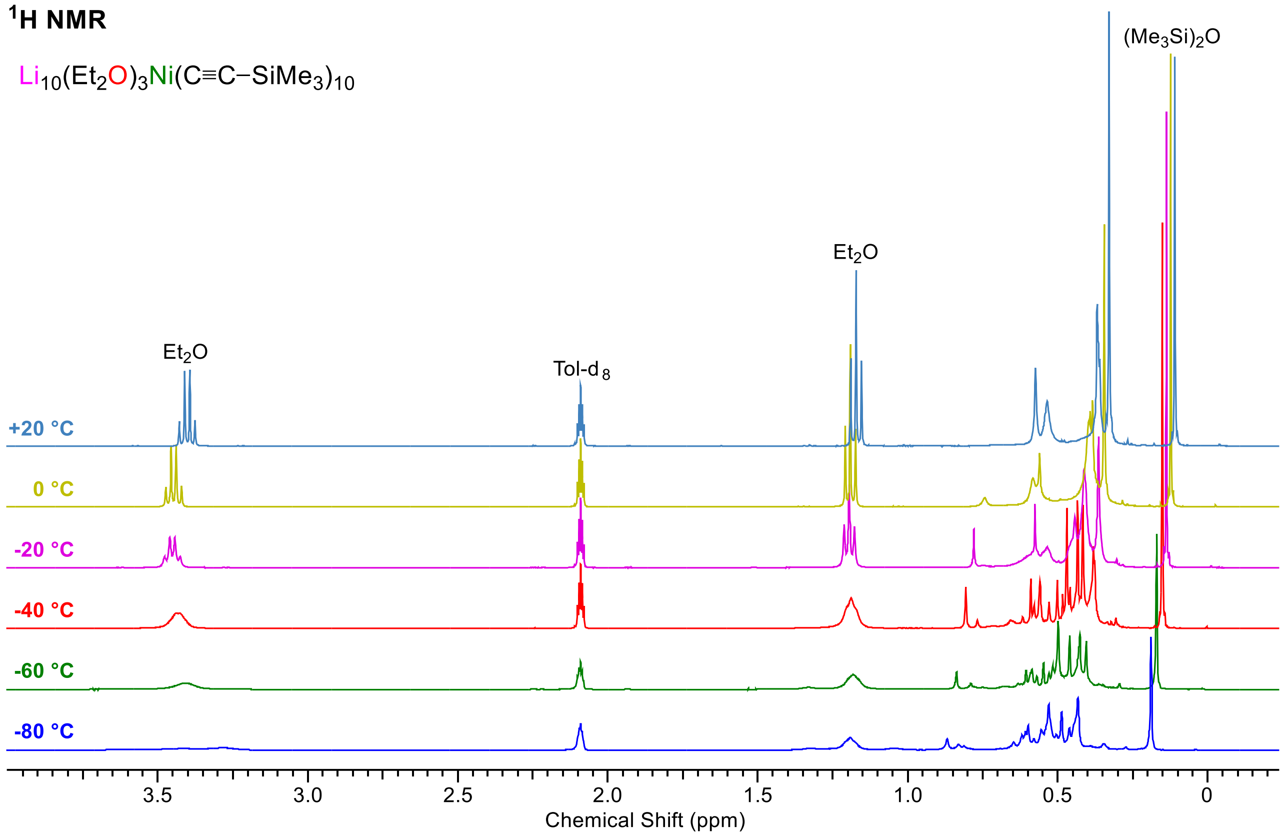

Figure S22: Stacked ^{1}H NMR spectra of $Li_{10}(Et_2O)_3Ni(C{\equiv}C{-}SiMe_3)_{10}$ (**3**) in toluene-d_8 at variable temperatures.

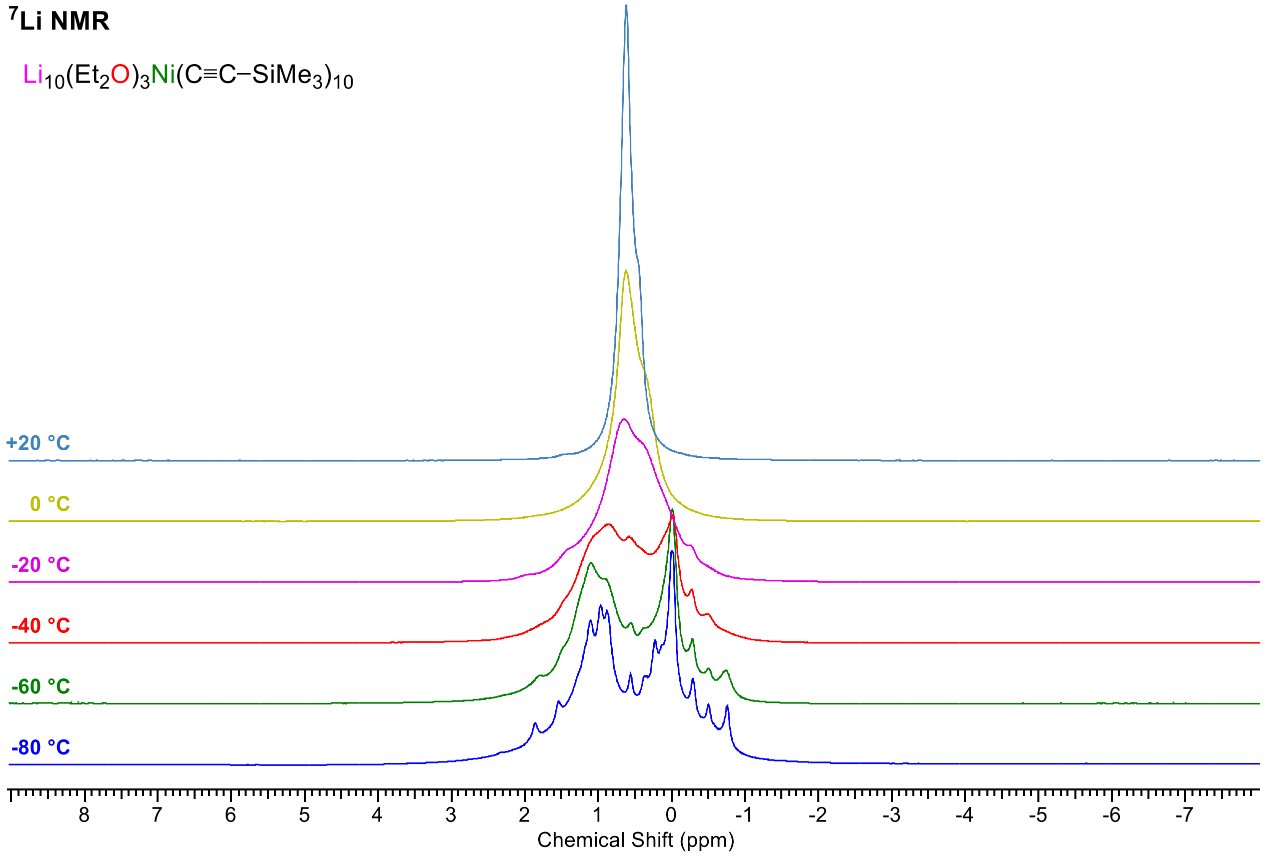

Figure S23: Stacked ^{7}Li NMR spectra of $Li_{10}(Et_2O)_3Ni(C{\equiv}C{-}SiMe_3)_{10}$ (**3**) in toluene-d_8 at variable temperatures.

X-Ray Crystallography

The crystal structures of all novel compounds have been deposited into the Cambridge Crystallographic Data Centre (CCDC) and have been assigned the following numbers: **1** – 2254773; **2** – 2254774; **3** – 2254775; **4** – 2254776; **5** –2254777; **6** – 2254778; **7** – 2254779; **8** – 2254780. Selected crystallographic and refinement parameters are presented below (**Tables S1–4**). In all cases, crystals immersed in an inert parabar oil were mounted at low temperatures and transferred into the nitrogen stream (100 or 173 K). Perfluorinated oils should be avoided for the lithium nickelates.

All measurements were made on a *RIGAKU Synergy S* area-detector diffractometer using mirror optics monochromated Cu *K*α radiation (λ = 1.54184 Å) or on a *RIGAKU XtaLAB Synergy R*, HyPix-Arc 100 area-detector diffractometer using mirror optics monochromated Mo *K*α radiation (λ = 0.71073 Å). Data reduction was performed using the *CrysAlisPro* program.[11] The intensities were corrected for Lorentz and polarization effects, and an absorption correction based on the Gaussian method using SCALE3 ABSPACK in *CrysAlisPro* was applied. The structure was solved by direct methods or intrinsic phasing using *SHELXT*,[12] which revealed the positions of all non-hydrogen atoms of the compounds. All non-hydrogen atoms were refined anisotropically. H-atoms were assigned in geometrically calculated positions and refined using a riding model where each H-atom was assigned a fixed isotropic displacement parameter with a value equal to 1.2Ueq of its parent atom (1.5Ueq for methyl groups). Refinement of the structure was carried out on F^2 using full-matrix least-squares procedures, which minimized the function $\Sigma w(F_o^2 - F_c^2)^2$. The weighting scheme was based on counting statistics and included a factor to downweight the intense reflections. All calculations were performed using the *SHELXL-2014/7*[13] program in OLEX2.[14]

For $[Li_{10}(Et_2O)_4(C{\equiv}C{-}^tBu)_{10}]$ (**1**), a disorder model was used for parts of the structure where the occupancies of each disorder component was refined through the use of a free variable. The sum of equivalent components was constrained to 1, i.e. 100%. The structure has been checked for void areas, however none could be found. The low density connects well with the dynamic disorder behavior in the structure. Twinning can be detected at post refinement steps, however the inclusion of the twin law, did not improve the refinement.

For $[Li_9Ni(C{\equiv}C{-}^tBu)_9]_2$ (**2**), Disorder model for parts of the structure where the occupancies of each disorder component was refined through the use of a free variable. The sum of equivalent components was constrained to 1, i.e. 100%. Areas containing disorder solvents were found where a satisfactory solvent model could not be achieved, therefore, a solvent mask was use The structure shows signs of twinning however a satisfactory twin law could not be found, leading to high final residual densities and R statistics.

For $Li_{10}(Et_2O)_3Ni(C{\equiv}C{-}SiMe_3)_{10}$ (**3**), a disorder model was used for parts of the structure where the occupancies of each disorder component was refined through the use of a free variable. The sum of equivalent components was constrained to 1, i.e. 100%. Due to density warnings, a solvent mask was used to locate voids but these did not contain any electron density inside. The structure was refined as an inversion twin due to uncertainty with the Flack parameter.

For $Li_{10}(Et_2O)_3Ni(C{\equiv}C–{}^tBu)_{10}$ (**4**), a disorder model was used for parts of the structure where the occupancies of each disorder component was refined through the use of a free variable. The sum of equivalent components was constrained to 1, i.e. 100%. Areas containing disorder solvents were found where a satisfactory solvent model could not be achieved, therefore, a solvent mask was used to include the contribution of electron density found in void areas into the calculated structure factor.

For $[Li_{11}(Et_2O)Ni_2(C{\equiv}C–{}^tBu)_{11}]_2$ (**5**), a disorder model was used for parts of the structure where the occupancies of each disorder component was refined through the use of a free variable. The sum of equivalent components was constrained to 1, i.e. 100%. Areas containing disordered solvents were found where a satisfactory solvent model could not be achieved, therefore, a solvent mask was used to include the contribution of electron density found in void areas into the calculated structure factor. The cluster contains regions in which the Li atoms are occupationally disordered across one or more positions in the solid-state structure where the sum of equivalent components was constrained to 1, i.e. 100%.

For $[Li_{10}(Et_2O)_2Ni(C{\equiv}C–{}^iPr)_8(C{\equiv}C–Me_2O)]_2$ (**6**), a disorder model was used for parts of the structure where the occupancies of each disorder component was refined through the use of a free variable. The sum of equivalent components was constrained to 1, i.e. 100%. Areas containing disordered solvents were found where a satisfactory solvent model could not be achieved, therefore, a solvent mask was used to include the contribution of electron density found in void areas into the calculated structure factor.

For $[Li_2(Et_2O)Ni(C{\equiv}C–{}^tBu)_4]_2$ (**7**), a disorder model was used for parts of the structure where the occupancies of each disorder component was refined through the use of a free variable. The sum of equivalent components was constrained to 1, i.e. 100%. Areas containing disorder solvents were found where a satisfactory solvent model could not be achieved, therefore, a solvent mask was used to include the contribution of electron density found in void areas into the calculated structure factor.

Identification code	**1**	**2**
CCDC deposition number	2254773	2254774
Empirical formula	$C_{76}H_{130}Li_{10}O_4$	$C_{108}H_{162}Li_{18}Ni_2$
Formula weight	1177.19	1702.71
Temperature/K	173.00(10)	173.00(10)
Crystal system	orthorhombic	monoclinic
Space group	Pbcn	C2/c
a/Å	25.2062(3)	25.0407(2)
b/Å	17.8835(2)	16.07277(13)
c/Å	19.3146(2)	30.7078(3)
α/°	90	90
β/°	90	97.1762(9)
γ/°	90	90
Volume/Å^3	8706.54(17)	12262.24(19)
Z	4	4
ρ_{calc}g/cm^3	0.898	0.922
μ/mm^{-1}	0.376	0.634
F(000)	2592	3680
Crystal size/mm^3	0.219 × 0.173 × 0.14	0.684 × 0.092 × 0.073
Radiation	Cu Kα (λ = 1.54184)	Cu Kα (λ = 1.54184)
2Θ range for data collection/°	6.06 to 148.984	6.55 to 136.496
Index ranges	-30 ≤ h ≤ 31, -22 ≤ k ≤ 22, -20 ≤ l ≤ 24	-30 ≤ h ≤ 28, -19 ≤ k ≤ 19, -36 ≤ l ≤ 36
Reflections collected	87773	92887
Independent reflections	8901 [R_{int} = 0.0389, R_{sigma} = 0.0237]	11228 [R_{int} = 0.0344, R_{sigma} = 0.0187]
Data/restraints/parameters	8901/315/571	11228/255/700
Goodness-of-fit on F^2	1.095	1.096
Final R indexes [I>=2σ (I)]	R_1 = 0.0881, wR_2 = 0.2962	R_1 = 0.1149, wR_2 = 0.3202
Final R indexes [all data]	R_1 = 0.1070, wR_2 = 0.3268	R_1 = 0.1174, wR_2 = 0.3218
Largest diff. peak/hole / e Å^{-3}	0.30/-0.31	1.87/-0.38
Flack parameter	-	-

Table S1: Crystal data and structure refinement details for compounds **1** and **2**.

Identification code	**3**	**4**
CCDC deposition number	2254775	2254776
Empirical formula	$C_{68}H_{138}Li_{10}NiO_4Si_{12}$	$C_{72}H_{120}Li_{10}NiO_3$
Formula weight	1484.97	1161.78
Temperature/K	100.01(10)	173.00(10)
Crystal system	monoclinic	triclinic
Space group	$P2_1$	P-1
a/Å	16.57914(5)	13.57043(8)
b/Å	20.21889(6)	14.17883(8)
c/Å	29.33071(10)	24.50395(10)
α/°	90	87.7356(4)
β/°	91.9914(3)	77.7715(4)
γ/°	90	62.5608(6)
Volume/Å^3	9826.06(5)	4080.33(4)
Z	4	2
ρ_{calc}g/cm^3	1.004	0.946
μ/mm^{-1}	1.949	0.602
F(000)	3216	1268
Crystal size/mm^3	0.626 × 0.335 × 0.202	0.398 × 0.235 × 0.175
Radiation	Cu Kα (λ = 1.54184)	Cu Kα (λ = 1.54184)
2Θ range for data collection/°	5.31 to 140.148	7.04 to 148.992
Index ranges	-20 ≤ h ≤ 20, -24 ≤ k ≤ 24, -30 ≤ l ≤ 35	-16 ≤ h ≤ 14, -17 ≤ k ≤ 17, -30 ≤ l ≤ 30
Reflections collected	378395	158617
Independent reflections	37320 [R_{int} = 0.0512, R_{sigma} = 0.0198]	16611 [R_{int} = 0.0205, R_{sigma} = 0.0095]
Data/restraints/parameters	37320/308/1987	16611/281/1014
Goodness-of-fit on F^2	1.02	1.041
Final R indexes [I>=2σ (I)]	R_1 = 0.0320, wR_2 = 0.0853	R_1 = 0.0443, wR_2 = 0.1302
Final R indexes [all data]	R_1 = 0.0325, wR_2 = 0.0859	R_1 = 0.0460, wR_2 = 0.1319
Largest diff. peak/hole / e Å^{-3}	0.63/-0.34	0.51/-0.33
Flack parameter	0.126(11)	-

Table S2: Crystal data and structure refinement details for compounds **3** and **4**.

Identification code	**5**	**6**
CCDC deposition number	2254777	2254778
Empirical formula	$C_{140}H_{218}Li_{22}Ni_4O_2$	$C_{106}H_{164}Li_{20}Ni_2O_6$
Formula weight	2320.65	1790.58
Temperature/K	173.00(10)	173.01(10)
Crystal system	triclinic	triclinic
Space group	P-1	P-1
a/Å	14.72420(10)	13.0955(2)
b/Å	14.85270(10)	13.51440(10)
c/Å	19.58560(10)	17.7720(2)
α/°	87.5880(10)	75.6700(10)
β/°	83.0760(10)	81.8670(10)
γ/°	85.4260(10)	75.3240(10)
Volume/Å^3	4236.19(5)	2937.54(6)
Z	1	1
ρ_{calc}g/cm^3	0.91	1.012
μ/mm^{-1}	0.767	0.718
F(000)	1252	964
Crystal size/mm^3	0.204 × 0.125 × 0.062	0.331 × 0.294 × 0.148
Radiation	Cu Kα (λ = 1.54184)	Cu Kα (λ = 1.54184)
2Θ range for data collection/°	4.548 to 149.006	5.15 to 149
Index ranges	-18 ≤ h ≤ 17, -18 ≤ k ≤ 18, -24 ≤ l ≤ 24	-16 ≤ h ≤ 13, -16 ≤ k ≤ 16, -22 ≤ l ≤ 22
Reflections collected	164874	114169
Independent reflections	17286 [R_{int} = 0.0345, R_{sigma} = 0.0162]	11964 [R_{int} = 0.0349, R_{sigma} = 0.0154]
Data/restraints/parameters	17286/344/995	11964/86/677
Goodness-of-fit on F^2	1.067	1.045
Final R indexes [I>=2σ (I)]	R_1 = 0.0569, wR_2 = 0.1668	R_1 = 0.0798, wR_2 = 0.2409
Final R indexes [all data]	R_1 = 0.0602, wR_2 = 0.1701	R_1 = 0.0832, wR_2 = 0.2455
Largest diff. peak/hole / e Å^{-3}	0.87/-0.64	1.40/-1.00
Flack parameter	-	-

Table S3: Crystal data and structure refinement details for compounds **5** and **6**.

Identification code	**7**	**8**
CCDC deposition number	2254779	2254780
Empirical formula	$C_{56}H_{92}Li_4Ni_2O_2$	$C_{28}H_{56}Li_2NiO_2Si_4$
Formula weight	942.47	609.67
Temperature/K	173.00(10)	99.98(10)
Crystal system	monoclinic	monoclinic
Space group	$P2_1/n$	$P2_1/c$
a/Å	11.42022(4)	12.60922(13)
b/Å	17.31117(7)	13.16095(10)
c/Å	34.29155(13)	12.68620(13)
α/°	90	90
β/°	98.1033(3)	114.8430(12)
γ/°	90	90
Volume/Å^3	6711.67(4)	1910.45(4)
Z	4	2
ρ_{calc}g/cm^3	0.933	1.06
μ/mm^{-1}	0.909	0.653
F(000)	2048	660
Crystal size/mm^3	0.238 × 0.16 × 0.111	0.238 × 0.213 × 0.172
Radiation	Cu Kα (λ = 1.54184)	Mo Kα (λ = 0.71073)
2Θ range for data collection/°	5.206 to 136.492	6.45 to 61.014
Index ranges	-13 ≤ h ≤ 13, -19 ≤ k ≤ 20, -41 ≤ l ≤ 41	-18 ≤ h ≤ 18, -18 ≤ k ≤ 18, -18 ≤ l ≤ 18
Reflections collected	140067	114183
Independent reflections	12280 [R_{int} = 0.0283, R_{sigma} = 0.0159]	5815 [R_{int} = 0.0295, R_{sigma} = 0.0104]
Data/restraints/parameters	12280/31/636	5815/6/177
Goodness-of-fit on F^2	1.053	1.082
Final R indexes [I>=2σ (I)]	R_1 = 0.0333, wR_2 = 0.0977	R_1 = 0.0208, wR_2 = 0.0587
Final R indexes [all data]	R_1 = 0.0351, wR_2 = 0.0995	R_1 = 0.0227, wR_2 = 0.0596
Largest diff. peak/hole / e Å^{-3}	0.43/-0.23	0.59/-0.15
Flack parameter	-	-

Table S4: Crystal data and structure refinement details for compounds **7** and **8**.

Molecular Structure of $[Li_{10}(Et_2O)_4(C{\equiv}C{-}^tBu)_{10}]$ (1)

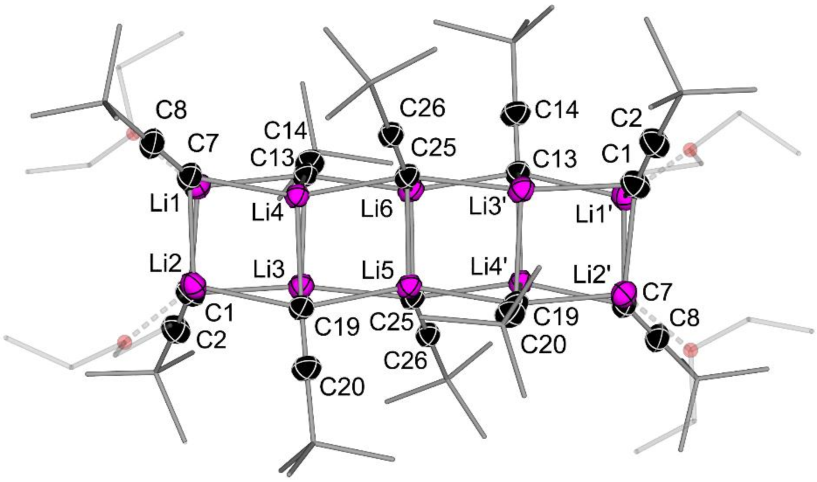

Figure S24: Molecular structure of $[Li_{10}(Et_2O)_4(C{\equiv}C{-}^tBu)_{10}]$ (**1**). Thermal ellipsoids shown at 30% probability. Hydrogen atoms omitted and ^{t}Bu groups and coordinated Et_2O shown as wireframes for clarity.

X–Y	Bond Length (Å)	X–Y	Bond Length (Å)
Li1–C1	2.217(3)	Li4–C19	2.230(3)
Li1–C7	2.212(3)	Li4–C25	2.265(3)
Li1–C13	2.216(3)	Li5–C19	2.1914(17)
Li2–C1	2.222(3)	Li5–C25	2.237(2)
Li2–C7	2.212(3)	Li6–C13	2.2139(16)
Li2–C19	2.204(3)	Li6–C25	2.234(2)
Li3–C1	2.272(4)	C1–C2	1.218(3)
Li3–C13	2.264(3)	C7–C8	1.211(3)
Li3–C19	2.270(3)	C13–C14	1.214(2)
Li3–C25	2.295(3)	C19–C20	1.216(2)
Li4–C7	2.200(3)	C25–C26	1.213(2)
Li4–C13	2.264(4)		

Table S5: Selected bond lengths in $[Li_{10}(Et_2O)_4(C{\equiv}C{-}^tBu)_{10}]$ (**1**).

Molecular Structure of $[Li_9Ni(C{\equiv}C{-}^tBu)_9]_2$ (2)

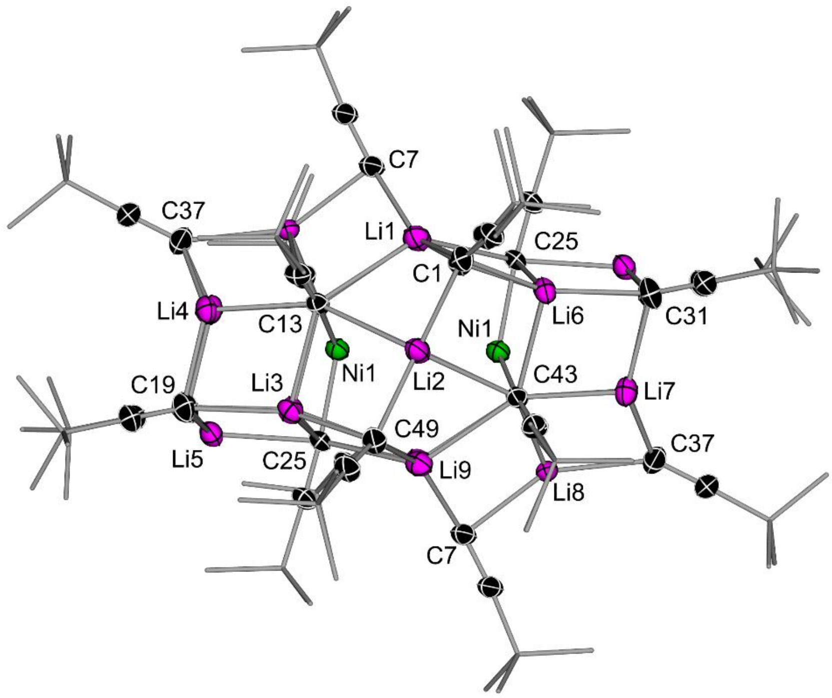

Figure S25: Molecular structure of $[Li_9Ni(C{\equiv}C{-}^tBu)_9]_2$ (**2**) showing the full lithium nickelate cluster. Thermal ellipsoids shown at 30% probability. Hydrogen atoms omitted and ^{t}Bu groups shown as wireframes for clarity.

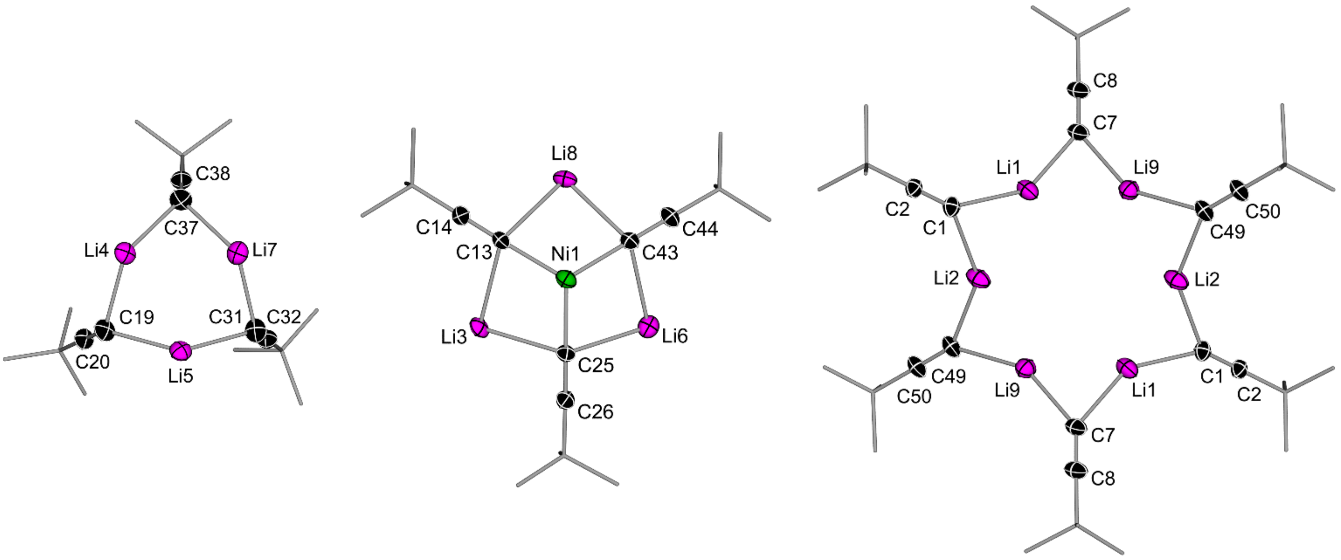

Figure S26: Molecular structure of $[Li_9Ni(C{\equiv}C{-}^tBu)_9]_2$ (**2**) showing the three unit building blocks. Thermal ellipsoids shown at 30% probability. Hydrogen atoms omitted and ^{t}Bu groups shown as wireframes for clarity.

X–Y	Bond Length (Å)	X–Y	Bond Length (Å)
Ni1–C13	1.907(5)	Li6–C31	2.17(1)
Ni1–C25	1.912(5)	Li6–C43	2.31(1)
Ni1–C43	1.907(5)	Li7–C31	2.13(1)
Li1–C1	2.17(1)	Li7–C37	2.11(1)
Li1–C7	2.16(1)	Li7–C43	2.23(1)
Li1–C13	2.46(1)	Li8–C7	2.152(9)
Li1–C25	2.47(1)	Li8–C13	2.306(9)
Li2–C1	2.19(1)	Li8–C37	2.221(9)
Li2–C13	2.43(1)	Li8–C43	2.290(9)
Li2–C43	2.47(1)	Li9–C7	2.20(1)
Li2–C49	2.15(1)	Li9–C25	2.44(1)
Li3–C13	2.339(9)	Li9–C43	2.44(1)
Li3–C19	2.17(1)	Li9–C49	2.23(1)
Li3–C25	2.30(1)	C1–C2	1.219(7)
Li3–C49	2.15(1)	C7–C8	1.215(7)
Li4–C13	2.28(1)	C13–C14	1.217(6)
Li4–C19	2.12(1)	C19–C20	1.207(7)
Li4–C37	2.12(1)	C25–C26	1.231(7)
Li5–C19	2.11(1)	C31–C32	1.181(8)
Li5–C26	2.33(1)	C37–C38	1.203(7)
Li5–C31	2.13(1)	C43–C44	1.221(7)
Li6–C1	2.15(1)	C49–C50	1.205(7)
Li6–C25	2.28(1)		

Table S6: Selected bond lengths in $[Li_9Ni(C{\equiv}C{-}^tBu)_9]_2$ (**2**).

Molecular Structure of $Li_{10}(Et_2O)_3Ni(C{\equiv}C–SiMe_3)_{10}$ (3)

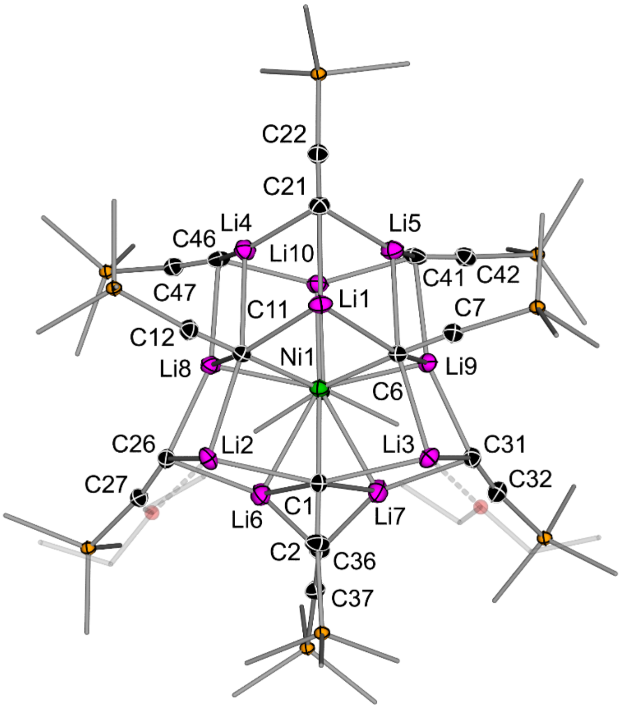

Figure S27: Molecular structure of $Li_{10}(Et_2O)_3Ni(C{\equiv}C–SiMe_3)_{10}$ (**3**) showing a top-down view along the Ni1–C16 bond. Thermal ellipsoids shown at 30% probability. Hydrogen atoms and one coordinated Et_2O omitted and $SiMe_3$ groups and coordinated Et_2O shown as wireframes for clarity. Only one molecule of the asymmetric is shown, and co-crystallised $(Me_3Si)_2O$ has been removed.

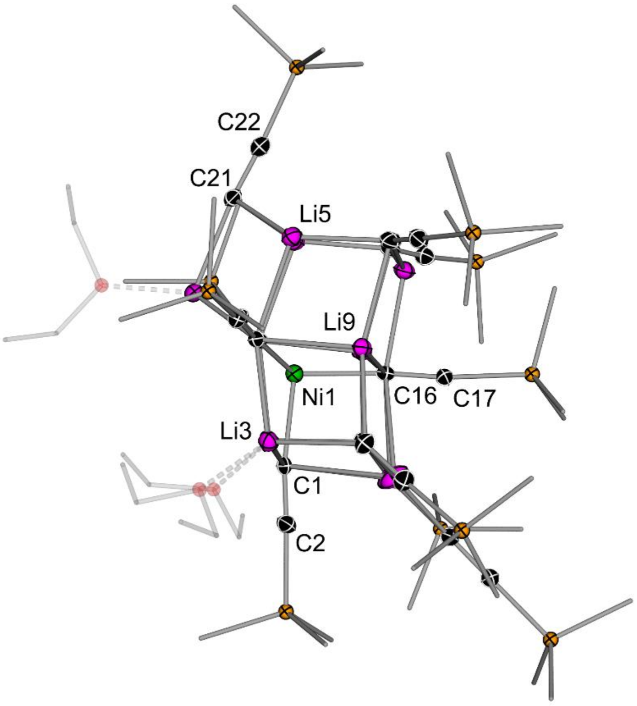

Figure S28: Molecular structure of $Li_{10}(Et_2O)_3Ni(C{\equiv}C–SiMe_3)_{10}$ (**3**) showing a side-on view of the cluster. Thermal ellipsoids shown at 30% probability. Hydrogen atoms omitted and $SiMe_3$ groups and coordinated Et_2O shown as wireframes for clarity. Only one molecule of the asymmetric is shown, and co-crystallised $(Me_3Si)_2O$ has been removed.

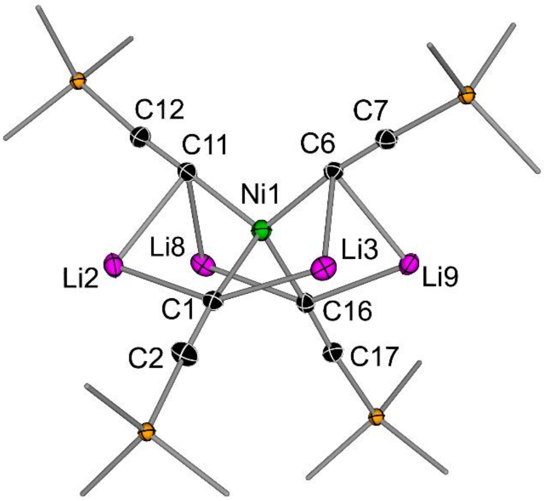

Figure S29: Molecular structure of $Li_{10}(Et_2O)_3Ni(C{\equiv}C–SiMe_3)_{10}$ (**3**) showing the tetra-lithium nickelate core. Thermal ellipsoids shown at 30% probability. Hydrogen atoms omitted and $SiMe_3$ groups and coordinated Et_2O shown as wireframes for clarity.

X–Y	Bond Length (Å)	X–Y	Bond Length (Å)
Ni1–C1	1.941(2)	Li7–C31	2.153(5)
Ni1–C6	1.904(2)	Li7–C36	2.112(5)
Ni1–C11	1.901(2)	Li8–C11	2.299(5)
Ni1–C16	1.911(2)	Li8–C16	2.355(5)
Li1–C6	2.302(5)	Li8–C26	2.144(5)
Li1–C11	2.291(5)	Li8–C46	2.287(5)
Li1–C21	2.156(5)	Li9–C6	2.296(5)
Li2–C1	2.333(5)	Li9–C16	2.336(5)
Li2–C11	2.252(5)	Li9–C31	2.180(5)
Li2–C26	2.201(5)	Li9–C41	2.303(5)
Li3–C1	2.326(5)	Li10–C16	2.198(5)
Li3–C6	2.227(5)	Li10–C41	2.126(6)
Li3–C31	2.210(5)	Li10–C46	2.198(5)
Li4–C11	2.257(5)	C1–C2	1.233(4)
Li4–C21	2.157(5)	C6–C7	1.238(3)
Li4–C46	2.106(5)	C11–C12	1.247(4)
Li5–C6	2.244(5)	C16–C17	1.243(3)
Li5–C21	2.143(6)	C21–C22	1.222(4)
Li5–C41	2.108(5)	C26–C27	1.219(3)
Li6–C16	2.538(6)	C31–C32	1.210(4)
Li6–C26	2.167(5)	C36–C37	1.215(3)
Li6–C36	2.132(6)	C41–C42	1.214(4)
Li7–C16	2.482(6)	C46–C47	1.219(4)

Table S7: Selected bond lengths in $Li_{10}(Et_2O)_3Ni(C{\equiv}C–SiMe_3)_{10}$ (**3**). Data provided for only one of the molecules in the asymmetric unit.

Molecular Structure of $Li_{10}(Et_2O)_3Ni(C{\equiv}C–{}^tBu)_{10}$ (4)

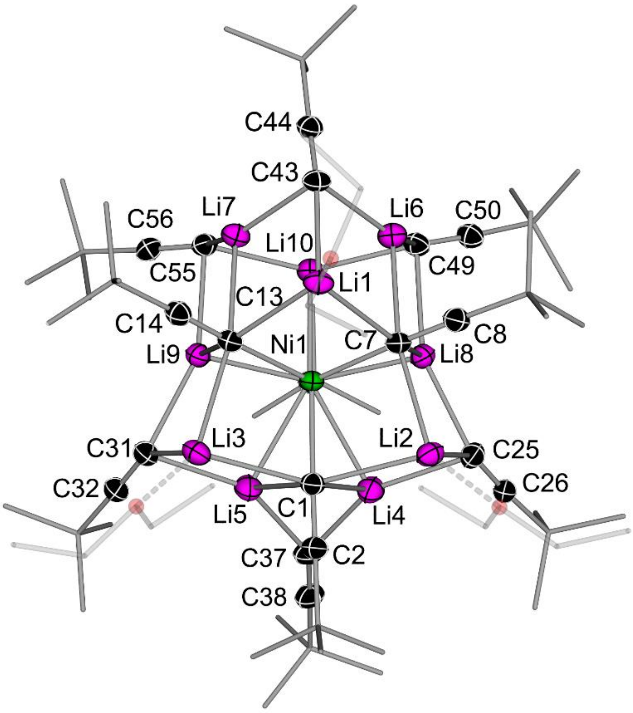

Figure S30: Molecular structure of $Li_{10}(Et_2O)_3Ni(C{\equiv}C–{}^tBu)_{10}$ (**4**) showing a top-down view along the Ni1–C19 bond. Thermal ellipsoids shown at 30% probability. Hydrogen atoms omitted and ^{t}Bu groups and coordinated Et_2O shown as wireframes for clarity.

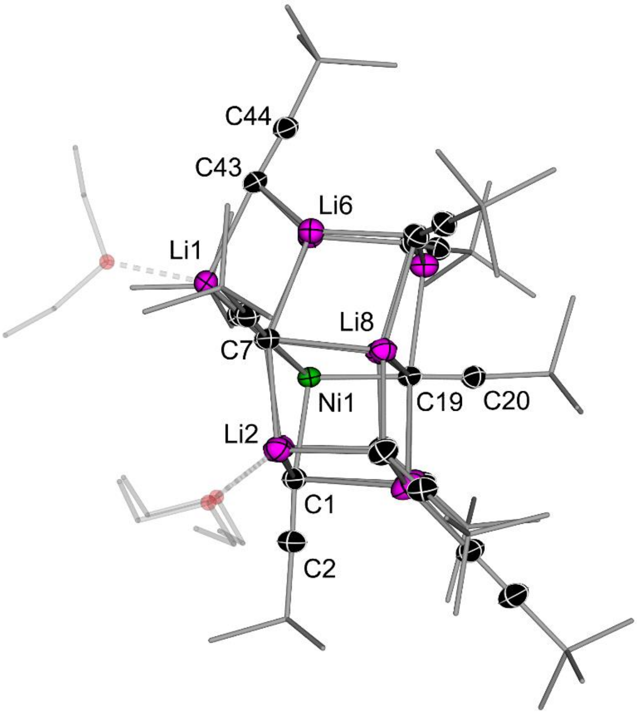

Figure S31: Molecular structure of $Li_{10}(Et_2O)_3Ni(C{\equiv}C–{}^tBu)_{10}$ (**4**) showing a side-on view of the cluster. Thermal ellipsoids shown at 30% probability. Hydrogen atoms omitted and ^{t}Bu groups and coordinated Et_2O shown as wireframes for clarity.

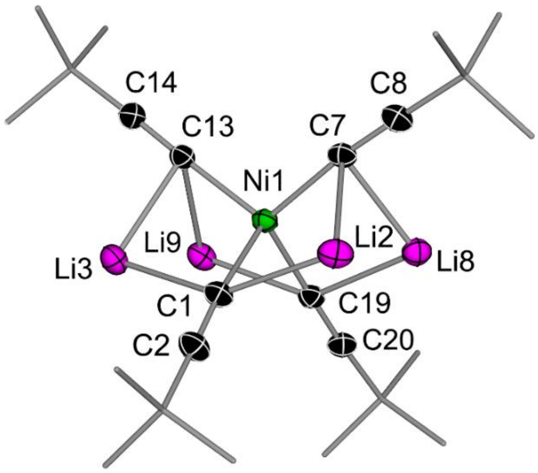

Figure S31: Molecular structure of $Li_{10}(Et_2O)_3Ni(C{\equiv}C–{}^tBu)_{10}$ (**4**) showing the tetra-lithium nickelate core. Thermal ellipsoids shown at 30% probability. Hydrogen atoms omitted and ^{t}Bu groups shown as wireframes for clarity.

X–Y	Bond Length (Å)	X–Y	Bond Length (Å)
Ni1–C1	1.984(1)	Li7–C43	2.114(3)
Ni1–C7	1.946(2)	Li7–C55	2.084(3)
Ni1–C13	1.948(1)	Li8–C17	2.267(3)
Ni1–C19	1.941(1)	Li8–C19	2.293(3)
Li1–C7	2.268(3)	Li8–C25	2.157(3)
Li1–C13	2.274(3)	Li8–C49	2.275(2)
Li1–C43	2.169(2)	Li9–C13	2.329(3)
Li2–C1	2.320(3)	Li9–C19	2.305(3)
Li2–C7	2.213(3)	Li9–C31	2.159(3)
Li2–C25	2.203(4)	Li9–C55	2.252(2)
Li3–C1	2.320(3)	Li10–C19	2.173(2)
Li3–C13	2.232(3)	Li10–C49	2.135(3)
Li3–C31	2.240(3)	Li10–C55	2.130(3)
Li4–C1	2.476(5)	C1–C2	1.222(2)
Li4–C25	2.130(3)	C7–C8	1.234(2)
Li4–C37	2.120(3)	C13–C14	1.234(3)
Li5–C1	2.533(3)	C19–C20	1.235(2)
Li5–C31	2.163(4)	C25–C26	1.210(3)
Li5–C37	2.121(4)	C31–C32	1.214(2)
Li6–C7	2.218(2)	C37–C38	1.208(3)
Li6–C43	2.095(4)	C43–C44	1.215(2)
Li6–C49	2.079(3)	C49–C50	1.211(3)
Li7–C13	2.217(2)	C55–C56	1.214(2)

Table S8: Selected bond lengths in $Li_{10}(Et_2O)_3Ni(C{\equiv}C–{}^tBu)_{10}$ (**4**).

Molecular Structure of $[Li_{11}(Et_2O)Ni_2(C{\equiv}C{-}^tBu)_{11}]_2$ (5)

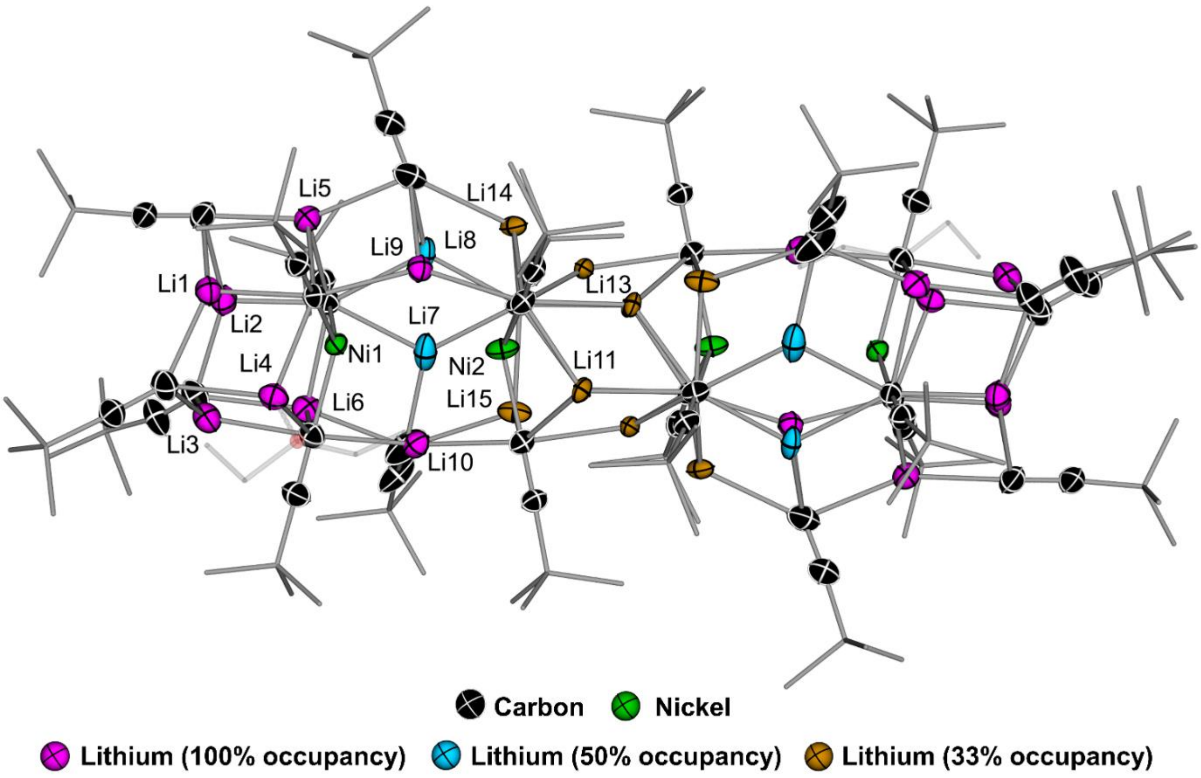

Figure S32: Molecular structure of $[Li_{11}(Et_2O)Ni_2(C{\equiv}C{-}^tBu)_{11}]_2$ (**5**). Thermal ellipsoids shown at 30% probability. Hydrogen atoms omitted and ^{t}Bu groups and coordinated Et_2O shown as wireframes for clarity.

Molecular Structure of $[Li_{10}(Et_2O)_2Ni(C{\equiv}C{-}^iPr)_8(C{\equiv}C{-}Me_2O)]_2$ (6)

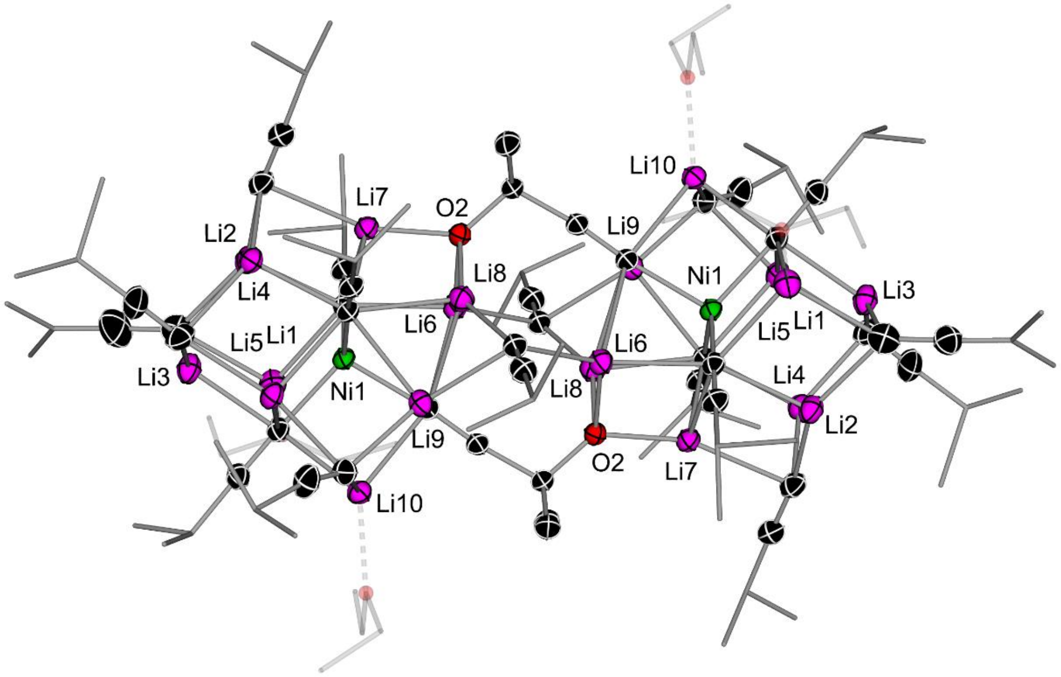

Figure S33: Molecular structure of $[Li_{10}(Et_2O)_2Ni(C{\equiv}C{-}^iPr)_8(C{\equiv}C{-}Me_2O)]_2$ (**6**). Thermal ellipsoids shown at 30% probability. Hydrogen atoms omitted and ^{i}Pr groups and coordinated Et_2O shown as wireframes for clarity.

Molecular Structure of [$Li_2(Et_2O)_2Ni(C{\equiv}C–^tBu)_4]_2$ (7)

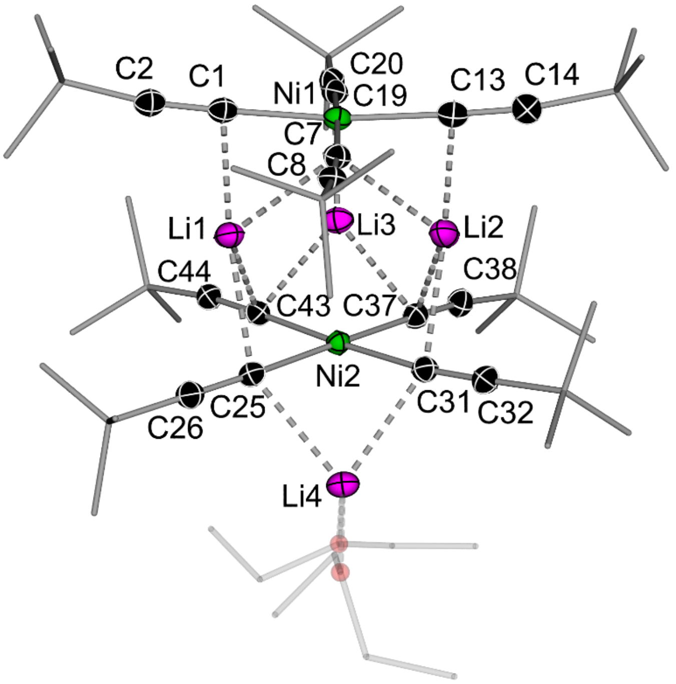

Figure S34: Molecular structure of [$Li_2(Et_2O)Ni(C{\equiv}C–^tBu)_4]_2$ (**7**). Thermal ellipsoids shown at 30% probability. Hydrogen atoms omitted and ^{t}Bu groups and coordinated Et_2O shown as wireframes for clarity.

X–Y	Bond Length (Å)	X–Y	Bond Length (Å)
Ni1–C1	1.885(1)	Li2–C31	2.248(3)
Ni1–C7	1.888(1)	Li3–C19	2.156(3)
Ni1–C13	1.189(1)	Li3–C37	2.204(3)
Ni1–C19	1.888(1)	Li3–C43	2.204(3)
Ni2–C25	1.894(1)	Li4–C25	2.317(3)
Ni2–C31	1.896(1)	Li4–C31	2.293(3)
Ni2–C37	1.872(1)	C1–C2	1.216(2)
Ni2–C43	1.871(1)	C7–C8	1.212(2)
Li1–C1	2.174(2)	C13–C14	1.217(2)
Li1–C7	2.352(3)	C19–C20	1.216(2)
Li1–C25	2.313(2)	C25–C26	1.214(2)
Li1–C43	2.462(3)	C31–C32	1.211(2)
Li2–C7	2.332(3)	C37–C38	1.212(2)
Li2–C13	2.154(3)	C43–C44	1.211(2)

Table S9: Selected bond lengths in [$Li_2(Et_2O)Ni(C{\equiv}C–^tBu)_4]_2$ (**7**).

Molecular Structure of $Li_2(Et_2O)_2Ni(C{\equiv}C–SiMe_3)_4$ (8)

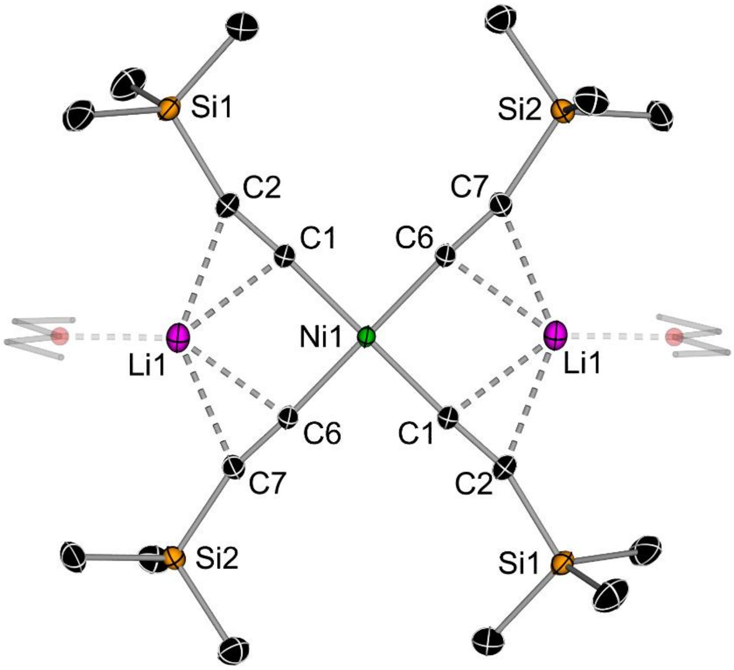

Figure S35: Molecular structure of $Li_2(Et_2O)_2Ni(C{\equiv}C–SiMe_3)_4$ (**8**). Thermal ellipsoids shown at 50% probability. Hydrogen atoms omitted and coordinated Et_2O shown as wireframes for clarity.

X–Y	Bond Length (Å)	X–Y	Bond Length (Å)
Ni1–C1	1.8707(7)	Li1–C6	2.212(2)
Ni1–C6	1.8695(7)	Li1–C7	2.334(2)
Li1–C1	2.210(1)	C1–C2	1.2328(10)
Li1–C2	2.333(2)	C6–C7	1.2318(11)

Table S10: Selected bond lengths in $Li_2(Et_2O)_2Ni(C{\equiv}C–SiMe_3)_4$ (**8**).

NMR Spectra of Reported Compounds

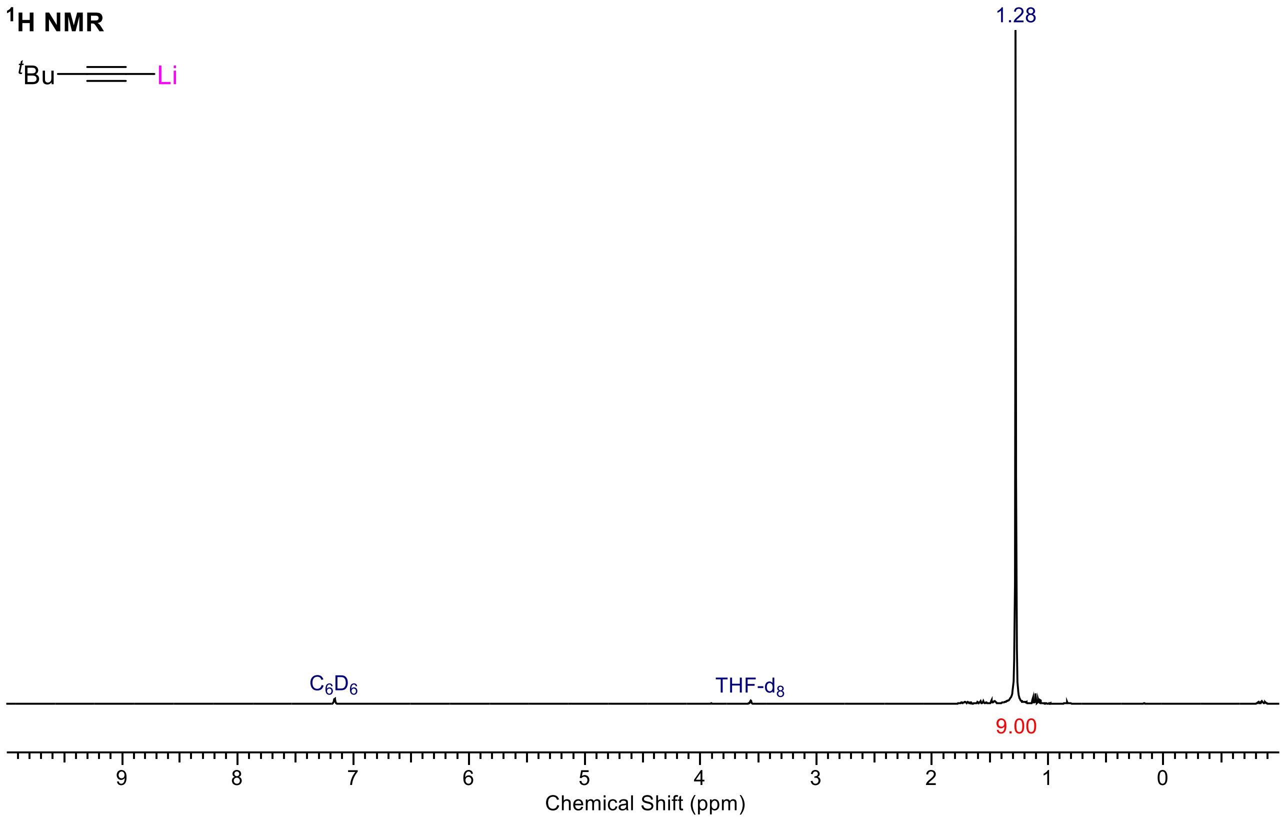

Spectra S1: ^{1}H NMR spectrum of ^{t}Bu–C≡C–Li in C_6D_6/THF-d_8.

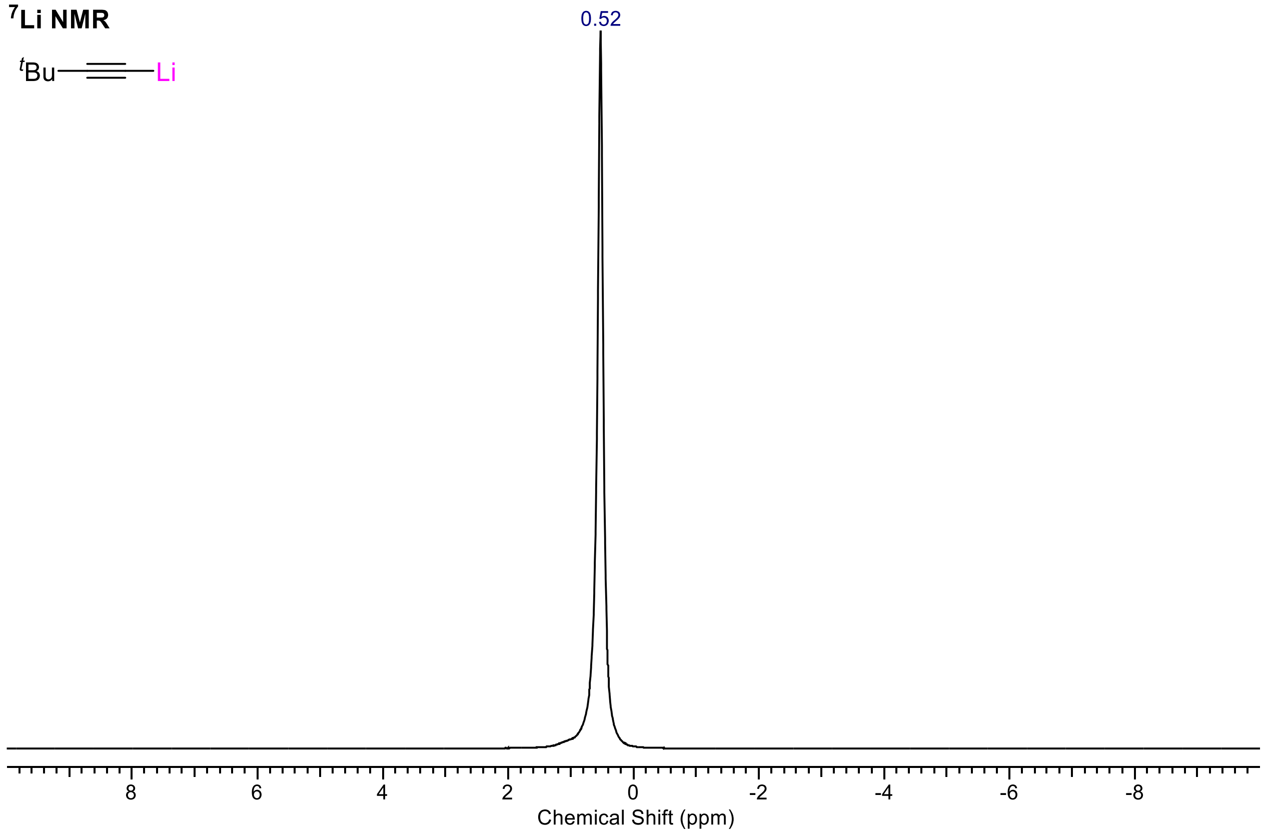

Spectra S2: ^{7}Li NMR spectrum of ^{t}Bu–C≡C–Li in C_6D_6/THF-d_8.

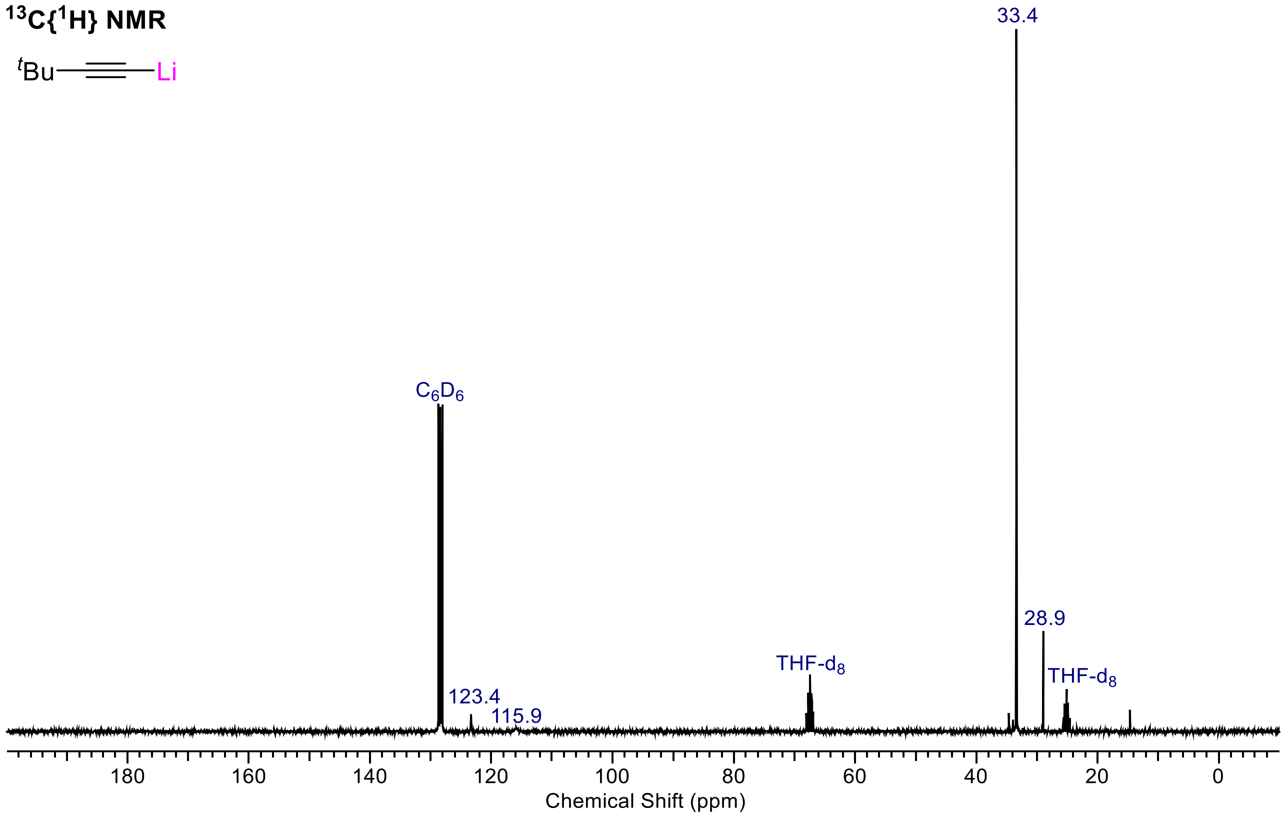

Spectra S3: $^{13}C\{^1H\}$ NMR spectrum of ^{t}Bu–C≡C–Li in C_6D_6/THF-d_8.

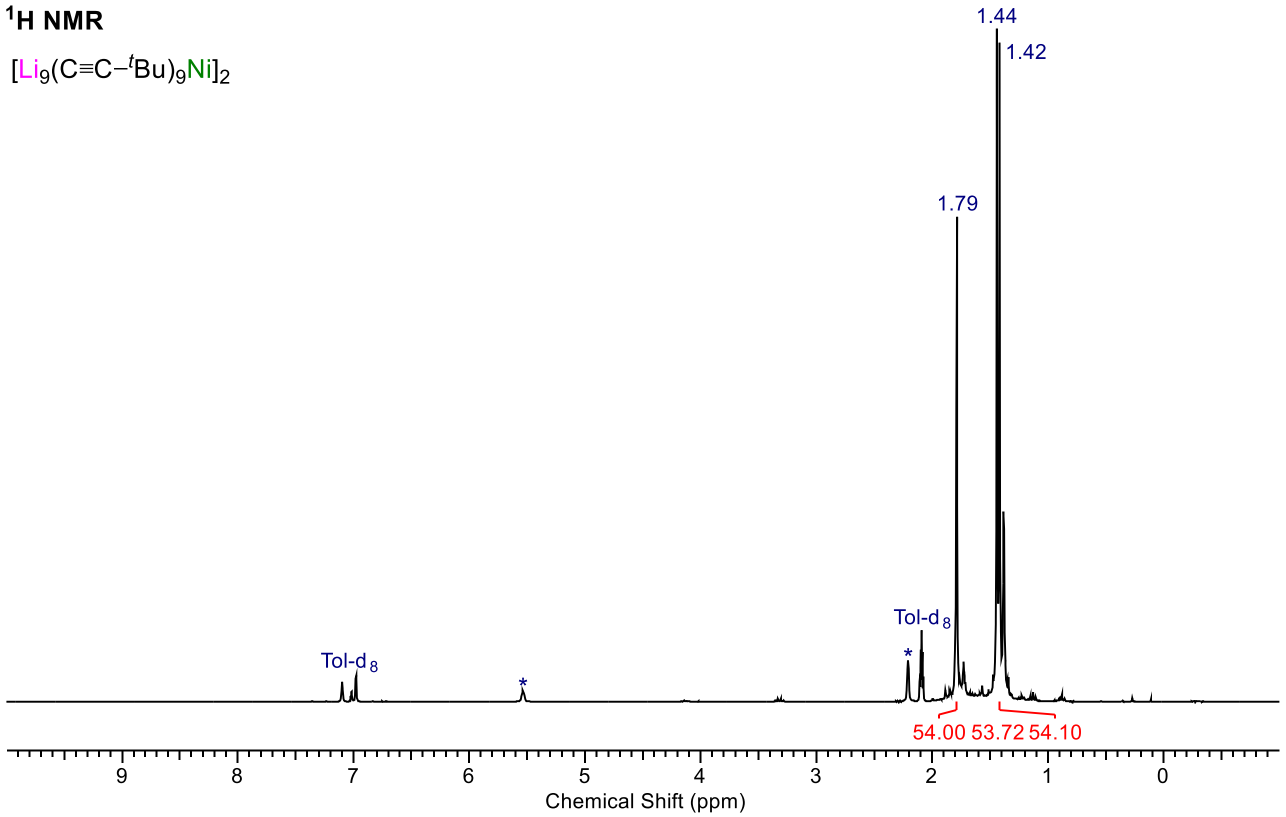

Spectra S4: ^{1}H NMR spectra of $[Li_9Ni(C{\equiv}C–^tBu)_9]_2$ (**2**) in toluene-d_8. * Trace COD.

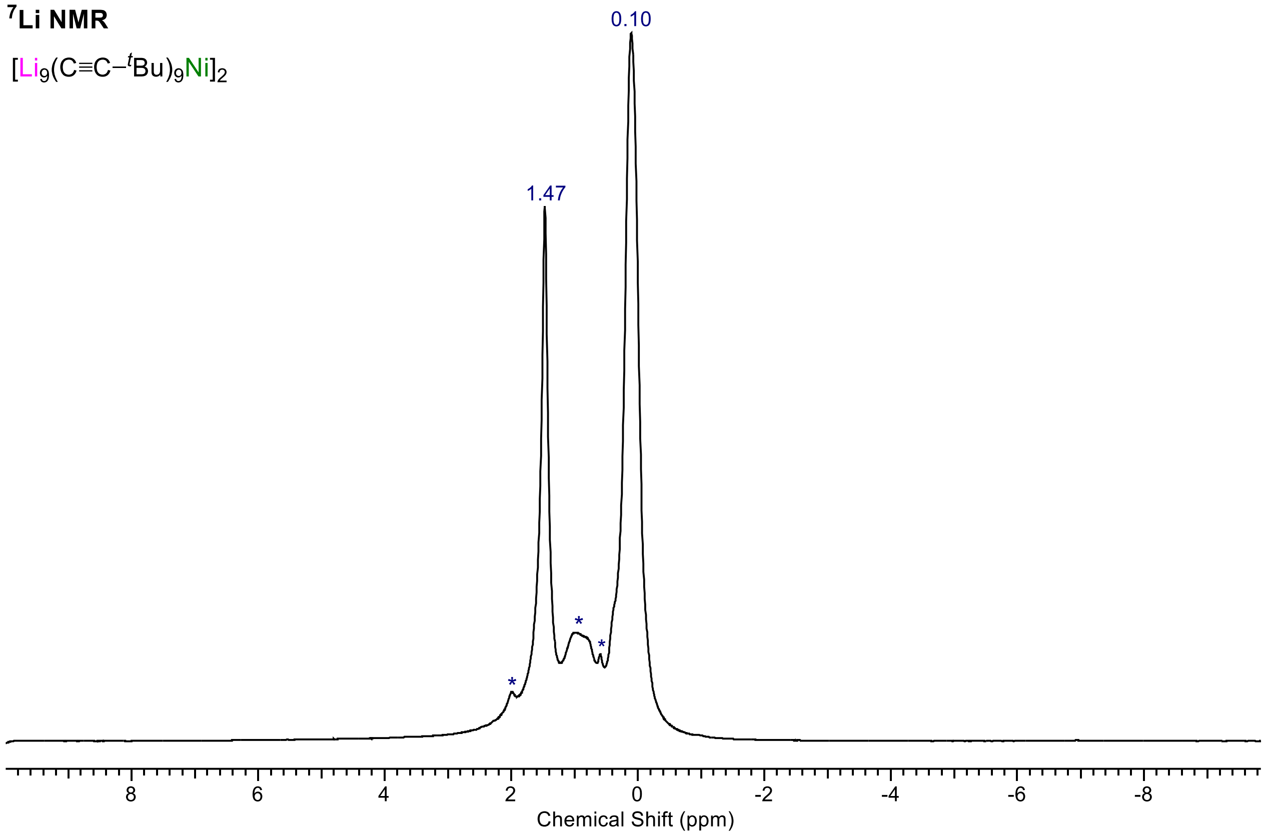

Spectra S5: ^{7}Li NMR spectra of [Li_9Ni(C≡C–^{t}Bu)$_9$]$_2$ (**2**) in toluene-d_8. * Trace impurities, possibly including ^{t}Bu–C≡C–Li and $Li_{10}(Et_2O)_3Ni$(C≡C–^{t}Bu)$_{10}$ (**4**).

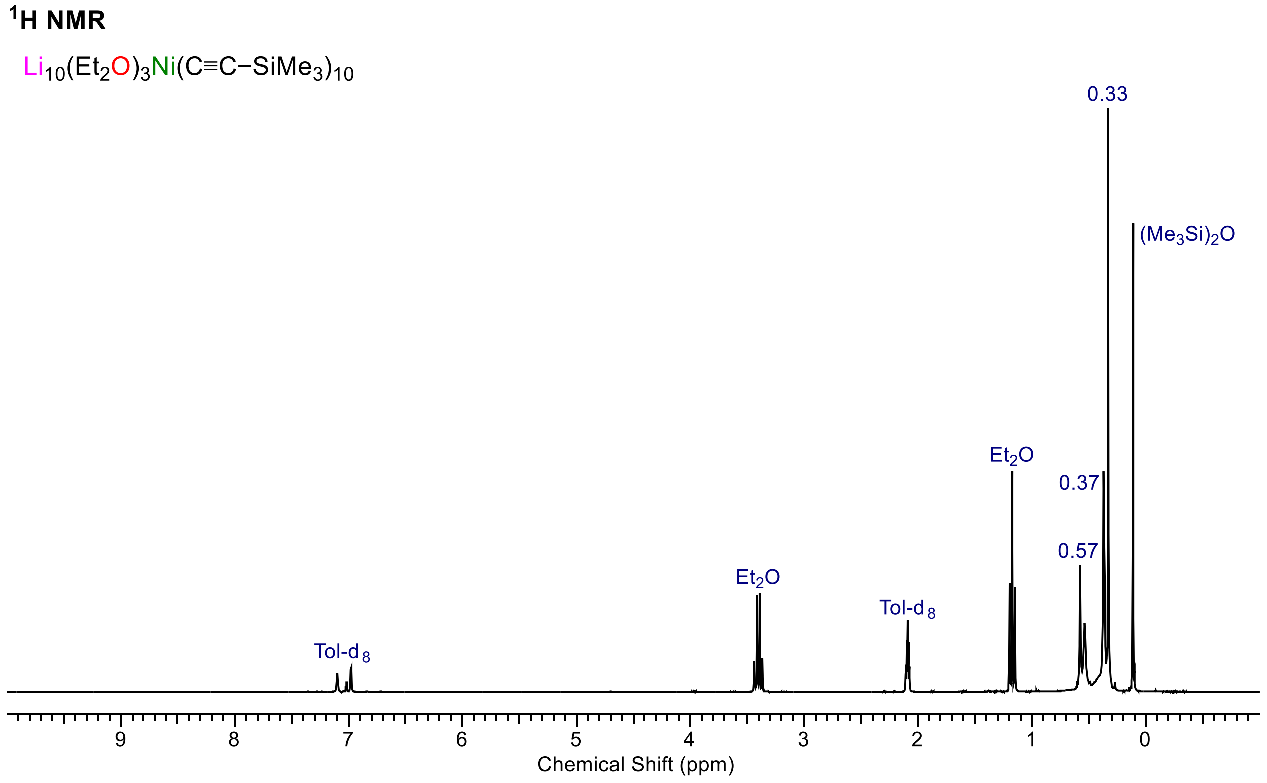

Spectra S6: ^{1}H NMR spectra of $Li_{10}(Et_2O)_3Ni$(C≡C–$SiMe_3$)$_{10}$ (**3**) in toluene-d_8.

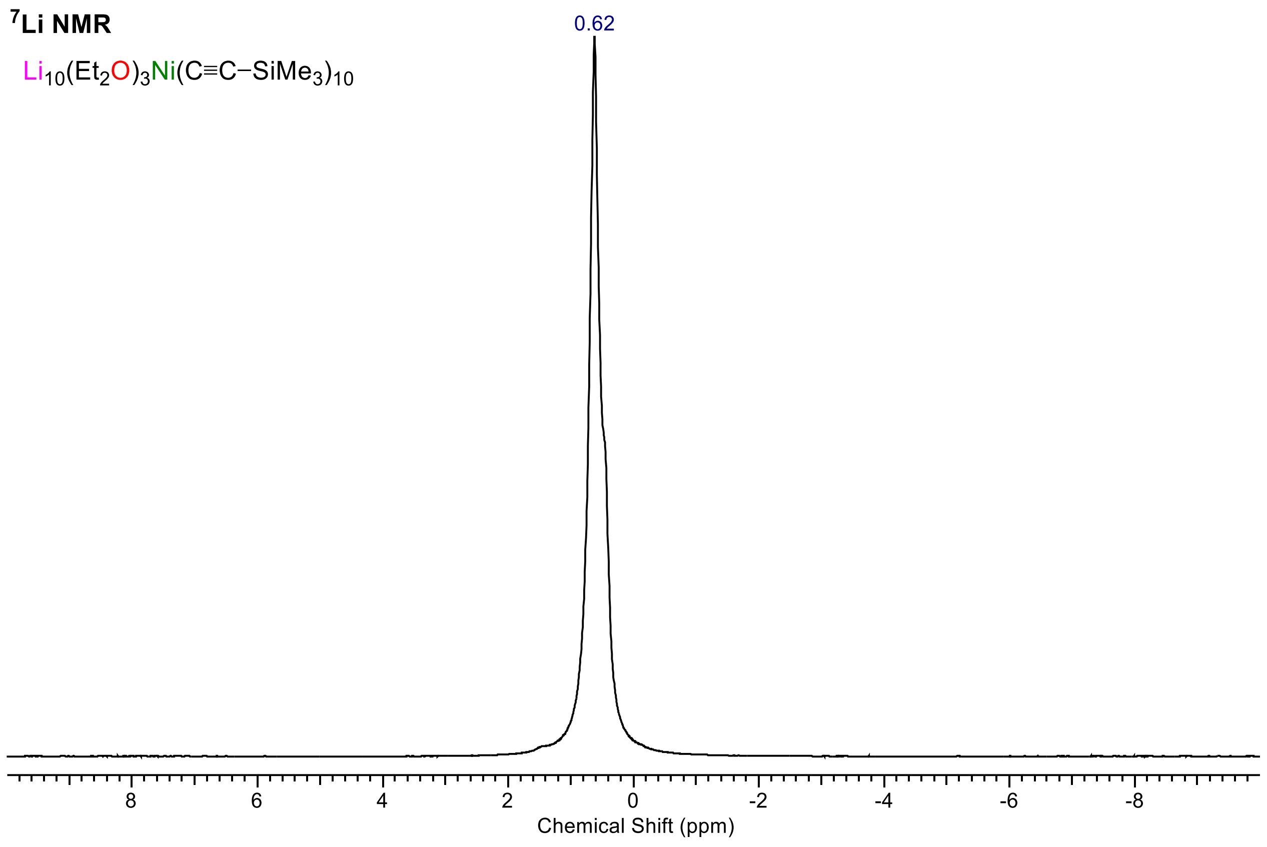

Spectra S7: ^{7}Li NMR spectra of $Li_{10}(Et_2O)_3Ni(C{\equiv}C–SiMe_3)_{10}$ (**3**) in toluene-d$_8$.

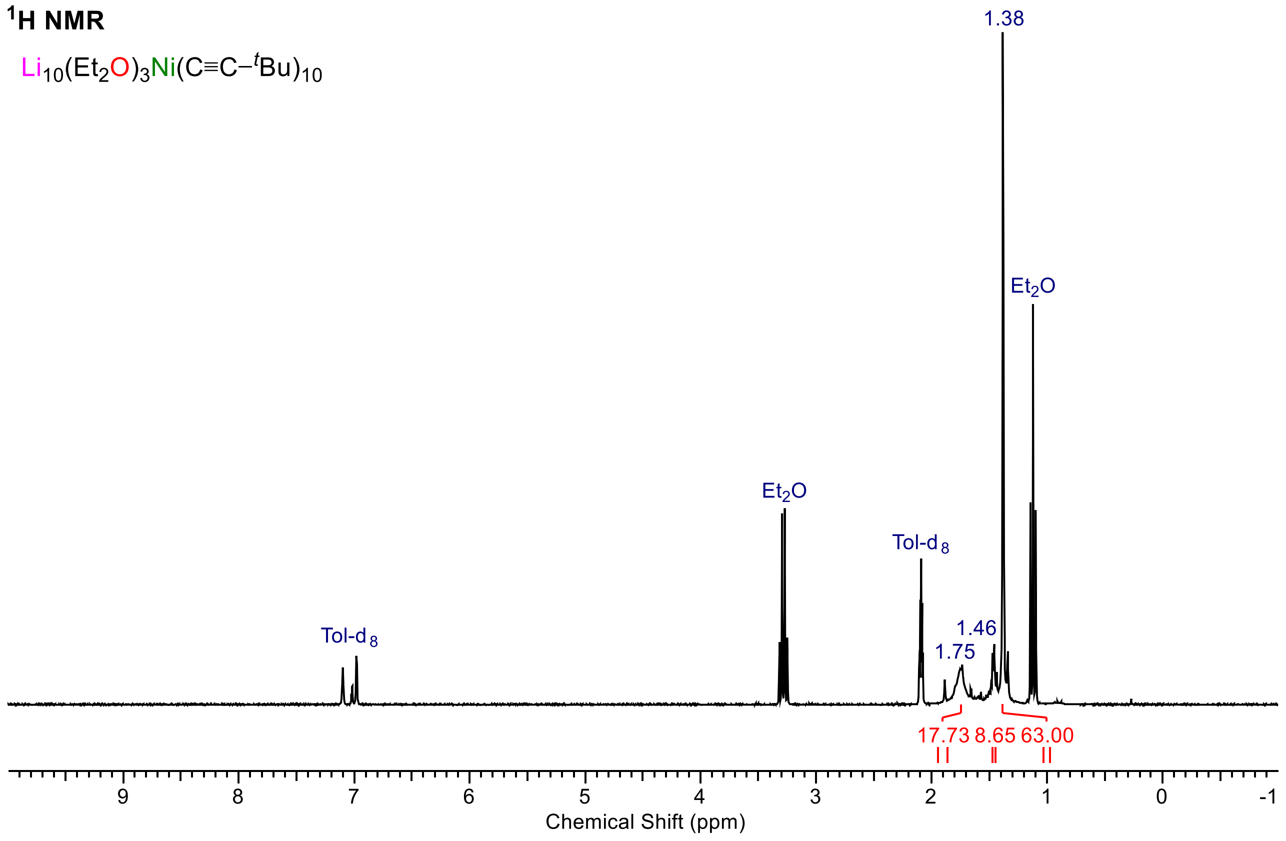

Spectra S8: ^{1}H NMR spectra of $Li_{10}(Et_2O)_3Ni(C{\equiv}C–{}^{t}Bu)_{10}$ (**4**) in toluene-d$_8$.

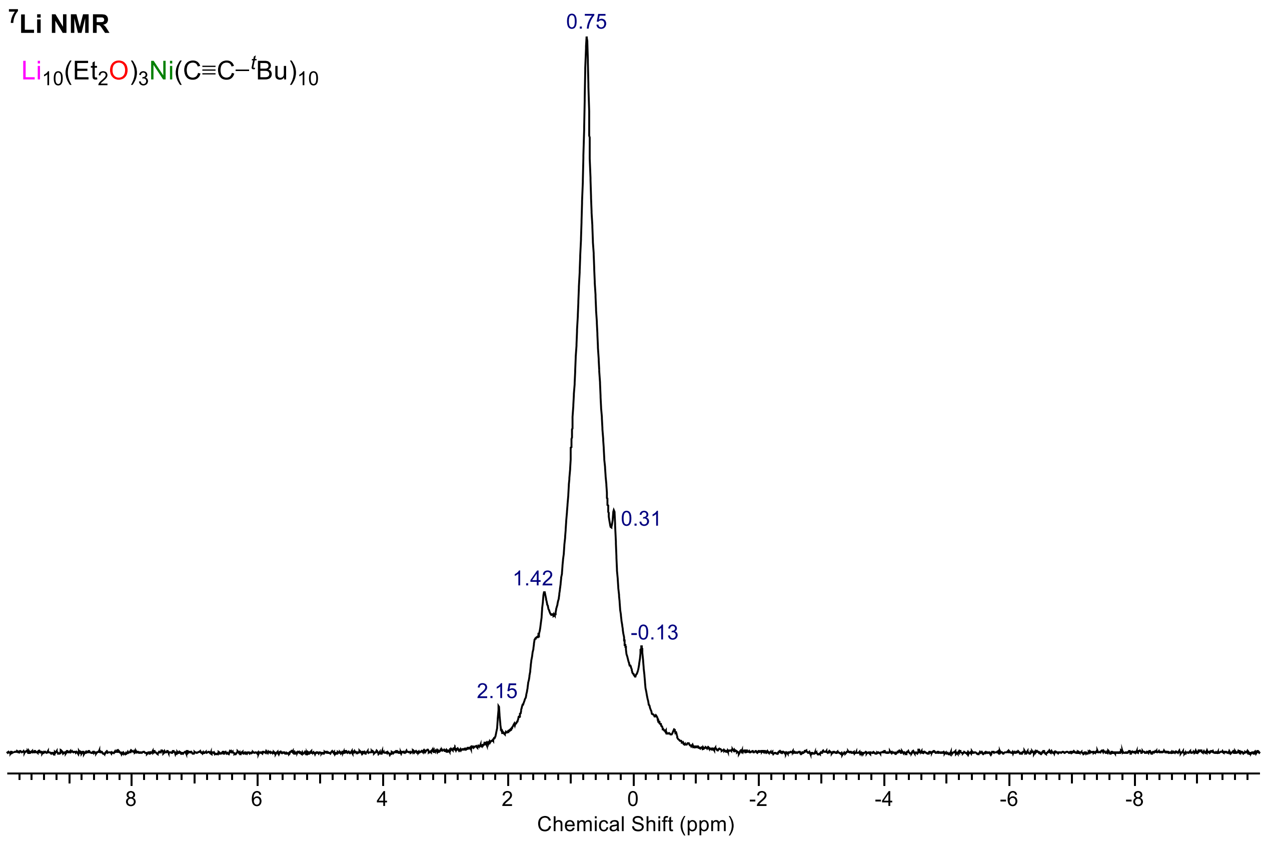

Spectra S9: ^{7}Li NMR spectra of $Li_{10}(Et_2O)_3Ni(C{\equiv}C\text{–}^{t}Bu)_{10}$ (**4**) in toluene-d_8.

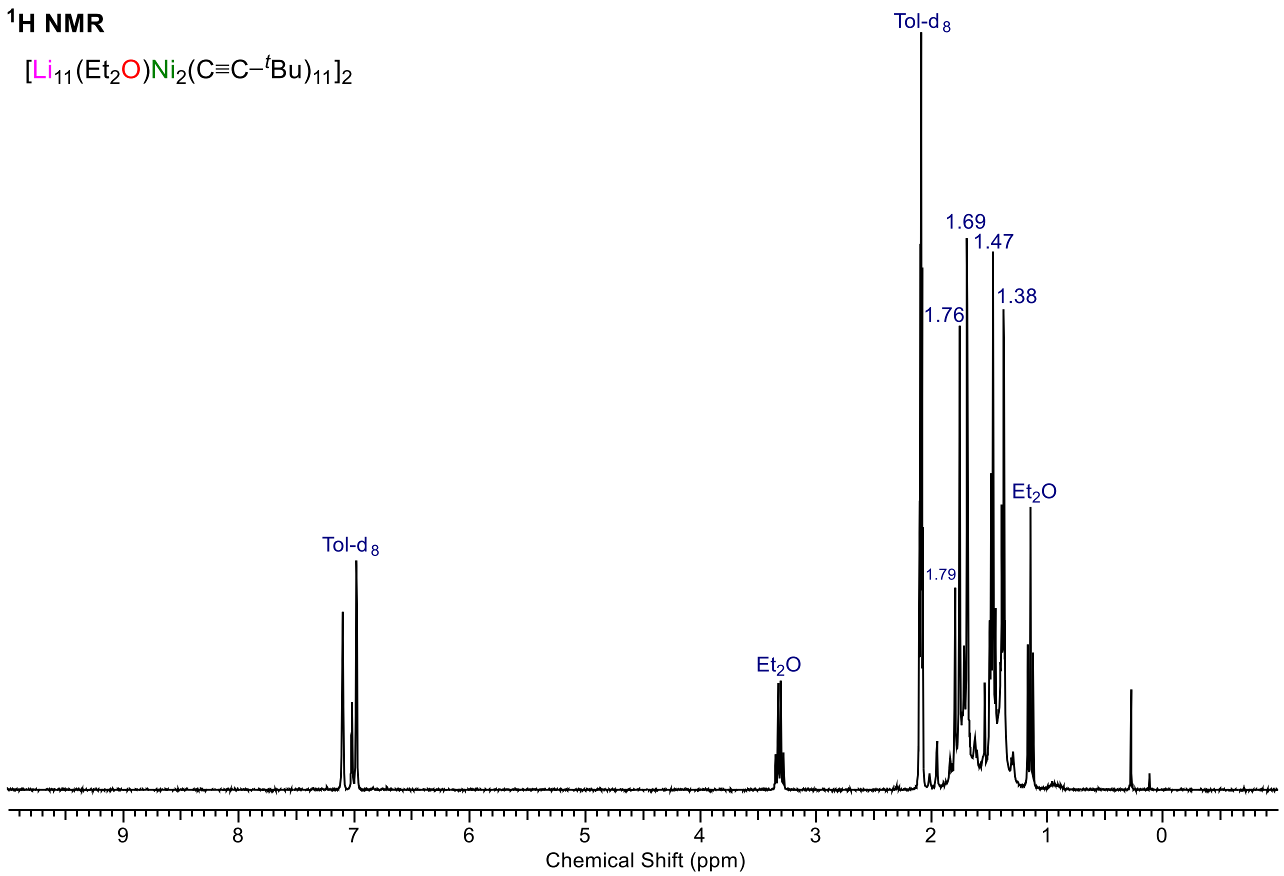

Spectra S10: ^{1}H NMR spectra of $[Li_{11}(Et_2O)Ni_2(C{\equiv}C\text{–}^{t}Bu)_{11}]_2$ (**5**) in toluene-d_8.

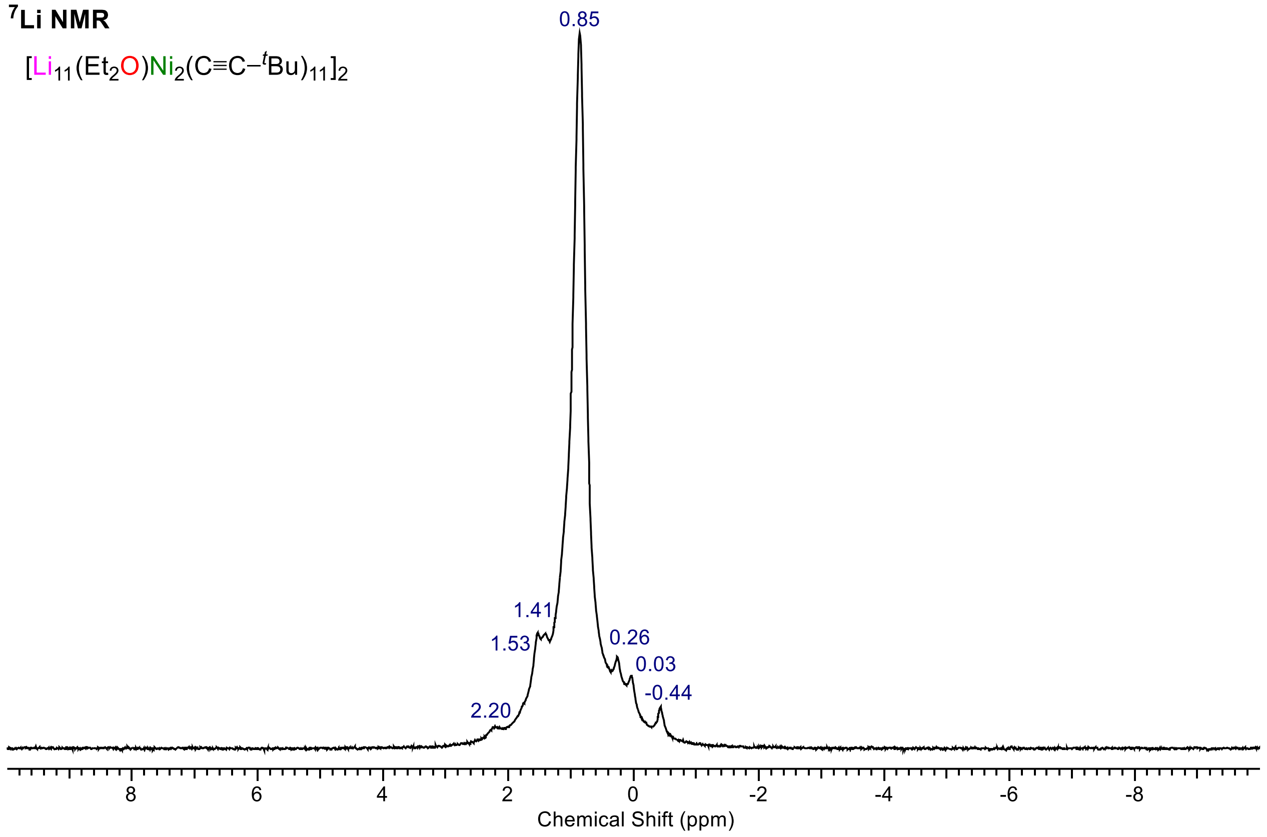

Spectra S11: ^{7}Li NMR spectra of $[Li_{11}(Et_2O)Ni_2(C{\equiv}C–{}^{t}Bu)_{11}]_2$ (**5**) in toluene-d_8.

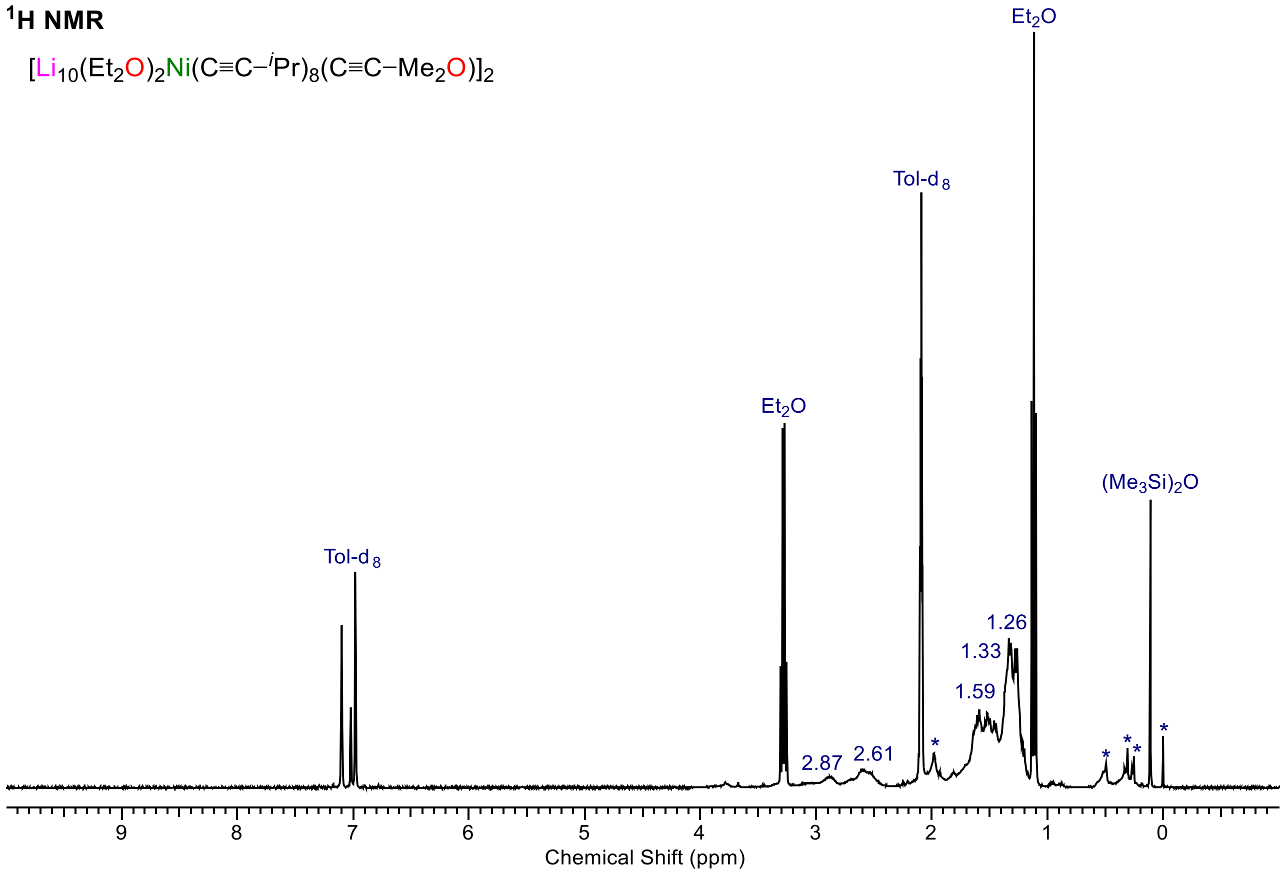

Spectra S12: ^{1}H NMR spectra of $[Li_{10}(Et_2O)_2Ni(C{\equiv}C–{}^{i}Pr)_8(C{\equiv}C–Me_2O)]_2$ (**6**) in toluene-d_8.

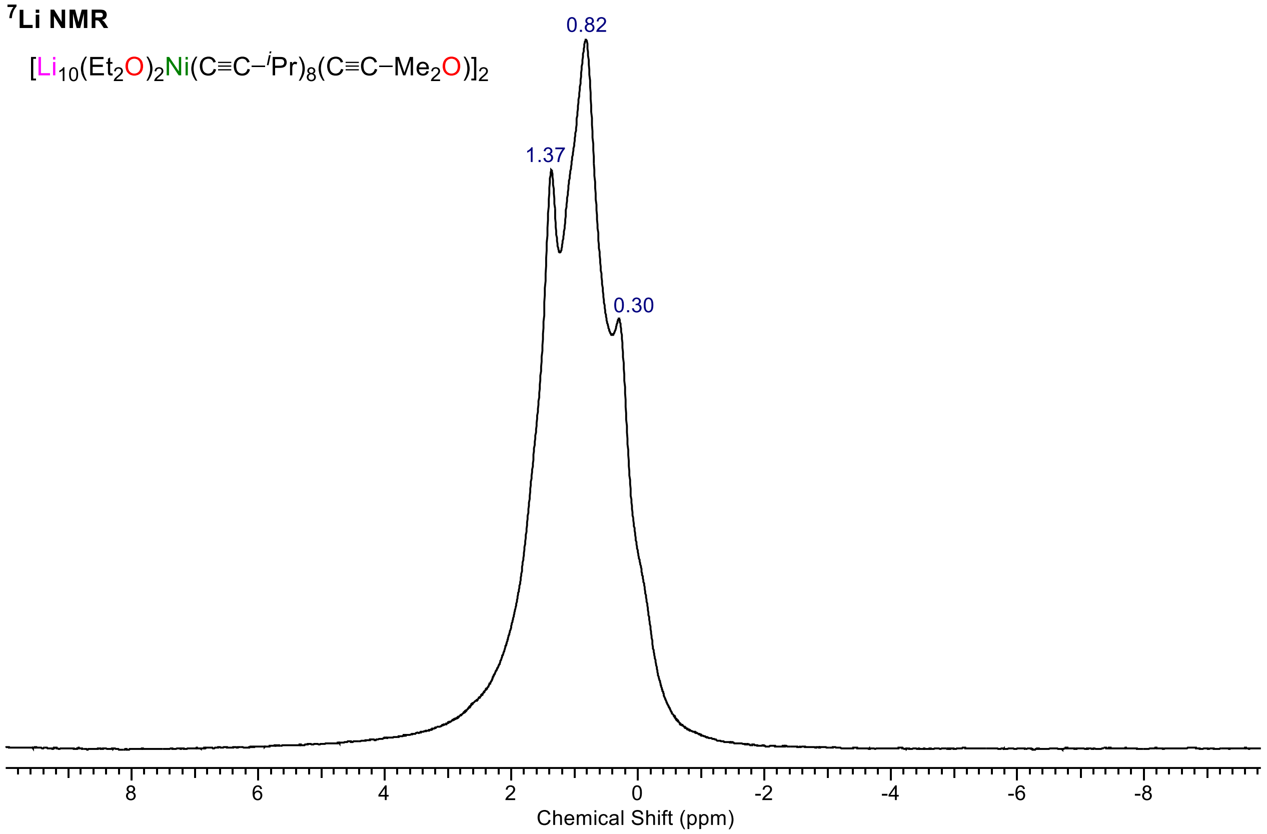

Spectra S13: ^{7}Li NMR spectra of $[Li_{10}(Et_2O)_2Ni(C{\equiv}C–^{i}Pr)_8(C{\equiv}C–Me_2O)]_2$ (**6**) in toluene-d_8.

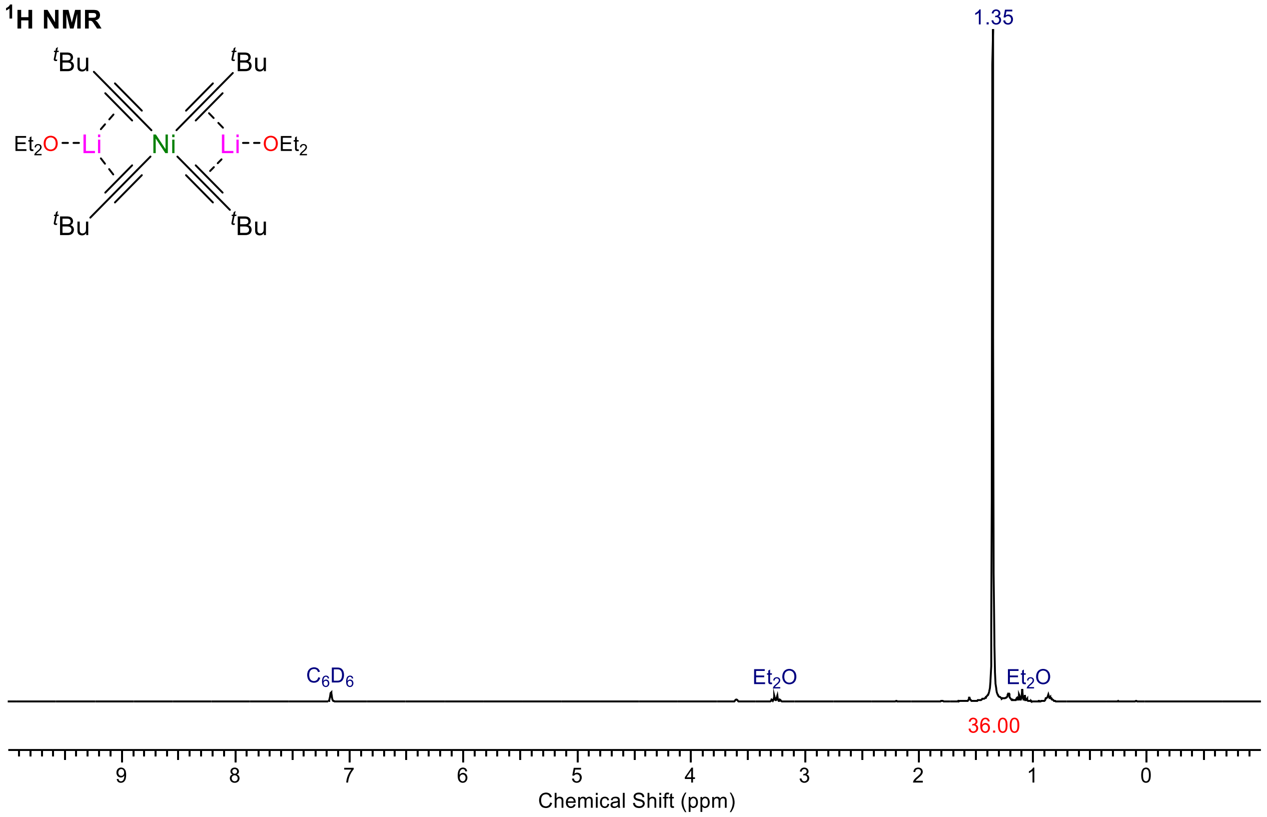

Spectra S14: ^{1}H NMR spectra of $Li_2(Et_2O)_nNi(C{\equiv}C–^{t}Bu)_4$ (**7**) in 5:1 C_6D_6/THF-d_8.

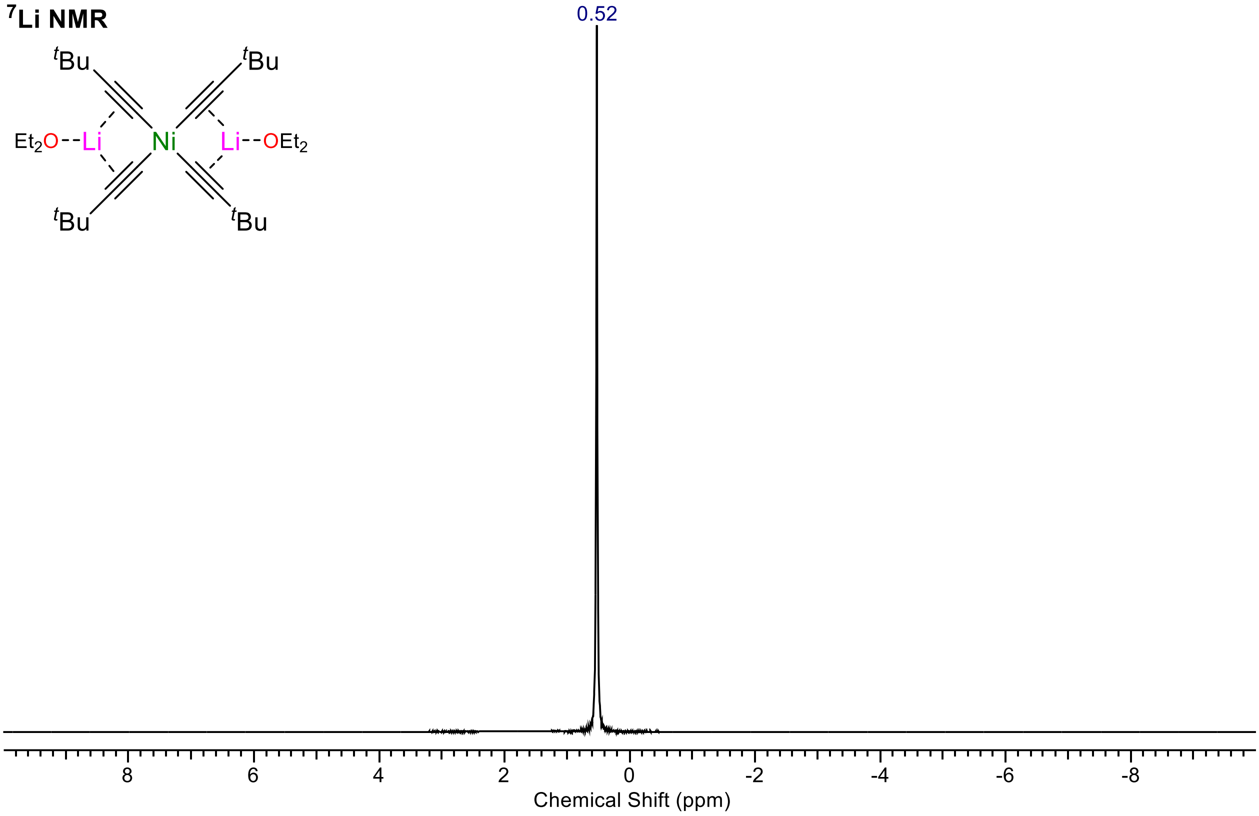

Spectra S15: ^{7}Li NMR spectra of $Li_2(Et_2O)_nNi(C{\equiv}C\text{–}{}^{t}Bu)_4$ (**7**) in 5:1 C_6D_6/THF-d_8.

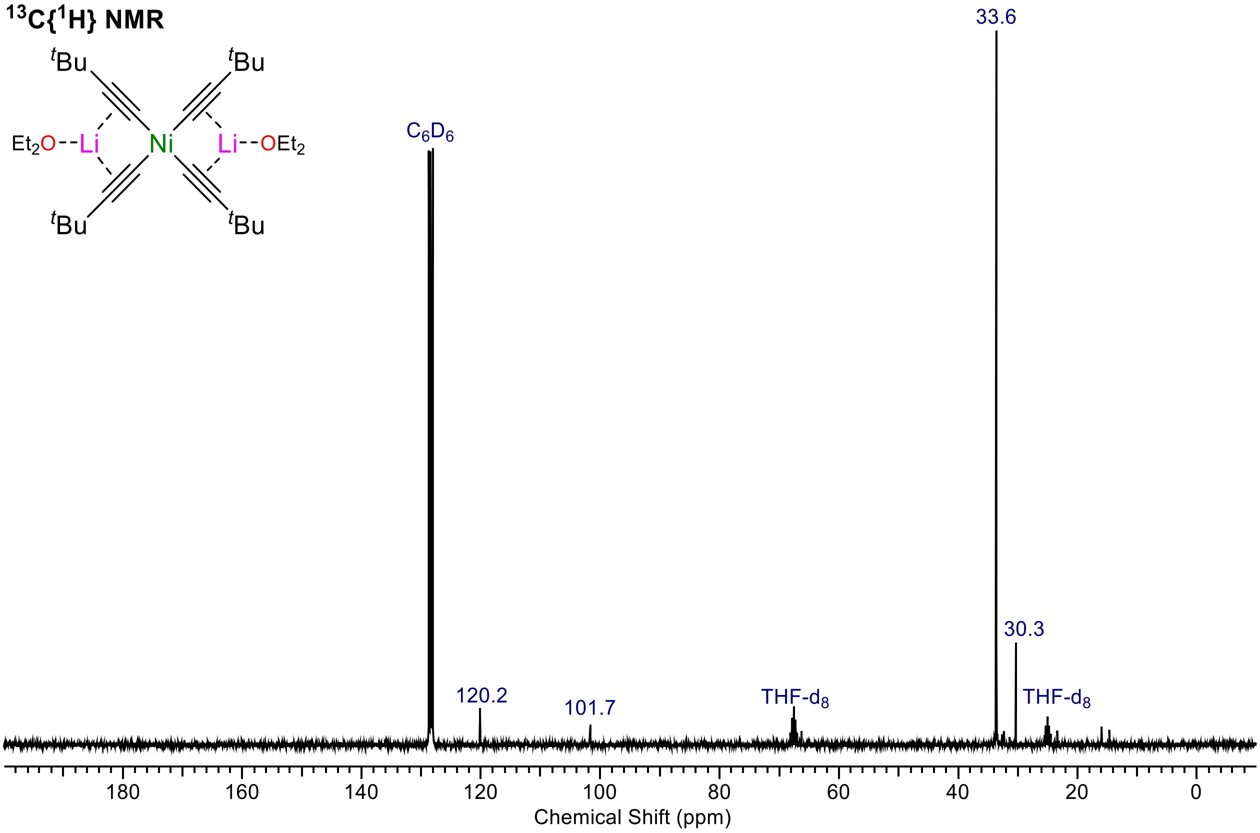

Spectra S16: $^{13}C\{^{1}H\}$ NMR spectra of $Li_2(Et_2O)_nNi(C{\equiv}C\text{–}{}^{t}Bu)_4$ (**7**) in 5:1 C_6D_6/THF-d_8.

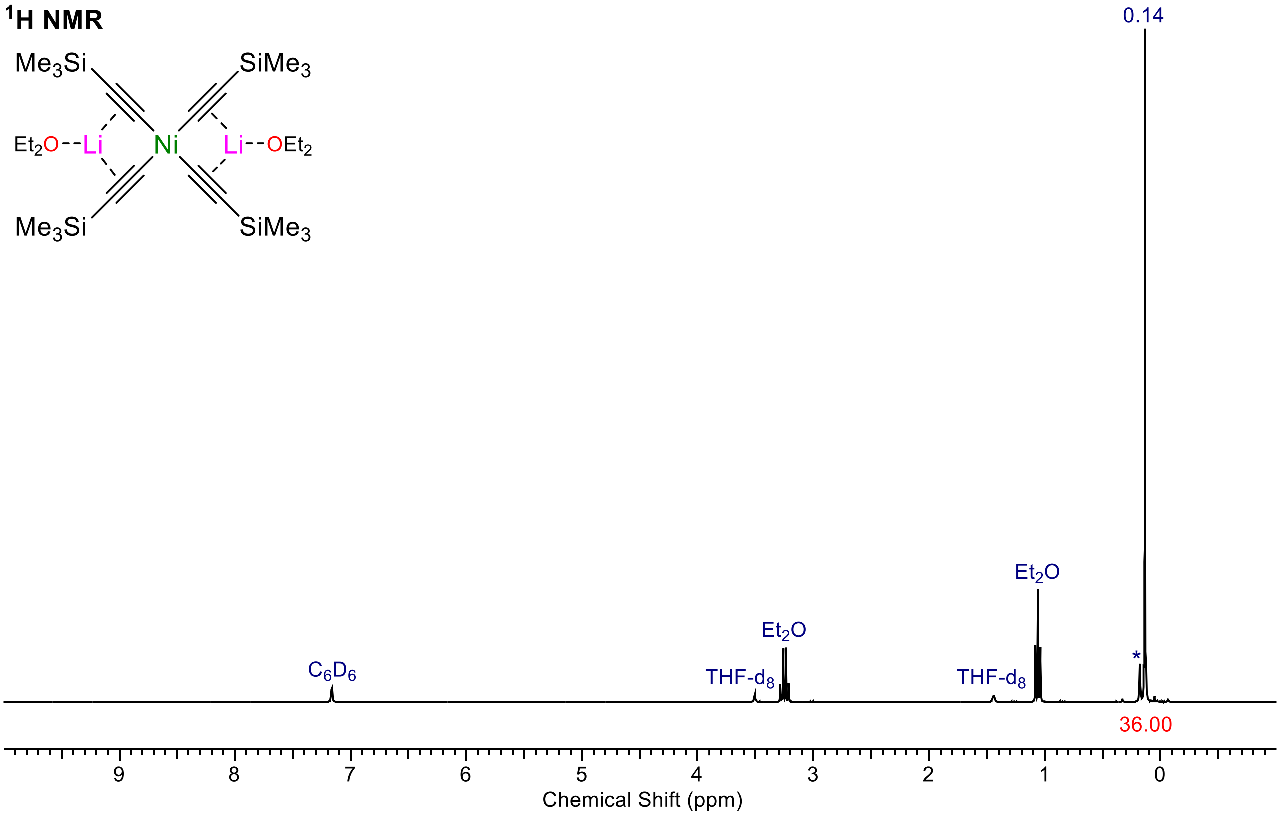

Spectra S17: ^{1}H NMR spectra of $Li_2(Et_2O)_nNi(C\equiv C{-}SiMe_3)_4$ (**8**) in 5:1 C_6D_6/THF-d_8. * Unidentified impurity.

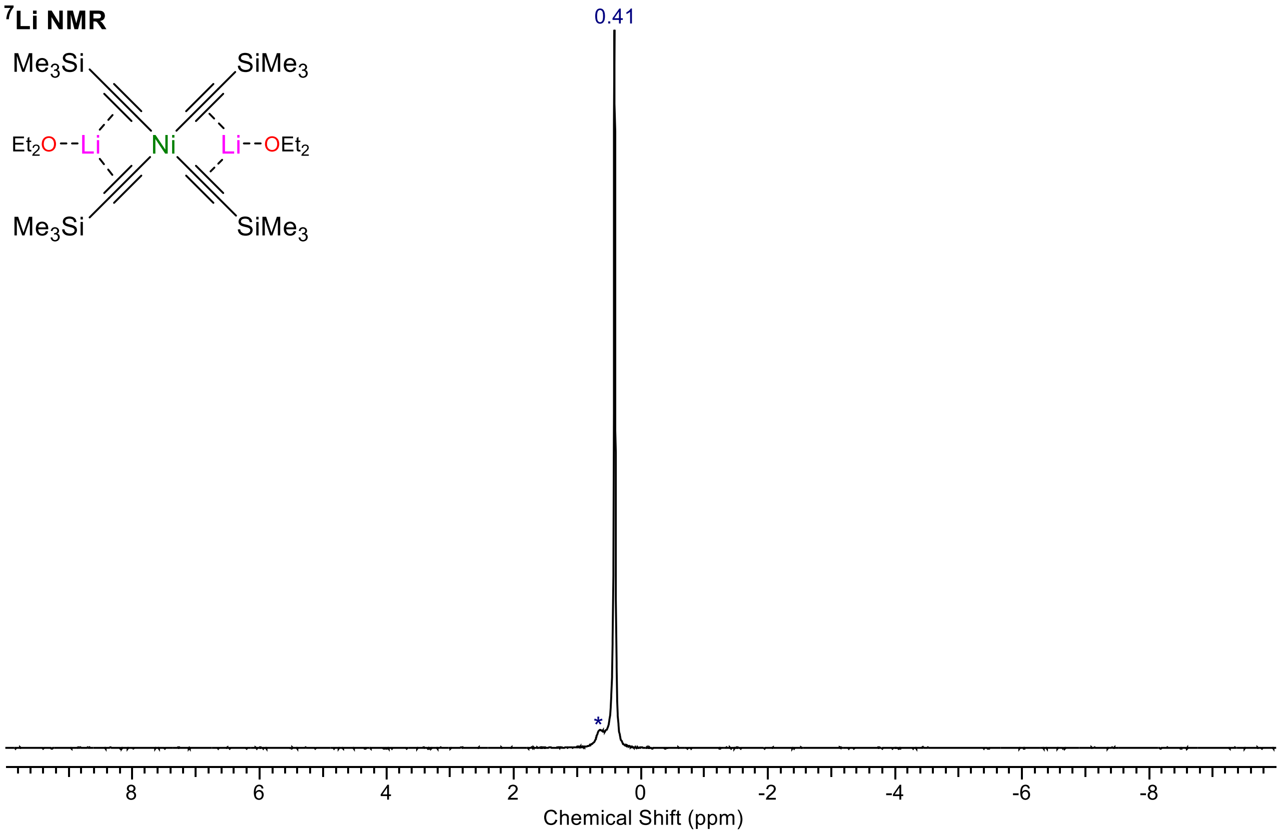

Spectra S18: ^{7}Li NMR spectra of $Li_2(Et_2O)_nNi(C\equiv C{-}SiMe_3)_4$ (**8**) in 5:1 C_6D_6/THF-d_8. * Unidentified impurity.

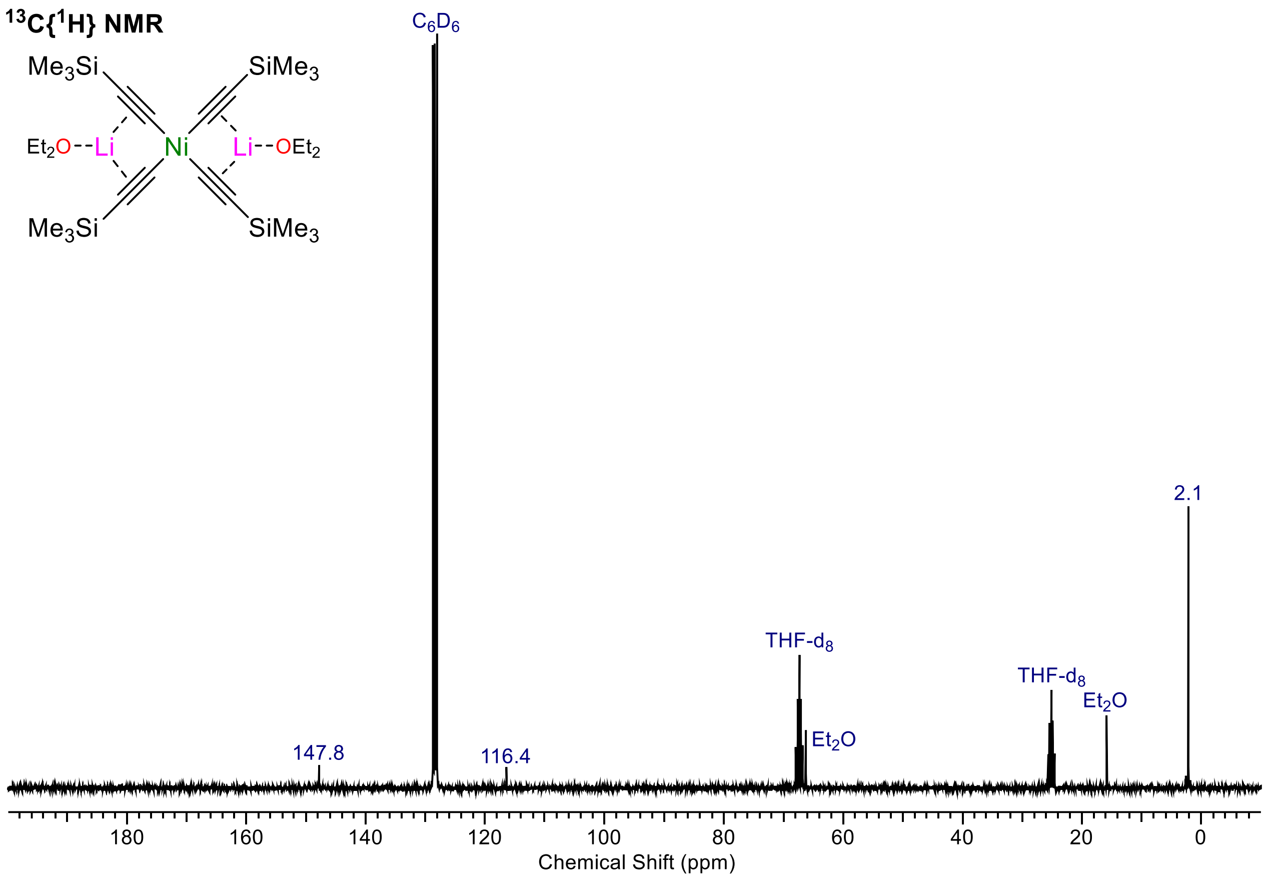

Spectra S19: $^{13}C\{^1H\}$ NMR spectra of $Li_2(Et_2O)_nNi(C{\equiv}C\text{–}SiMe_3)_4$ (**8**) in 5:1 C_6D_6/THF-d_8.

References

1 A. M. Borys, *Organometallics*, 2023, **42**, 182–196.

2 A. M. Borys, The Schlenk Line Survival Guide, https://schlenklinesurvivalguide.com (Accessed April 2023).

3 A. M. Borys, L. A. Malaspina, S. Grabowsky and E. Hevia, *Angew. Chem. Int. Ed.*, 2022, **61**, e202209797.

4 J. Szyling, A. Szymańska, A. Franczyk and J. Walkowiak, *J. Org. Chem.*, 2022, **87**, 10651–10663.

5 R. Neufeld and D. Stalke, *Chem. Sci.*, 2015, **6**, 3354–3364.

6 S. Bachmann, R. Neufeld, M. Dzemski and D. Stalke, *Chem. Eur. J.*, 2016, **22**, 8462–8465.

7 S. Bachmann, B. Gernert and D. Stalke, *Chem. Commun.*, 2016, **52**, 12861–12864.

8 W. Bauer and D. Seebach, *Helv. Chim. Acta*, 1984, **67**, 1972–1988.

9 G. Fraenkel and P. Pramanik, *J. Chem. Soc. Chem. Commun.*, 1983, 1527–1529.

10 A. C. Jones, A. W. Sanders, M. J. Bevan and H. J. Reich, *J. Am. Chem. Soc.*, 2007, **129**, 3492–3493.

11 Oxford-Diffraction, 2018.

12 G. M. Sheldrick, *Acta Cryst.*, 2015, **A71**, 3–8.

13 G. M. Sheldrick, *Acta Cryst.*, 2015, **C71**, 3–8.

14 O. V. Dolomanov, L. J. Bourhis, R. J. Gildea, J. A. K. Howard and H. Puschmann, *J. Appl. Crystallogr.*, 2009, **A42**, 339–341.
